# Decoding migraine disorders: parathyroid hormone-related peptide receptors as key genetic drivers

**DOI:** 10.1093/braincomms/fcaf142

**Published:** 2025-04-28

**Authors:** Andreia Dias, Marta Ferreira, Mariana Santos, Alda Sousa, Carla Oliveira, Miguel Alves-Ferreira, Carolina Lemos

**Affiliations:** i3S—Instituto de Investigação e Inovação em Saúde, Universidade do Porto, Porto 4200-135, Portugal; Unidade Multidisciplinar de Investigação Biomédica (UMIB), Instituto de Ciências Biomédicas Abel Salazar (ICBAS), Universidade do Porto, Porto 4050-313, Portugal; ITR—Laboratory for Integrative and Translational Research in Population Health, Porto 4050-600, Portugal; i3S—Instituto de Investigação e Inovação em Saúde, Universidade do Porto, Porto 4200-135, Portugal; IPATIMUP—Instituto de Patologia e Imunologia Molecular da Universidade do Porto, Universidade do Porto, Porto 4200-135, Portugal; Unidade Multidisciplinar de Investigação Biomédica (UMIB), Instituto de Ciências Biomédicas Abel Salazar (ICBAS), Universidade do Porto, Porto 4050-313, Portugal; ITR—Laboratory for Integrative and Translational Research in Population Health, Porto 4050-600, Portugal; IBMC—Instituto de Biologia Molecular e Celular, Universidade do Porto, Porto 4200-135, Portugal; i3S—Instituto de Investigação e Inovação em Saúde, Universidade do Porto, Porto 4200-135, Portugal; Unidade Multidisciplinar de Investigação Biomédica (UMIB), Instituto de Ciências Biomédicas Abel Salazar (ICBAS), Universidade do Porto, Porto 4050-313, Portugal; i3S—Instituto de Investigação e Inovação em Saúde, Universidade do Porto, Porto 4200-135, Portugal; IPATIMUP—Instituto de Patologia e Imunologia Molecular da Universidade do Porto, Universidade do Porto, Porto 4200-135, Portugal; FMUP—Faculdade de Medicina da Universidade do Porto, Universidade do Porto, Porto 4200-319, Portugal; Unidade Multidisciplinar de Investigação Biomédica (UMIB), Instituto de Ciências Biomédicas Abel Salazar (ICBAS), Universidade do Porto, Porto 4050-313, Portugal; IBMC—Instituto de Biologia Molecular e Celular, Universidade do Porto, Porto 4200-135, Portugal; CGPP—Centro de Genética Preditiva e Preventiva, Universidade do Porto, Porto 4200-135, Portugal; i3S—Instituto de Investigação e Inovação em Saúde, Universidade do Porto, Porto 4200-135, Portugal; Unidade Multidisciplinar de Investigação Biomédica (UMIB), Instituto de Ciências Biomédicas Abel Salazar (ICBAS), Universidade do Porto, Porto 4050-313, Portugal; ITR—Laboratory for Integrative and Translational Research in Population Health, Porto 4050-600, Portugal

**Keywords:** gene expression, migraine disorders, G-protein-coupled receptors, parathyroid hormone-related peptide receptor, parathyroid hormone

## Abstract

Migraine is a complex neurological disorder, and the most common migraine categories are migraine with aura and without aura. The higher prevalence of migraine in related individuals compared to the general population indicates a potential genetic predisposition; however, gene expression, which is influenced by both genetic and environmental factors, can also be a major factor in the migraine susceptibility. Given the high number of Portuguese migraine patients whose diagnosis and treatment have not yet been well established, we decided to carry out a whole transcriptome analysis within a migraine Portuguese cohort. This study aims to identify potential biomarkers that could contribute to improved migraine therapy. We performed total RNA sequencing on whole blood samples from 15 migraine patients and 12 age-matched controls. Differential expression analysis and gene set enrichment analysis were performed in different migraine subgroups. Finally, we performed the protein-protein interaction networks of differentially expressed genes. Gene set enrichment analysis comparing migraine patients with controls highlighted upregulated pathways linked to metabolism, and downregulated immuno-inflammatory pathways. Moreover, the groups of female migraine patients and female migraine without aura patients emphasized significant upregulated pathways, including G protein-coupled receptors signalling pathways, when compared with female controls. Interestingly, we found two important differentially expressed genes related to parathyroid hormone: *PTH1R* and *PTH2*. *PTH1R* was upregulated in female migraine without aura versus female controls, while *PTH2* was both upregulated between female migraine patients and female controls, as well as between female migraine without aura and controls. Here, we show, for the first time, the involvement of parathyroid hormone receptors and their associated gene expression patterns in female migraine patients. These molecules stand out as sturdy and promising biomarkers for innovative therapeutic in female migraine patients.

## Teaser

Dias *et al*. analysed gene expression in Portuguese migraine patients, revealing upregulated metabolic and G-protein coupled receptor pathways and downregulated immune pathways. Notably, *PTH1R* and *PTH2* were upregulated in female migraine groups, highlighting parathyroid hormone receptors as potential biomarkers for female migraine patients.

## Graphical Abstract

**Graphical Abstract:**
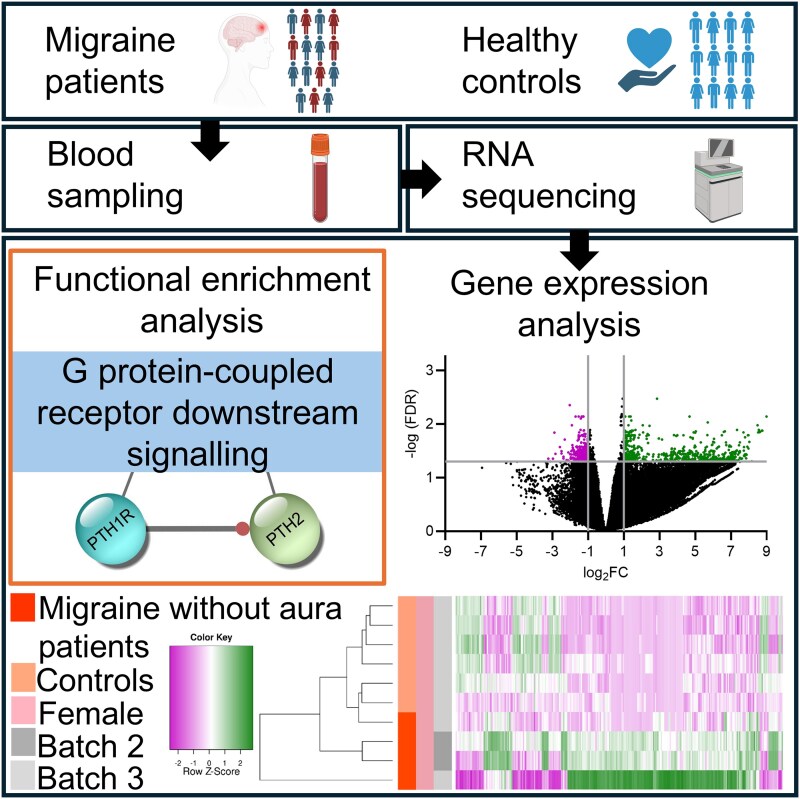

## Introduction

Migraine is a complex neurological disorder classified into two main categories: migraine with aura (MwA) or without aura (MwoA). Aura is characterized by neurological transitory visual, sensory, aphasic or motor symptoms.^1^

Despite recent significant advances in migraine research, several critical questions about the condition persist. One such question concerns the underdiagnosis and inadequate treatment of migraine. This is likely because many patients opt to self-diagnose or avoid seeking professional care. Additionally, non-specialist healthcare providers frequently misdiagnose migraine as other conditions. The most common misdiagnoses include sinusitis, allergies, cervical pain syndrome and tension-type headache.^2^ Thus, current treatment options in migraine frequently result in unsatisfactory outcomes, rendering it one of the most undertreated neurological disorders.^3^

Understanding why certain individuals are more prone to developing migraine than others remain a primary challenge, which we believe can be addressed by considering their genetic background. Documented evidence suggests a familial component to migraine, with heritability estimates ranging from 34 to 64%.^4^ While migraine has a genetic component, various environmental and lifestyle factors are also linked to the condition. Triggers, such as light sensitivity and weather changes, have consistently been identified as catalysts for migraine attacks.^5^ Indeed, while shared environmental risk factors within families might contribute to the onset of migraine, the higher prevalence of migraine in related individuals compared to the general population indicates a potential genetic predisposition.^6^

Genetic germline variations tend to remain relatively stable over a lifetime, whereas gene expression varies by tissue and can be influenced by both genetic and environmental factors.^7^

Individuals carry millions of DNA variants in their genome,^8^ with a significant portion located in non-coding regions, impacting gene expression and splicing patterns.^9^ RNA-sequencing (RNA-seq) enables the quantification of gene expression levels, identification of novel transcripts, alternative splicing and gene fusion events all potentially associated with migraine mechanisms.^10^

While recent transcriptomic studies using RNA-seq have shown promise in the context of pain, identifying potential pain-related genes and variations in gene expression,^11^ there is a dearth of similar data concerning patients with migraine.^12^

Our aim is to conduct a whole transcriptome analysis with total RNA-seq for the first time within a Portuguese cohort of migraine patients. Implementing these methodologies holds the promise of translating research findings into effective therapies by exploring the interaction between environmental factors and epigenetic regulation of gene expression.^13^

## Material and methods

### Study participants

This study was conducted in a Portuguese cohort from the outpatient neurologic clinic at Centro Hospitalar Universitário de Santo António (CHUdSA) and at Centre for Predictive and Preventive Genetics (CGPP-i3S). We selected a homogeneous group of 15 episodic migraine patients (9 patients with MwoA and 6 patients with MwA) and 12 matched controls. Clinical information of participants was collected, and patients with hemiplegic and chronic migraine were excluded; those with the co-occurrence of MwA and MwoA were included in the MwA group. Controls and cases were from the same ethnic and geographical origin, age-matched and non-related. All cases and controls were evaluated by the neurologist at the medical consultation, where a questionnaire was carried out according to the operational criteria of the International Headache Society (IHS)-3rd edition of the International Classification of Headache Disorders.^1^ Along with establishing an accurate diagnosis, the questionnaire gathered detailed information on symptom characteristics, attack frequency, medication usage, treatment responses and associated comorbid conditions. Blood samples were collected in the sequence of a neurologic appointment and were kept at CGPP’s biobank at i3S.

The Ethics Committee of CHUdSA and i3S approved the study and participants gave their written informed consent complying with the World Medical Association Declaration of Helsinki. Institutional Review Board Statement: The use of biological material and information from patients was approved by the Committee for Ethical and Responsible Conduct of Research CECRI, i3S; approval code 2/CECRI/2020.

### Sample collection and extraction

Peripheral blood samples were collected into Tempus^TM^ Blood RNA tubes (Applied Biosystems, Foster City, CA, USA). Total RNA was extracted with the MagMAX for Stabilized Blood Tubes RNA Isolation Kit (Thermo Fisher Scientific) according to the manufacturer’s protocol. Extracted RNA was quantified using Qubit® RNA HS Assay Kit on a Qubit®2.0 Fluorometer (Thermo Fisher Scientific Inc., Waltham, MA, USA) and the purity and integrity by the Experion (Bio-Rad Laboratories, USA; RIN > 7).

### RNA sequencing

For whole-transcriptome library preparation, the TruSeq Stranded Total RNA with Ribo-Zero Globin library kit (Illumina, Inc., San Diego, CA, USA) and paired-end sequencing were performed using the Illumina NovaSeq platform with a 2× 150 read length. Quality control of the raw fastq files was performed using FastQC, unique reads were mapped to the human genome (GRCh38) reference sequence using STAR. RSeQC was used to count and annotated the read counts using information from USCS GRCh38 (Genecode v41) and ensemble (v107 and v108).

### Statistical analysis

For differential expression analysis, we compared migraine patients (MwoA and MwA) to healthy controls, MwoA versus healthy controls, MwA versus healthy controls and MwoA versus MwA. Differentially expressed genes (DEGs) were detected using edegR (v.3.38.4) R package and DEGs were selected considering the canonical transcripts, |log2fold-change|≥ 1 and adjusted *P*-value < 0.05. The heatmap of DEGs were generated using the heatmap.2 function from gplots R package (v.3.1.3). Volcano plots were generated using GraphPad Prism 8.4.3 software.

A Venn diagram in Venny 2.1 (https://bioinfogp.cnb.csic.es/tools/venny/) was used to show the common DEGs between comparison subgroups.

Additionally, we performed Gene Set Enrichment Analysis (GSEA), implemented in the WEB-based GEne SeT AnaLysis Toolkit (Webgestalt) software (https://www.webgestalt.org/). The parameters were set as following: ‘‘statistical method: hypergeometric; multiple test adjustment: BH; significance-level: *P* < 0.05; minimum number of five genes and a maximum number of 2000 genes per gene set by default. The analyses are based on the estimation of likelihood ratio (LR) for each gene with the respective *P*-values.

### Pathway analysis

The protein–protein interaction networks of DEGs in the present study were constructed using the Search Tool for the Retrieval of Interacting Genes (STRING; version 12.0; http://string-db.org/cgi/input.pl) database. The DEGs involved in the Reactome pathways of the GSEA analysis methods were imported. A confidence score >0.7 was used as the cut-off criterion. Then, Cytoscape software (version 3.10.1; http://www.cytoscape.org/) was used to construct the protein–protein interaction network.

## Results

### Sample characterization

A whole-transcriptome analysis was conducted across various subgroups: migraine patients versus healthy controls, MwoA patients versus controls, MwA patients versus controls and MwoA versus MwA. Notably, the MwA group exhibited a slightly higher mean age compared to the MwoA group. Nonetheless, the migraine subgroups displayed similarities in attack frequency, type of headache pain, and family history (as detailed in Table 1). It is worth mentioning that all MwA patients experienced visual auras during their attacks, with one patient reporting sensory changes, specifically tingling and numbness on the left side of the face. The 12 healthy controls had a mean age of 54.83 ± 20.14, with no significant age difference observed between migraine patients (51.60 ± 16.23) and controls (54.83 ± 20.14). The ratio of female:male was 1,1:1.

**Table 1:** Descriptive characteristics of MwoA and MwA patients

Type of migraine	MwoA (*n* = 9)	MwA (*n* = 6)
**Age**	48.22 (±19.01)	56.67 (±10.41)
**Age of onset (n/total)**		
0–10 (inclusive)	0/9	1/6
11–20 (inclusive)	7/9	3/6
21–30 (inclusive)	1/9	1/6
31–40 (inclusive)	1/9	1/6
**Attack frequency (attacks/month) (n/total)**		
<1/month	3/9	2/6
1–15/month	6/9	4/6
**Type of headache pain (n/total)**		
Throbbing/pulsatile	9/9	6/6
**Family history (n/total)**	9/9	6/6
**Recurrent medication (n/total)**	3/9	3/6
**Trigger factors (n/total)**		
Food and drinks	1/9	2/6
Skip meals	0/9	1/6
Tiredness	1/9	0/6
Nausea	5/9	5/6
Vomiting	2/9	3/6
Photophobia	7/9	4/6
Phonophobia	5/9	4/6
Stress	2/9	2/6
Menstruation	4/9	2/6
Fragrances	1/9	3/6
**Comorbidities (n/total)**		
Autoimmune diseases	0/9	2/6
Epilepsy	1/9	0/6
Endocrine and metabolic	2/9	0/6
Gastrointestinal	1/9	0/6
Cardiac disorders	1/9	1/6

### Differential expression analysis

**Figure 1:**
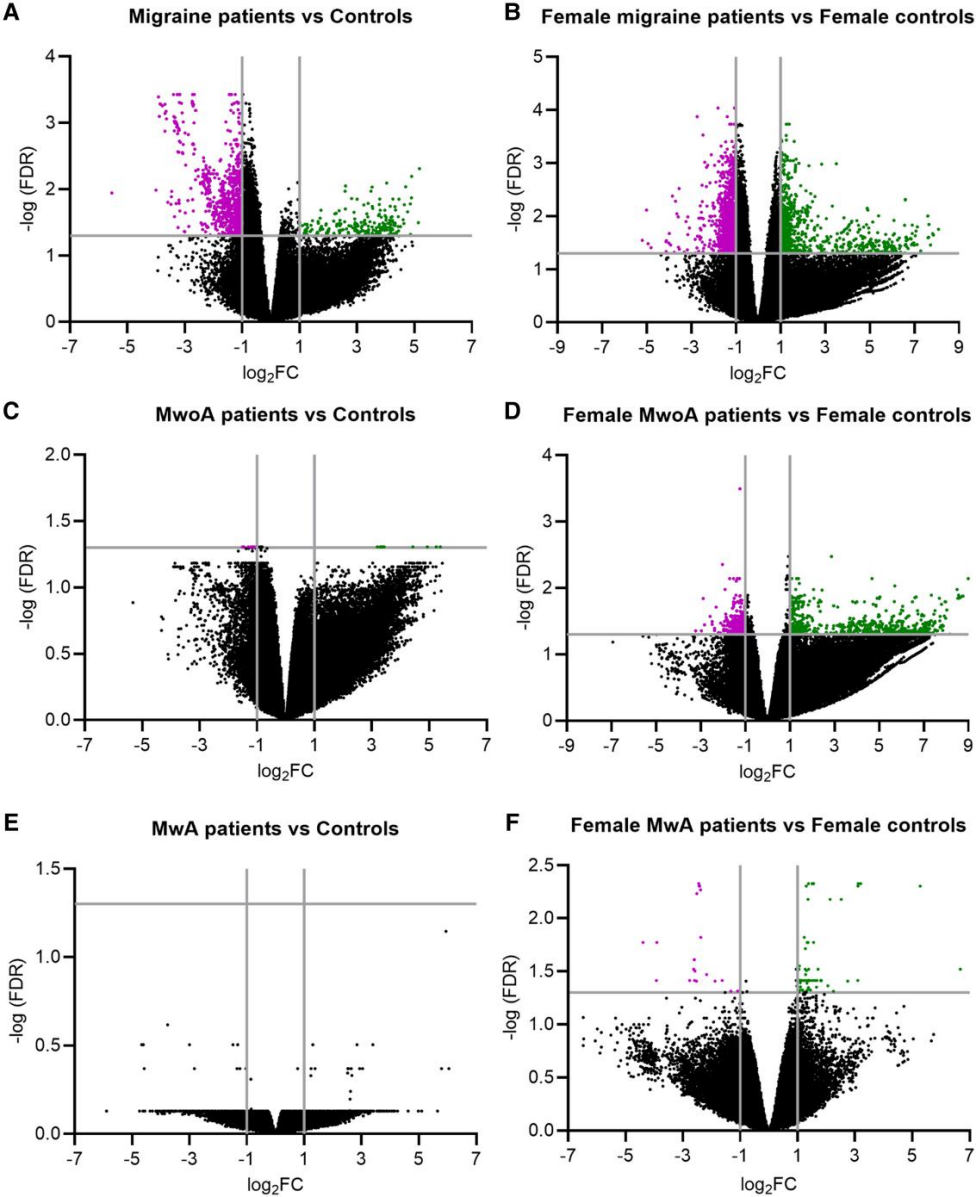
**Distribution of DEGs between different migraine groups and controls.** Volcano plots of DEGs between migraine patients and controls (*N* = 27) (**A**), female migraine patients and female controls (*N* = 14) (**B**), MwoA patients and controls (*N* = 21) (**C**), female MwoA patients and controls (*N* = 10) (**D**), MwA patients and controls (*N* = 18) (**E**), female MwA patients and controls (*N* = 10) (**F**). Volcano plot displaying individual genes, with a significant threshold of |log_2_FC| ≥ 1 and adjusted *P*-value < 0.05. Magenta and green dots represent downregulated and upregulated DEGs, respectively.

All samples passed quality control during the sequencing process, with no samples excluded. After that, we performed a differential expression analysis, in which we found 282 DEGs between migraine patients and controls (*P* < 0.05, |log2FC|≥1; Fig. 1A; supplementary table 1). When we performed the analyses for MwoA and MwA groups separately, we only found 5 DEGs between MwoA and controls (*P* < 0.05, |log2FC|≥1; Fig. 1C; supplementary table 2), and no DEGs were found when comparing MwA versus controls (Fig. 1E) nor for MwoA versus MwA (supplementary Fig. 1A). Additional analyses were carried out by gender. In this sense, 545 DEGs were found between female migraine patients and female controls (*P* < 0.05, |log2FC|≥1; Fig. 1B; supplementary table 3), 292 DEGs between female MwoA patients and female controls (*P* < 0.05, |log2FC|≥1; Fig. 1D;  supplementary table 4) and 27 DEGs between female MwA patients and female controls (*P* < 0.05, |log2FC|≥1; Fig. 1F;  supplementary table 5). In contrast, no DEGs were found between female MwA and female MwoA (supplementary Fig. 1B). Regarding males, DEGs were not identified in our analysis (supplementary Fig. 1C and D), except when comparing male MwA patients versus male controls (supplementary Fig. 1E; supplementary table 6), and male MwoA versus male MwA patients (supplementary table 7) but the small sample size did not allow us to draw significant conclusions.

**Figure 2:**
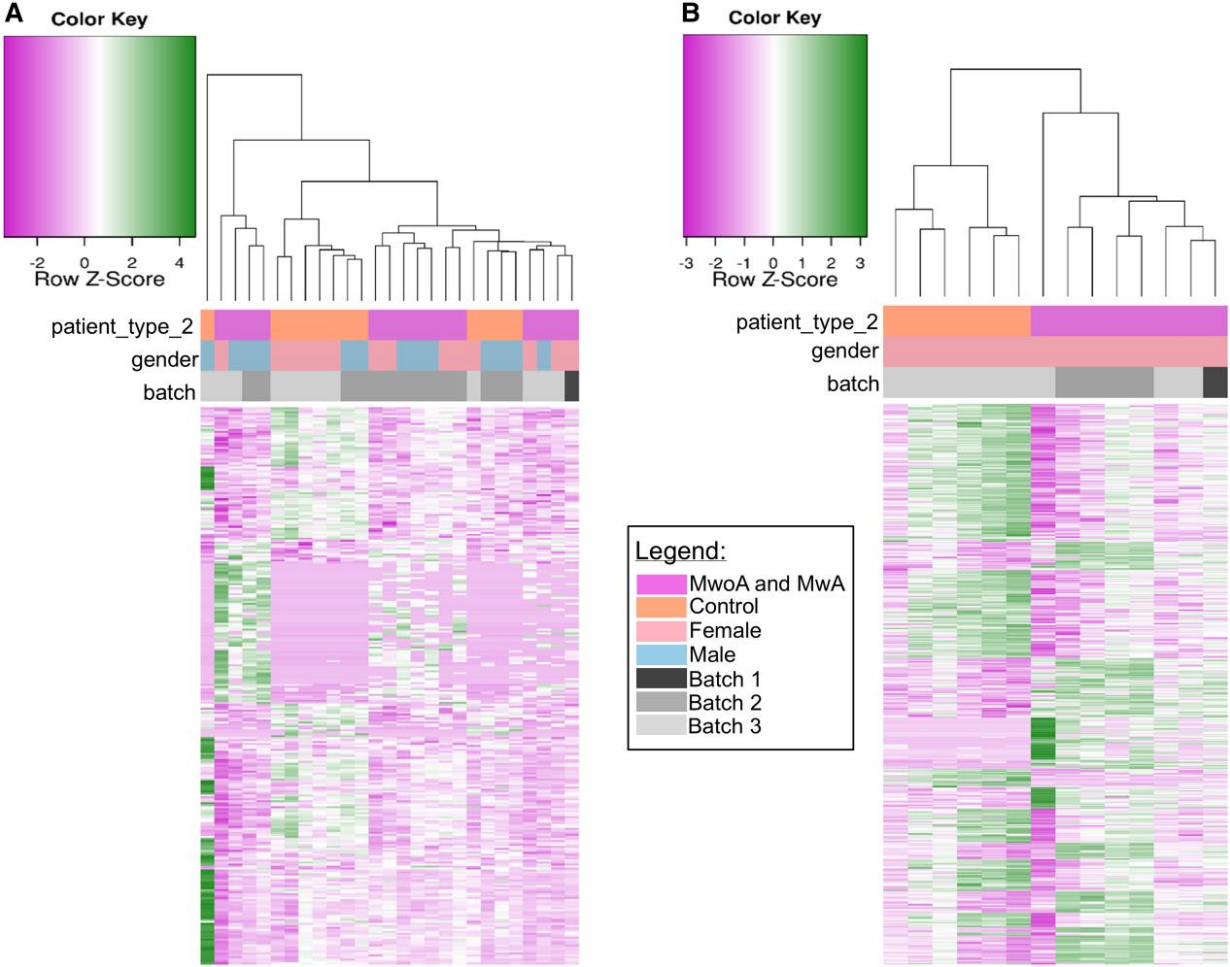
**Heatmap displays significant DEGs in peripheral blood samples**. Migraine patients and controls (*N* = 27) (**A**), female migraine patients and female controls (*N* = 14) (**B**). The values are represented as *z*-scores, a value that came from scaling between samples. Z-score is calculated by: (gene expression value in each sample)−(mean expression across all samples)/standard deviation. Labels at the bottom indicate the sample type, gender and batch effect (sequencing runs performed at different time points) and the dendrogram reveals the results of hierarchical clustering of the genes based on their expression patterns.

**Figure 3:**
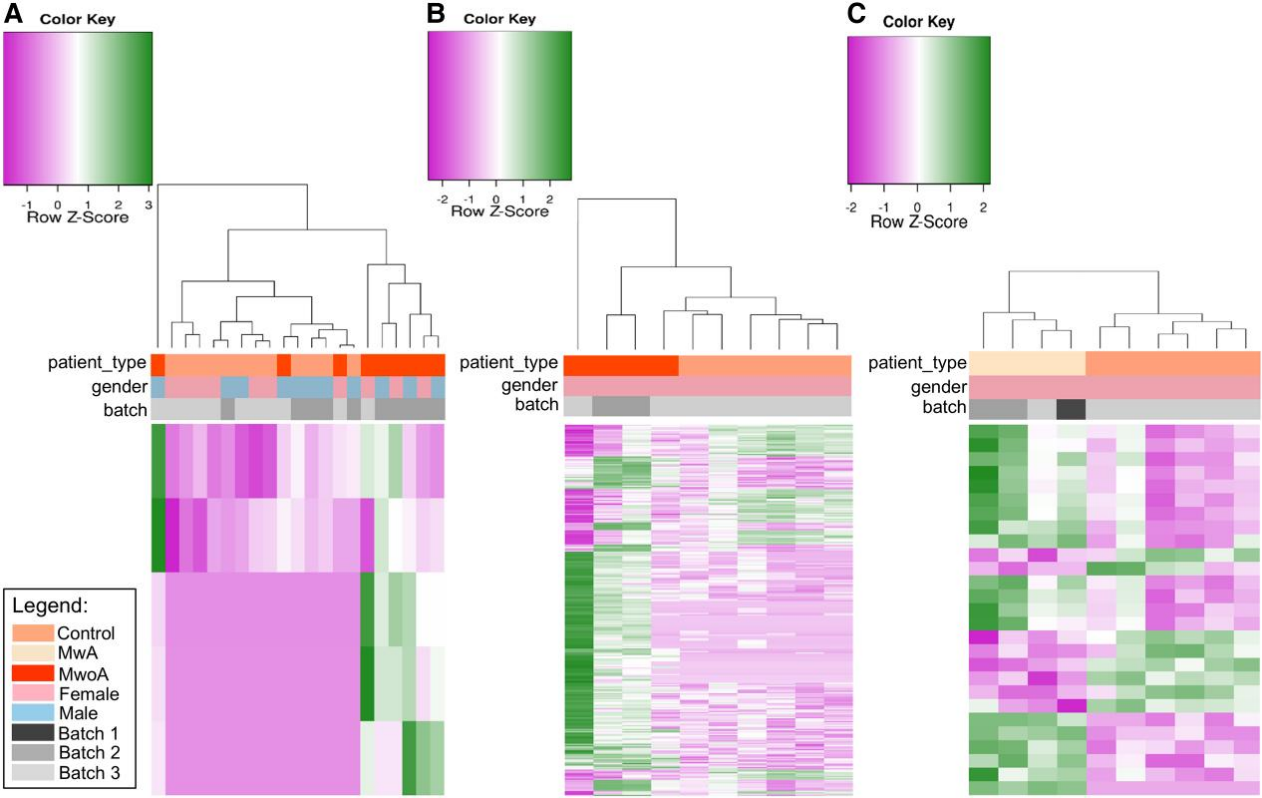
**Heatmap displays significant DEGs in peripheral blood samples comparing migraine patients subgroups to controls**. MwoA patients and controls (*N* = 21) (**A**), female MwoA patients and controls (*N* = 10) (**B**), female MwA patients and controls (*N* = 10) (**C**). The values are represented as *z*-scores, a value that came from scaling between samples. Z-score is calculated by: (gene expression value in each sample)−(mean expression across all samples)/standard deviation. Labels at the bottom indicate the sample type, gender and batch effect (sequencing runs performed at different time points), and the dendrogram reveals the results of hierarchical clustering of the genes based on their expression patterns.

Visualization of DEGs using heatmaps (Figs 2 and 3) revealed distinct clustering patterns that aligned with the original sample groupings: migraine patients versus controls (Fig. 2A), female migraine patients versus female controls (Fig. 2B) MwoA patients versus controls (Fig. 3A), female MwoA patients versus female controls (Fig. 3B) and female MwA patients versus female controls (Fig. 3C).

Our comparative analysis of the combined patient group (MwoA and MwA) versus control group identified 282 DEGs. However, upon segmenting the analysis by gender, a notable observation emerged, the number of DEGs, encompassing both upregulated and downregulated genes, substantially increased within the female group, while the male group exhibited no DEGs (as shown in Supplementary Fig. 1C).

This pattern also held true for the individual analyses involving the MwoA versus controls and MwA versus controls subgroups, mirroring the findings from the overall patient group. Consequently, we can state that these differences in DEGs are exclusive to the female cohort.

Nonetheless, when we conducted a comparison between males with MwA and the male control group, we identified the presence of 5 upregulated genes. It is important to note that for meaningful conclusions regarding the male MwA group, it will be necessary to increase the sample size to draw further conclusions (Supplementary Fig. 1E).

**Figure 4:**
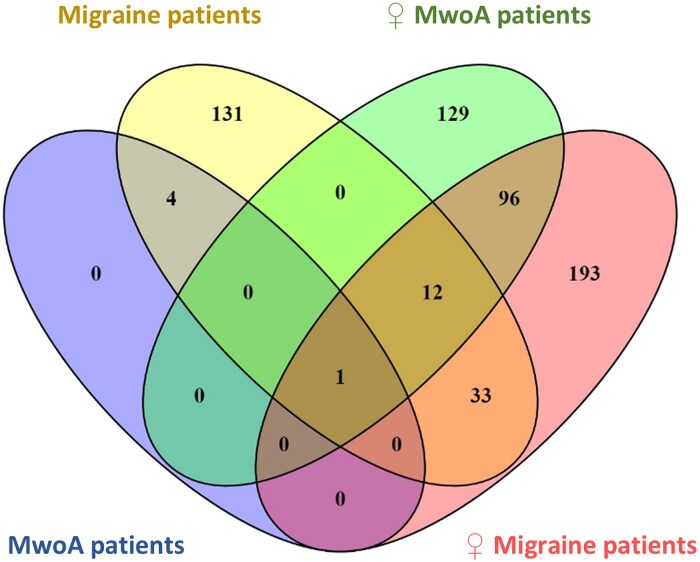
**A Venn diagram illustrating the overlap of mapped DEGs between different groups**. The numbers within each circle indicate the number of DEGs among various comparisons [migraine subgroups versus control (*N* = 27)]. The overlapping numbers denote the shared DEGs across distinct comparisons, while the non-overlapping figures delineate genes specific to each condition: migraine patients, female migraine patients, MwoA patients and female MwoA patients.

Mapped DEGs overlap between comparison pairs (migraine patients versus controls, female migraine patients versus female controls, MwoA patients versus controls and female MwoA patients versus female controls) are presented in Fig. 4.

Upon interpretation of Fig. 4, it becomes evident that in both migraine patients versus controls and female migraine patients versus female controls comparisons, 33 DEGs are shared, whereas each group also displays a substantial number of unique DEGs. This pattern persists in the comparisons involving MwoA patients-Controls and Female MwoA patients-Controls comparisons, with no DEGs in common between these two groups, and 129 DEGs exclusive to the female MwoA patients-Controls group. These observations strongly suggest a notable gender-related discrepancy in gene expression, indicating that the pathophysiological mechanisms of migraine differ between men and women.

In the comparisons between female migraine patients-controls and female MwoA patients-controls, we found that there are many DEGs in common with these two groups. This prompts the hypothesis that the two entities, namely MwoA and MwA, might not differ significantly in terms of migraine pathophysiology. This notion is supported by the absence of DEGs in the MwoA-MwA comparison in this study. However, it will be necessary to increase the sample to be able to draw some conclusions.

### Functional enrichment analysis

Subsequently, we conducted a functional enrichment analysis of the DEGs using GSEA, resulting in the identification of statistically significant affected pathways in migraine patients, female migraine patients and female MwoA patients (supplementary Tables 8–10).

**Figure 5:**
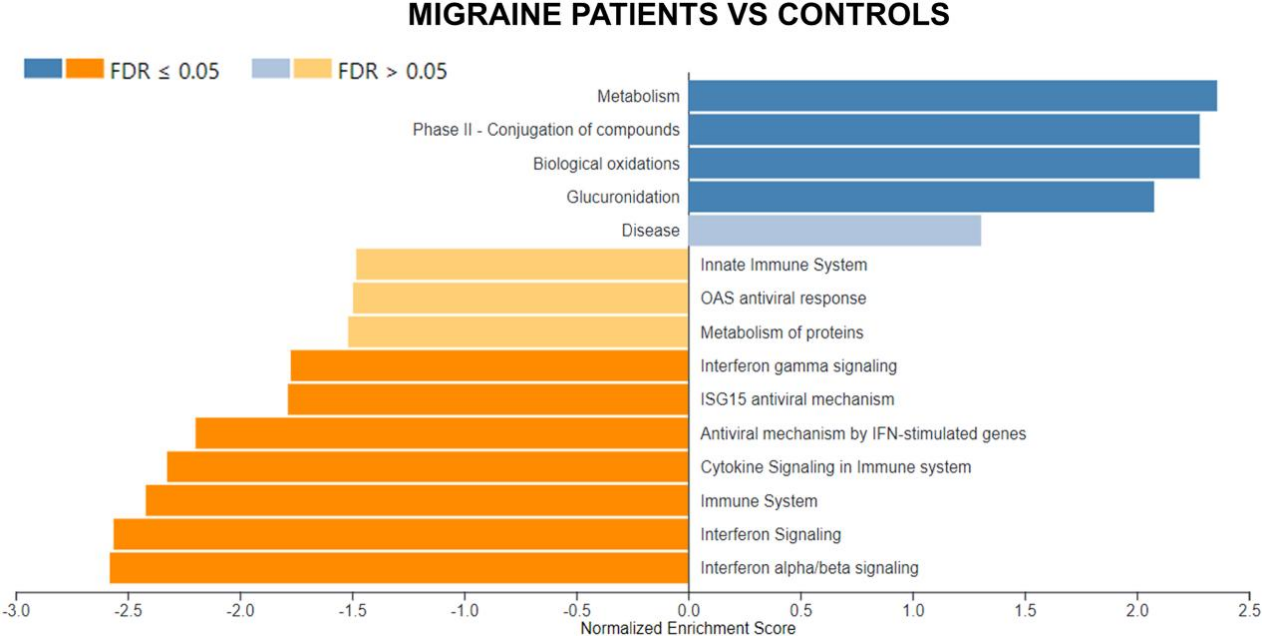
**Bar graph of pathway analysis according to Reactome database using WebGestalt software in the migraine patients group versus healthy controls (*N* = 27).** Only pathways with an FDR < 0.05 were selected as statistically significant. FDR, false discovery rate.

**Figure 6:**
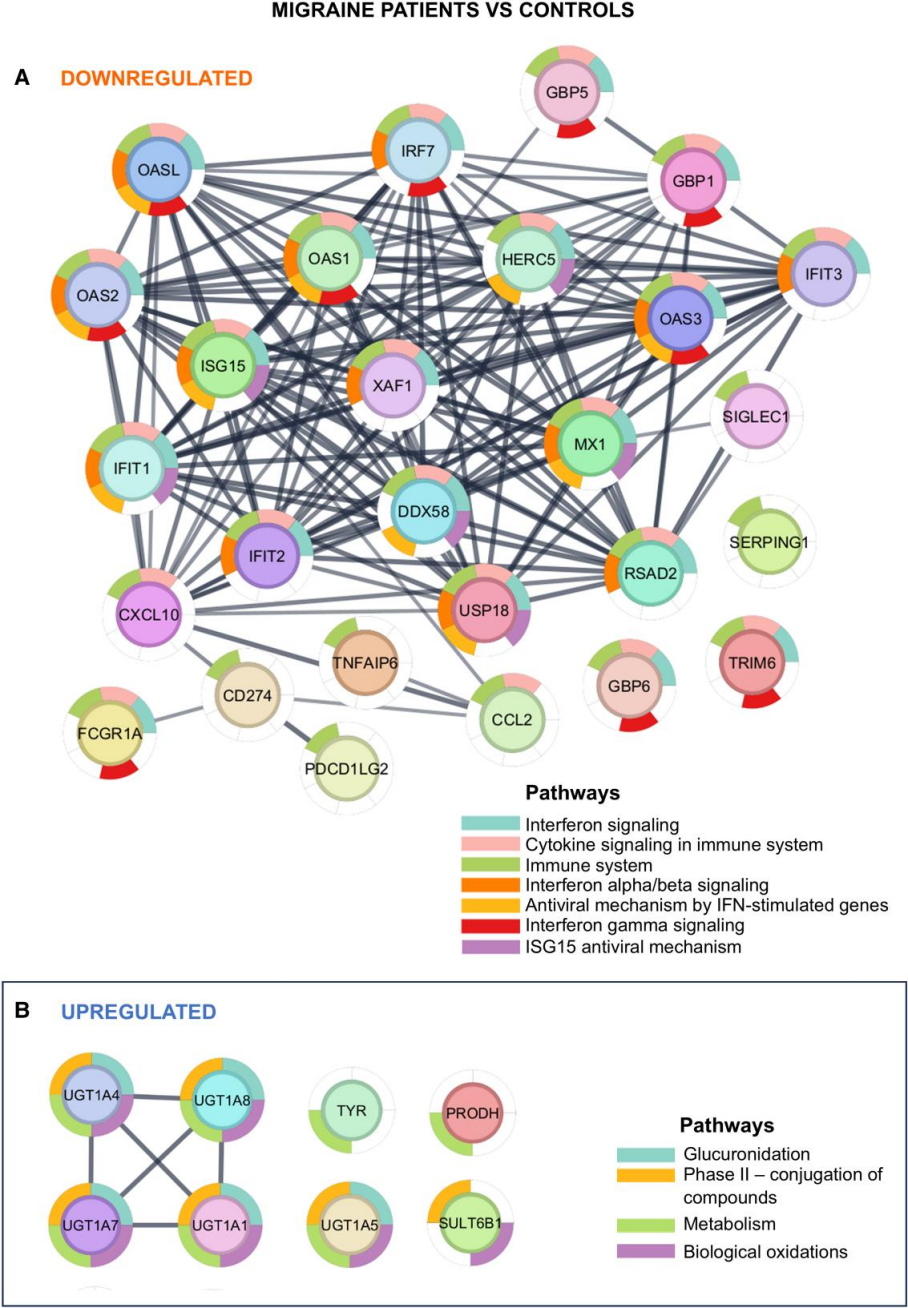
**Functional enrichment analysis of DEGs in the migraine patients group versus healthy controls (*N* = 27)**. DEGs were sorted based on their log_2_FC value resulting in a pre-ranked gene list that was further used in a GSEA. Downregulated genes and associated pathways network (**A**). Upregulated genes and associated pathways network (**B**). Log_2_FC—log 2-fold change.

Analysis of the group of migraine patients and controls highlighted 4 upregulated (‘Metabolism’, ‘Phase II- conjugation of compounds’, ‘Biological oxidations’, ‘Glucuronidation’) and 7 downregulated pathways (‘Interferon gamma signaling’, ‘ISG15 antiviral mechanism’, ‘Immune system’, ‘Cytokine signaling in immune system’, ‘antiviral mechanism by IFN-stimulated genes’, ‘Interferon signaling’, ‘Interferon alpha/beta signaling’) with FDR < 0.5 (Fig. 5). Subsequently, we inferred about the interactions between downregulated (Fig. 6A) and upregulated (Fig. 6B) DEGs and associated pathways in the migraine patients versus controls group. Since the comparison between MwoA patients versus controls only detected 5 DEGs, it was not possible to perform the GSEA in this group.

**Figure 7:**
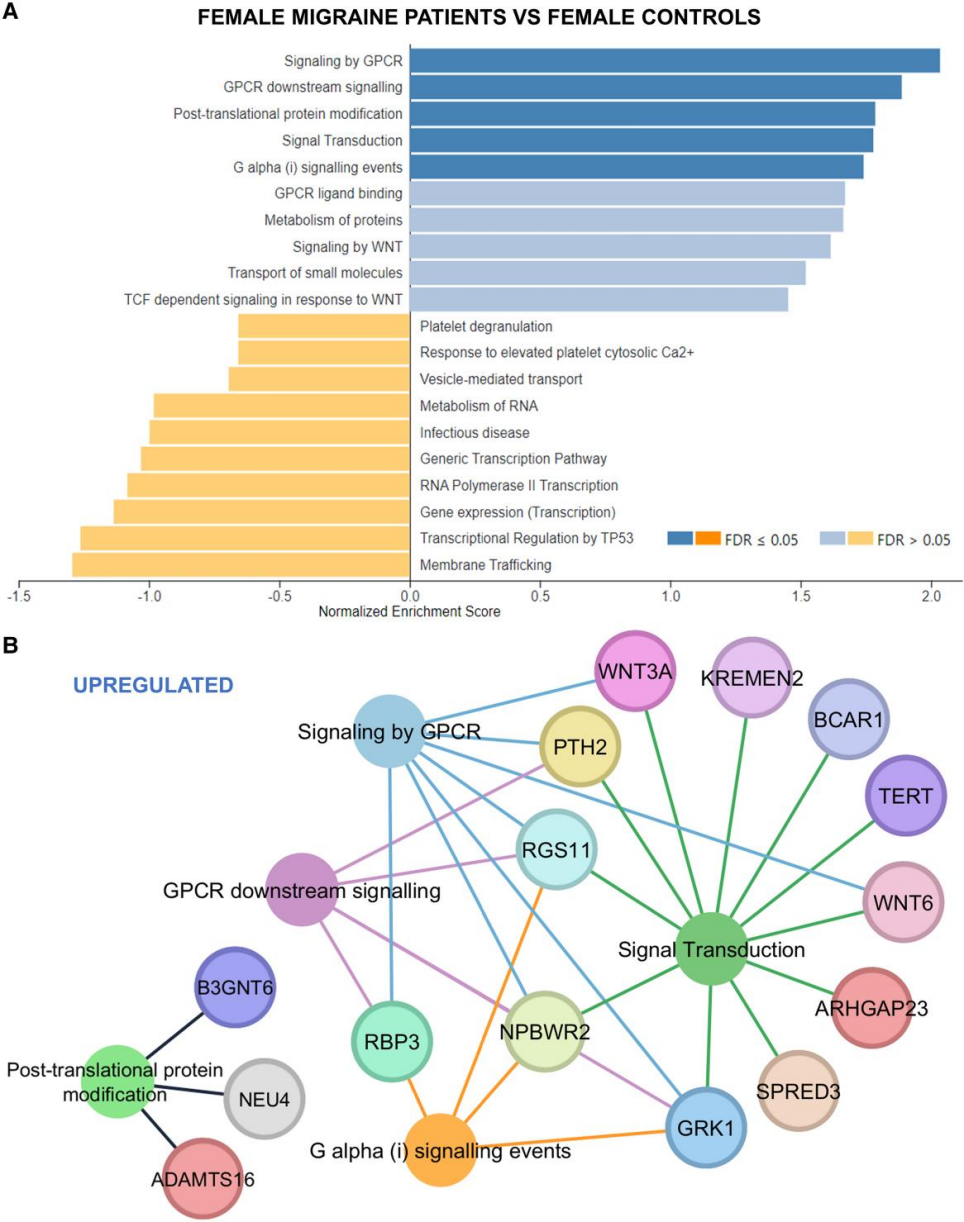
**Functional enrichment analysis of DEGs in the female migraine patients group versus female healthy controls (*N* = 14).** DEGs were sorted based on their log_2_FC value resulting in a pre-ranked gene list that was further used in a GSEA. Only pathways with an FDR < 0.05 were selected as statistically significant. Bar graph of pathway analysis according to Reactome database using WebGestalt software (**A**). Upregulated genes and associated pathways network (**B**). FDR, false discovery rate; log_2_FC, log 2-fold change.

GSEA for female migraine patients versus female controls unveiled 5 significant upregulated pathways, including ‘signaling by GPCR’, ‘GPCR downstream signaling’, ‘Pos-translational protein modification’, ‘signal transduction’, and ‘G alpha (i) signaling events’ pathways (Fig. 7A). No downregulated pathways were found when analyzing this group. Considering the different pathways, we have included a gene set enrichment network in Fig. 7B, corresponding to the significant upregulated genes and associated pathways.

**Figure 8:**
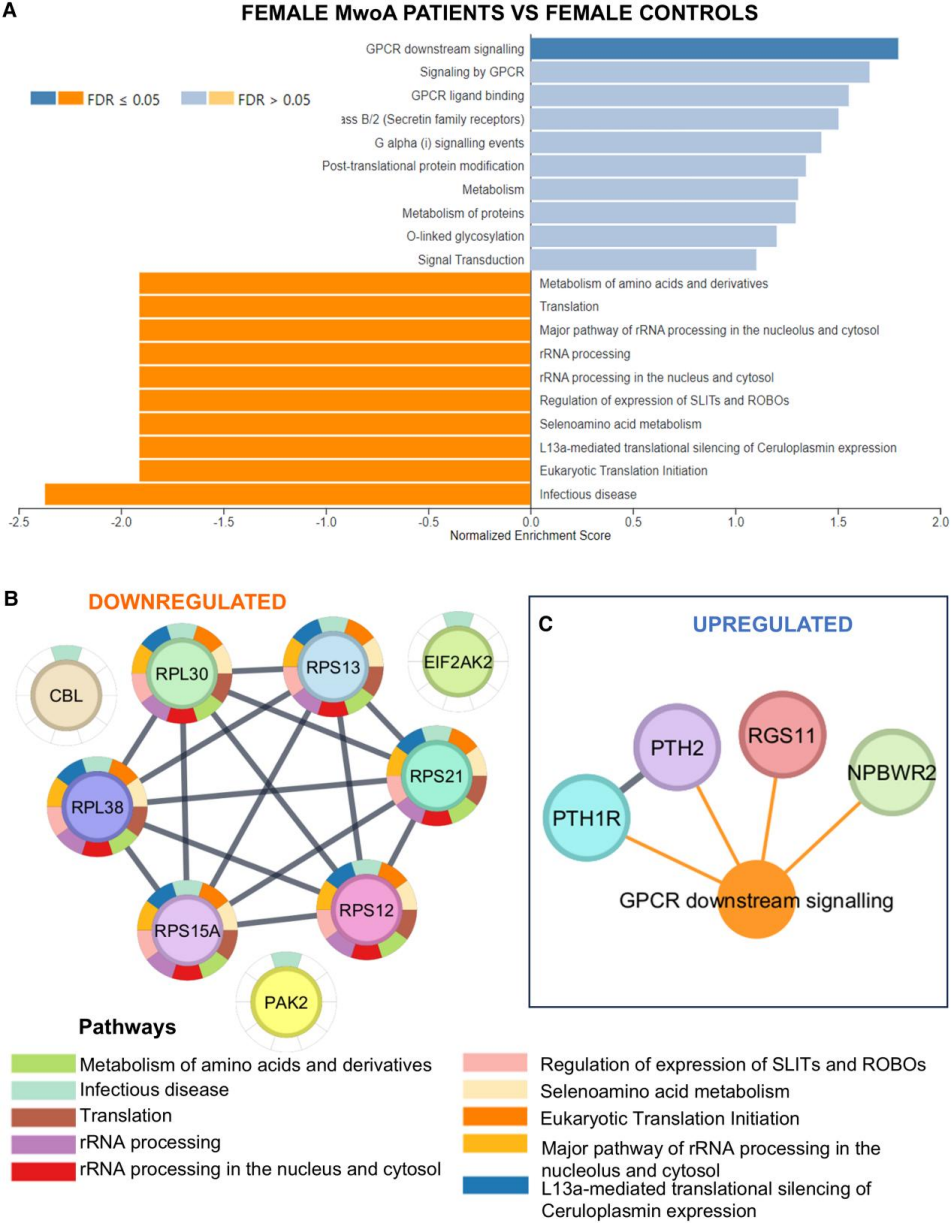
**Functional enrichment analysis of DEGs in the female MwoA patients group versus female healthy controls (*N* = 10).** DEGs were sorted based on their log_2_FC value resulting in a pre-ranked gene list that was further used in a GSEA. Only pathways with an FDR < 0.05 were selected as statistically significant. Bar graph of pathway analysis according to Reactome database using WebGestalt software (**A**). Downregulated genes and associated pathways network (**B**). Upregulated genes and associated pathways network (**C**). FDR, false discovery rate; log_2_FC, log 2-fold change.

Additionally, GSEA for the group of female MwoA patients versus female controls demonstrated that there is one upregulated pathway (‘GPCR downstream signaling’) and 10 downregulated pathways (‘Metabolism of amino acids and derivatives’, ‘Translation’, ‘Major pathway of rRNA processing in the nucleolus and cytosol’, ‘rRNA processing’, ‘rRNA processing in the nucleus and cytosol’, ‘regulation of expression of SLITs and ROBOs’, ‘Selenoamino acid metabolism’, ‘L13a-mediated translational silencing of Ceruloplasmin expression’, ‘Eukaryotic translation Initiation’, and ‘Infectious disease’) with FDR < 0.5 (Fig. 8A). The visual network of genes associated with each pathway is represented in Fig. 8B and C.

**Figure 9:**
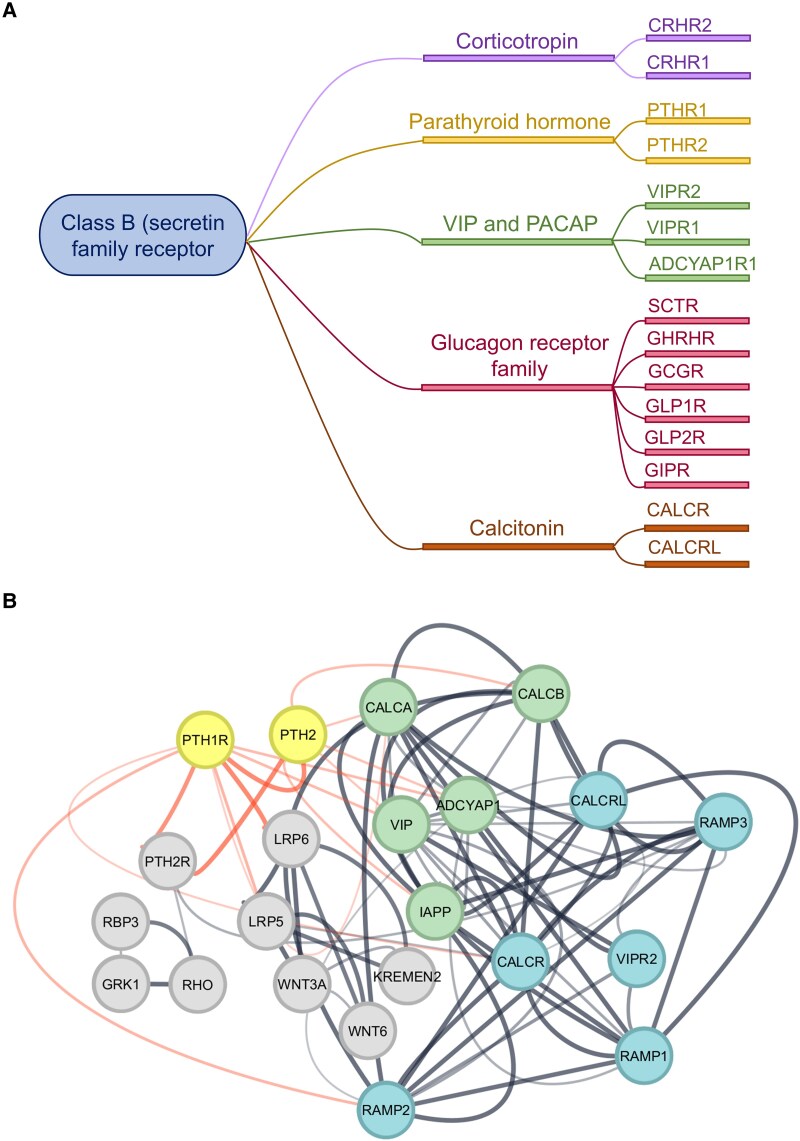
**Class B of GPCRs and the interaction of some interesting proteins with therapeutic targets in migraine**. Proteins belonging to class B of GPCRs (**A**). Protein–protein interaction (PPI) of the proteins found in this study (highlighted in yellow) that interact with each other (red lines); in green are the neuropeptides widely associated with migraine, and in blue are some of the receptors that have been therapeutic targets for migraine (**B**).

Interestingly, the class B (secretin family) of G protein-coupled receptors (GPCRs) has been widely studied in the migraine treatment. In Fig. 9, we can see that the PTH1R, found in GSEA of the MwoA group compared to controls, interacts with other proteins, namely calcitonin gene-related peptide—CGRP (encoded by *CALCA* and *CALCB* genes) and pituitary adenylate cyclase-activating peptide—PACAP (*ADCYAP1* gene), two therapeutic targets in vogue in the migraine development.

In summary, our analysis of migraine patients versus controls identified 282 DEGs, with a marked increase in DEGs among females and none observed in males. Significant overlap in DEGs between female migraine and female MwoA patients suggests minimal pathophysiological differences between MwA and MwoA.

Additionally, PTH1R and PTH2, found in GSEA of the female migraine patients, emerged as significant, interacting with key therapeutic targets in migraine research like CGRP and PACAP.

## Discussion

We analyzed gene expression profiles of whole blood from Portuguese migraine patients (MwoA and MwA) and healthy controls using RNA-Seq.

Comparative analysis revealed disparities in gene expression profiles between migraine patients and healthy controls (Figs 1A and 2A), with immune-related pathways downregulated (Fig. 6A) and metabolism-related pathways upregulated (Fig. 6B).

These findings align with previous studies highlighting altered gene expression levels in individuals with migraine, such as in Gerring *et al*.^14^, in which they compared migraine patients with controls and identified 53 DEGs, and highlighted altered immune-inflammatory pathways, specifically changes in interferon and cytokine signalling.^15^

Other studies on peripheral blood mononuclear cells (PBMCs) of migraine patients underscored the role of inflammatory pathways and cytokines, alongside mitochondrial dysfunction, in migraine susceptibility. These studies reported an upregulation of several cytokines and COX-2, indicating a systemic shift in immune functions. These findings align with prior reports of increased plasma levels of proinflammatory cytokines such as IL-1, IL-6, TNFα, IL-8, CCL3, CCL5 and C-reactive protein (CRP) in migraine.^16^

Similarly, animal models of chronic migraine revealed immune response and glutamate signalling genes, along with changes in oxidative stress regulation.^17^ Kogelman *et al*.^18^ compared transcriptome expressions in trigeminal ganglia and dorsal root ganglia of naïve rats, identifying DEGs, including some previously associated with migraine like *CACNA1A* and *ATP1A2.*

Some previous studies proposed potential biomarkers for MwA patients, such as alpha-fodrin,^19^ and identified differences in *NMNAT2* and *RETN* genes in the peripheral blood of MwA patients compared to controls.^20^ In our study, no significant DEGs were found between MwA patients and controls (Fig. 1E). Limited DEGs in female MwA patients also restricted the scope of GSEA, reflecting challenges in replicating prior findings and proposing biomarkers.

Our findings reinforce the role of immune-inflammatory pathways in migraine (Fig. 6A). Neurogenic inflammation, triggered by neuropeptides, like CGRP and PACAP, prompts mast cell degranulation, releasing histamine, serotonin and pro-inflammatory cytokines (e.g. TNF-α, IL-1, IL-6),^21^ which are mechanisms involved in the pathophysiology of migraine.^22^

However, despite evidence of elevated cytokines in migraine patients, our results suggest potential compensatory immune pathway downregulation, possibly mitigating excessive inflammation and weakening immune responses.^23^ Genetic predisposition, such as cytokine-related gene polymorphisms, might further contribute to immune modulation in migraine patients.^22^

In this study, upregulated metabolic pathways were also observed (Fig. 6B), consistent with the hypothesis that migraine involve energy deficits and mitochondrial dysfunction.^24^ Reduced ATP concentrations and impaired oxidative metabolism have been linked to migraine,^25^ with epigenetic mechanisms, like mitochondrial methylation, offering insights into cortical spreading depression (CSD) susceptibility.^26^ Addressing metabolic deficiencies may present therapeutic opportunities to ameliorate mitochondrial energy depletion and mitigate migraine severity.^24^

Gender-specific analyses in our study highlighted DEGs in female migraine patients (both MwoA and MwA), suggesting upregulation of GPCR signalling pathways (Figs 7B and 8C). Additionally, the comparison between female MwoA patients and female healthy controls suggest that pathways linked to ribosome biogenesis and translation, as well as pathways related to infectious disease are downregulated (Fig. 8B).

GPCRs, critical in disease regulation, are promising drug targets.^27^ Regulatory mechanisms for GPCRs primarily involve G protein-coupled receptor kinases, arrestins and regulator of G protein-signalling proteins. Regulator of G protein-signalling proteins often function as GTPase-activating proteins for Gα subunits, controlling the kinetics and magnitude of GPCR signalling, thereby regulating the downstream effectors, and signalling pathways.^28^

GPCRs are commonly classified using the A-F system, primarily based on their amino acid sequences and functional similarities.^29^ The Class B (Secretin Receptor Family) consists of 15 receptors for peptide hormones like glucagon receptors, corticotropin, vasoactive intestinal peptide and PACAP, parathyroid hormone (PTH) and calcitonin (CT) (Fig. 9A). These receptors primarily couple to stimulatory G proteins, leading to increased intracellular cAMP levels.^30^

Among these receptors, the parathyroid hormone receptors (PTH1R and PTH2R) stand out, primarily interacting through coupling with the stimulatory G protein of adenylyl cyclase (Gs). PTH2R emerges as a pivotal mediator in nociception and recent investigations have elucidated its role in regulating calcium transport and impacting keratinocyte differentiation.^31^

In our study, it appears that the *PTH1R* gene is upregulated in female MwoA patients compared to female controls (Fig. 8C). PTHrP (parathyroid hormone-related protein) triggers the activation of PTH1R, subsequently leading to downstream activation of protein kinases C and/or A (PKA).^32^ CGRP binds to receptors on endothelial cells, initiating a PKA-mediated sequence that activates endothelial nitric oxide synthase, thereby enhancing the production of nitric oxide. Subsequently, nitric oxide spreads to neighboring vascular smooth muscles, prompting vasodilation.^33^ Our result can reveal that the increase in PTH1R can facilitate the cascade that leads to vasodilation and an increase in migraine attacks.

Some studies identified the involvement of two endogenous neuropeptides in the pathophysiology of migraine: CGRP and PACAP.^34^ CGRP manifests in two human isoforms: α-CGRP and β-CGRP, encoded by the *CALCA* and *CALCB* genes, respectively.^35^ On the other hand, PACAP is encoded by the *ADCYAP1* gene and exhibits two functional isoforms: PACAP38 (representing approximately 90% of total PACAP) and PACAP27. Both PACAP isoforms acts on four receptors, demonstrating similar affinity and functionality.^34^ CGRP and PACAP have been shown to induce attacks, though PACAP uniquely triggers premonitory-like symptoms.

The significance of CGRP in migraine has been extensively established, particularly evident in the success of eight FDA-approved CGRP-based therapeutics (four anti-CGRP monoclonal antibodies—‘mAbs’ and four CGRP receptor small-molecule inhibitors—‘Gepants’). Three of the mAbs target CGRP directly, while one binds to the canonical CGRP receptor. In contrast, all gepants function as antagonists of the canonical CGRP receptor.^36^

However, these agents significantly benefit only about 40–60% of migraine patients, suggesting the involvement of additional factors beyond CGRP in migraine pathophysiology.^37^ A phase 2 trial for Lu AG09222, the first PACAP-targeting monoclonal antibody directed against the PACAP ligand, highlights its potential as a novel preventive treatment for migraine. This development offers new hope for patients unresponsive to existing therapies.^38^

PAC1 receptor exhibits high homology with receptors like the vasoactive intestinal peptide receptor, secretin receptor, GLP-1 receptor, PTH-PTHrP receptor and CT receptor.^39^ This similarity raises doubts about which other receptors might be involved in migraine. Hence, in the quest to identify the relevant receptors for CGRP and PACAP in migraine, it is important to note that other members of the GPCR peptide family can also be involved in migraine susceptibility.^37^

Collectively, these findings offer new perspectives on class B GPCRs such as PTH1R and PTH2R, emerging as robust and promising candidates for novel clinical targets in conditions like migraine and other painful disorders. Importantly, these receptors establish interactions with the most studied neuropeptides in migraine (CGRP and PACAP) as we can see in Fig. 9B.

The ability to identify changes in gene expression is highly dependent on sample size, which may be a limitation of our study. Using the RNASeqPower software (v1.1.0),^40^ we estimated a power of 84% to detect DEGs with a fold change of 1.5 for genes exhibiting an average coefficient of variation of 0.3. However, for genes with a coefficient of variation of 0.5, the power to detect the same fold change dropped to 49%. Based on these findings, we conclude that our study had sufficient power to detect expression changes in genes with low to moderate variability. Conversely, it lacked the statistical power necessary to reliably detect changes in genes with high variability across samples.

Replicating other studies can be controversial, as genetic background of the populations studied is different and also, the design of the study, ascertainment of the samples and methodologies to analyze the results may be different.

Additionally, our study, as mentioned previously, has focused on peripheral blood as the source of investigation. This approach may have its limitations, as migraine is predominantly viewed as a brain disorder, with vascular system involvement primarily at the blood vessel level, such as vasodilation.^14^ Since migraine has a very large clinical heterogeneity, it has been extremely difficult to find objective clinical biomarkers. Peripheral blood emerges as an attractive candidate for genomic studies of migraine due to its ease of acquisition and the ability to collect multiple samples with minimal discomfort to the patient. Existing evidence indicates that most expressed genes in brain tissue are also found in blood, with tissue-specific expression driven by a relatively small number of genes.^14^ The development of a blood gene expression signature specific to migraine holds the potential to significantly enhance the clinical management of patients with this condition.

## Conclusions

Migraine is a complex disorder, requiring different disciplines and foster multi-actor engagement in the research process. Employing innovative methodologies, such as RNA-seq, to establish validated genomic profile diagnostics within a well-characterized clinical cohort holds the promise of assisting clinicians in managing migraine patients more effectively.

Given the notable proportion of non-responders to CGRP(-receptor) blocking agents, it is imperative to explore alternative therapeutic avenues.

In summary, the findings of this study present fresh insights into class B GPCRs like PTH1R and PTH2R, standing out as sturdy and promising targets for innovative therapeutic in conditions such as migraine and various painful disorders.

## Supplementary Material

fcaf142_Supplementary_Data

## Data Availability

All data generated or analysed during this study are included in this published article [and its supplementary information files].
