# Supplementary material for: Decoding migraine disorders: parathyroid hormone-related peptide receptors as key genetic drivers
Source: Brain Commun. 2025 Apr 28;7(2):fcaf142. doi: 10.1093/braincomms/fcaf142 (PMC12034459; doi:10.1093/braincomms/fcaf142)
Supplement: fcaf142_Supplementary_Data [file fcaf142_supplementary_data.pdf]

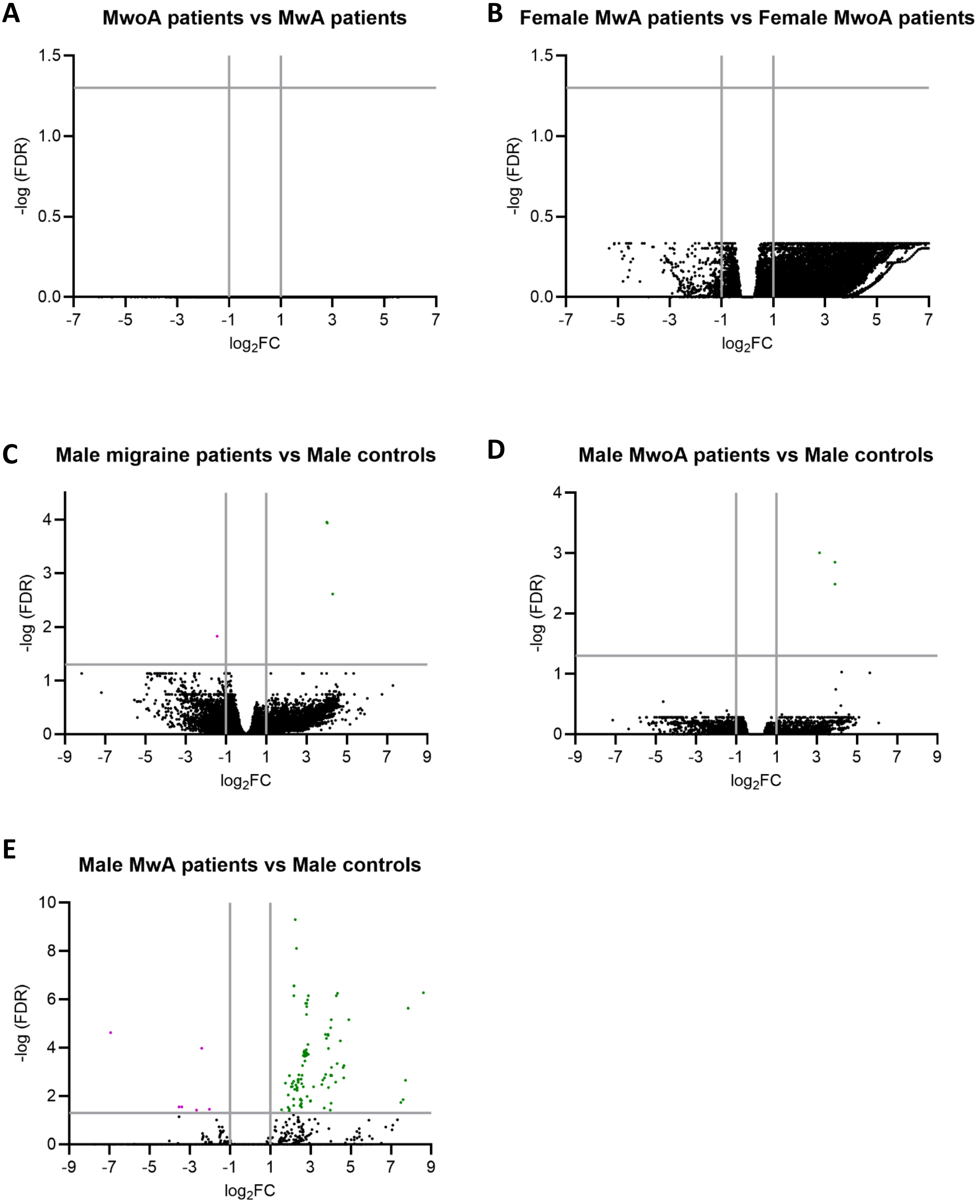

Supplementary Figure 1: Volcano plots of DEGs between MwoA patients and MwA patients (**A**), female MwA patients and female MwoA patients (**B**), male migraine patients and male controls (**C**), male MwoA patients and male controls (**D**) and between male MwA patients and male controls (**E**). Volcano plot displaying individual genes, with a significance threshold of $|\log_2 FC| \geq 1$ and adjusted p-value < 0.05. Magenta and green dots indicate downregulated and upregulated DEGs, respectively.

Supplementary table 1: Differentially expressed genes (migraine patients vs. controls). Log_2FC – Log 2-fold change; logCPM – log counts per million; LR – likelihood ratio; FDR – False discovery rate

Transcript	Transcript stable ID	chr	start	end	Gene name	Gene type	Canonical	MANE Select	log_2FC	logCPM	LR	P-Value	FDR
**ENST00000228928**	ENSG00000111331	12	112938473	112973251	OAS3	protein_coding	Yes	NM_006187.4	-2.7111	3.7528	29.1399	6.73E-08	0.0004
**ENST00000278407**	ENSG00000149131	11	57597684	57614848	SERPING1	protein_coding	Yes	NM_000062.3	-3.2317	-0.3856	30.0450	4.22E-08	0.0004
**ENST00000394662**	ENSG00000225492	1	89410318	89426243	GBP1P1	transcribed_unprocessed_pseudogene	Yes	-	-3.9158	0.0054	28.8531	7.81E-08	0.0004
**ENST00000440769**	ENSG00000234292	5	91280096	91281142	-	lncRNA	Yes	-	-1.2805	-0.6780	27.8821	1.29E-07	0.0005
**ENST00000382040**	ENSG00000134321	2	6877776	6898239	RSAD2	protein_coding	Yes	NM_080657.5	-3.8359	5.5697	27.5983	1.49E-07	0.0005
**ENST00000265598**	ENSG00000078081	3	183122214	183162734	LAMP3	protein_coding	Yes	NM_014398.4	-3.6882	-0.2815	27.5361	1.54E-07	0.0005
**ENST00000306602**	ENSG00000169245	4	76021117	76023497	CXCL10	protein_coding	Yes	NM_001565.4	-3.6550	0.4426	26.6681	2.42E-07	0.0007
**ENST00000513638**	ENSG00000290525	1	89407678	89424934	GBP1P1	lncRNA	Yes	-	-3.8885	-0.8397	26.1587	3.14E-07	0.0008
**ENST00000370751**	ENSG00000137959	1	78620447	78646145	IFI44L	protein_coding	Yes	NM_006820.4	-3.3171	4.5726	25.5323	4.35E-07	0.0010
**ENST00000258381**	ENSG00000135899	2	230165185	230219984	SP110	protein_coding	Yes	NM_080424.4	-1.0560	3.4018	24.3998	7.83E-07	0.0014
**ENST00000652439**	ENSG00000290727	2	73641082	73697509	ALMS1P1	lncRNA	Yes	-	-2.9933	-1.5123	24.4089	7.79E-07	0.0014
**ENST00000433113**	ENSG00000240527	10	95833507	95873758	-	lncRNA	Yes	-	-1.1951	-1.5197	23.7829	1.08E-06	0.0018
**ENST00000215794**	ENSG00000184979	22	18150169	18177397	USP18	protein_coding	Yes	NM_017414.4	-3.1833	1.2401	23.6537	1.15E-06	0.0019
**ENST00000233057**	ENSG00000055332	2	37099209	37156980	EIF2AK2	protein_coding	Yes	NM_001135651.3	-1.4056	4.0975	23.1799	1.48E-06	0.0021
**ENST00000584605**	ENSG00000266728	17	27623363	27640777	-	protein_coding	Yes	-	-1.1445	-0.4007	23.1319	1.51E-06	0.0021
**ENST00000371804**	ENSG00000185745	10	89392622	89406487	IFIT1	protein_coding	Yes	NM_001548.5	-3.0510	5.6498	23.1113	1.53E-06	0.0021
**ENST00000264350**	ENSG00000138646	4	88457118	88506163	HERC5	protein_coding	Yes	NM_016323.4	-2.6450	4.3581	22.6218	1.97E-06	0.0025
**ENST00000649529**	ENSG00000187608	1	1013496	1014540	ISG15	protein_coding	Yes	NM_005101.4	-3.0875	4.5657	22.3362	2.29E-06	0.0028
**ENST00000344754**	ENSG00000088827	20	3686969	3712600	SIGLEC1	protein_coding	Yes	NM_023068.4	-2.6839	0.3252	22.1928	2.47E-06	0.0029
**ENST00000395473**	ENSG00000168961	17	27631187	27649560	LGALS9	protein_coding	Yes	NM_009587.3	-1.1296	0.6851	21.9384	2.82E-06	0.0032

**ENST00000580979**	ENSG00000264456	17	30971651	30973312	-	lncRNA	Yes	-	-1.2068	-2.6522	21.6305	3.31E-06	0.0035
**ENST00000493687**	ENSG00000242256	9	112242549	112242839	RN7SL57P	misc_RNA	Yes	-	-1.7494	-3.6268	21.6109	3.34E-06	0.0036
**ENST00000515156**	ENSG00000250138	5	69631962	69636399	-	unprocessed_pseudogene	Yes	-	-1.7674	-1.4149	21.4385	3.65E-06	0.0037
**ENST00000696174**	ENSG00000188897	16	11372014	11527247	-	protein_coding	Yes	NM_001370704.1	-1.1063	2.8201	21.1652	4.21E-06	0.0040
**ENST00000428767**	ENSG00000163016	2	73671029	73685085	ALMS1P1	transcribed_unprocessed_pseudogene	Yes	-	-2.9664	-2.6659	20.9397	4.74E-06	0.0043
**ENST00000634862**	ENSG00000290937	3	198167738	198222513	-	lncRNA	Yes	-	-1.1384	1.2190	20.7411	5.26E-06	0.0046
**ENST00000401216**	ENSG00000216035	10	29602263	29602346	MIR938	miRNA	Yes	-	-2.1266	-4.5377	20.6720	5.45E-06	0.0047
**ENST00000381577**	ENSG00000120217	9	5450541	5470554	CD274	protein_coding	Yes	NM_014143.4	-2.2562	2.2694	20.5867	5.70E-06	0.0048
**ENST00000371930**	ENSG00000152766	10	88819895	88851844	ANKRD22	protein_coding	Yes	NM_144590.3	-2.5352	0.9572	20.3754	6.36E-06	0.0050
**ENST00000578539**	ENSG00000264853	17	75370946	75373736	-	lncRNA	Yes	-	-1.2227	0.2788	20.1003	7.35E-06	0.0053
**ENST00000624318**	ENSG00000279476	16	81517823	81520617	-	TEC	Yes	-	-1.3059	-0.2770	20.1083	7.32E-06	0.0053
**ENST00000392619**	ENSG00000186407	17	74609884	74623738	CD300E	protein_coding	Yes	NM_181449.3	-1.0329	2.8121	20.0490	7.55E-06	0.0053
**ENST00000429111**	ENSG00000224083	9	5096665	5098193	MTCO1P11	unprocessed_pseudogene	Yes	-	-1.5432	-2.7934	20.0165	7.68E-06	0.0054
**ENST00000623625**	ENSG00000279349	3	112525547	112528142	-	TEC	Yes	-	-1.0599	-1.4145	19.8362	8.44E-06	0.0056
**ENST00000268058**	ENSG00000140464	15	73994715	74047827	PML	protein_coding	Yes	NM_033238.3	-1.1471	0.1350	19.5713	9.69E-06	0.0058
**ENST00000398598**	ENSG00000157601	21	41426238	41459212	MX1	protein_coding	Yes	NM_002462.5	-2.0981	4.5725	19.4632	1.03E-05	0.0058
**ENST00000547105**	ENSG00000258316	12	62234389	62235515	KLF17P1	processed_pseudogene	Yes	-	-1.2674	-2.7564	19.2878	1.12E-05	0.0058
**ENST00000581811**	ENSG00000274963	1	150568970	150569269	RN7SL600P	misc_RNA	Yes	-	-1.2781	-0.7303	19.2724	1.13E-05	0.0058
**ENST00000392583**	ENSG00000111335	12	112978518	113011723	OAS2	protein_coding	Yes	NM_002535.3	-1.8796	4.0722	19.2401	1.15E-05	0.0059
**ENST00000638172**	ENSG00000255221	11	105092485	105101414	CARD17	transcribed_unprocessed_pseudogene	Yes	-	-2.2715	-0.3230	19.0269	1.29E-05	0.0062
**ENST00000373450**	ENSG00000242366	2	233617632	233773300	UGT1A8	protein_coding	Yes	NM_019076.5	4.9076	-5.4267	18.9746	1.32E-05	0.0064
**ENST00000371818**	ENSG00000119917	10	89327996	89340968	IFIT3	protein_coding	Yes	NM_001549.6	-2.3643	6.2316	18.8861	1.39E-05	0.0065
**ENST00000580341**	ENSG00000277452	1	150566561	150566860	RN7SL473P	misc_RNA	Yes	-	-1.3804	-1.1213	18.8300	1.43E-05	0.0065
**ENST00000602669**	ENSG00000269940	14	103694559	103695170	-	lncRNA	Yes	-	-1.2022	-1.6669	18.8069	1.45E-05	0.0066

**ENST00000582970**	ENSG00000173821	17	80260851	80398794	RNF213	protein_coding	Yes	NM_001256071.3	-1.1003	7.5623	18.6945	1.53E-05	0.0068
**ENST00000215838**	ENSG00000185339	22	30607173	30627271	TCN2	protein_coding	Yes	NM_000355.4	-1.4475	-1.7885	18.5975	1.61E-05	0.0069
**ENST00000687452**	ENSG00000259366	8	29055928	29057395	-	lncRNA	Yes	-	-1.5163	-1.3505	18.5956	1.62E-05	0.0069
**ENST00000638998**	ENSG00000290797	11	105092468	105101431	-	lncRNA	Yes	-	-2.2037	-1.1569	18.5404	1.66E-05	0.0069
**ENST00000504490**	ENSG00000251441	4	112356134	112359819	RTEL1P1	transcribed_processed_pseudogene	Yes	-	-1.1500	-1.2816	18.5089	1.69E-05	0.0069
**ENST00000202917**	ENSG00000089127	12	112906961	112919903	OAS1	protein_coding	Yes	NM_016816.4	-2.2273	3.7243	18.3164	1.87E-05	0.0070
**ENST00000518552**	ENSG00000254325	8	55893594	55895739	-	lncRNA	Yes	-	-1.3574	-0.5932	18.2364	1.95E-05	0.0071
**ENST00000683103**	ENSG00000168026	3	39107679	39138900	TTC21A	protein_coding	Yes	NM_001366900.1	-1.3997	-0.8697	18.0301	2.17E-05	0.0074
**ENST00000361157**	ENSG00000126709	1	27666063	27672192	IFI6	protein_coding	Yes	NM_002038.4	-2.1784	3.8090	17.9978	2.21E-05	0.0075
**ENST00000370747**	ENSG00000137965	1	78649830	78664073	IFI44	protein_coding	Yes	NM_006417.5	-2.2180	4.1000	17.9641	2.25E-05	0.0075
**ENST00000409140**	ENSG00000196141	2	200306692	200482264	SPATS2L	protein_coding	Yes	NM_001100423.2	-2.1673	0.9578	17.9202	2.30E-05	0.0076
**ENST00000656637**	ENSG00000228358	4	117371529	117412712	LINC02263	lncRNA	Yes	-	4.0355	-5.7103	17.7035	2.58E-05	0.0080
**ENST00000439539**	ENSG00000240288	3	10285753	10293449	GHRLOS	lncRNA	Yes	-	-1.0356	-0.4799	17.6821	2.61E-05	0.0081
**ENST00000467148**	ENSG00000130589	20	63558085	63574239	HELZ2	protein_coding	Yes	NM_001037335.2	-1.2060	3.3793	17.6588	2.64E-05	0.0081
**ENST00000292494**	ENSG00000160932	8	143018528	143022409	LY6E	protein_coding	Yes	NM_002346.3	-1.6999	0.7490	17.6001	2.73E-05	0.0082
**ENST00000340181**	ENSG00000010030	6	36366193	36387669	ETV7	protein_coding	Yes	NM_016135.4	-2.3503	-0.1532	17.5878	2.74E-05	0.0083
**ENST00000257895**	ENSG00000135437	12	55720392	55724705	RDH5	protein_coding	Yes	NM_002905.5	-1.0455	-2.4052	17.4951	2.88E-05	0.0085
**ENST00000390295**	ENSG00000211649	22	22369613	22370087	IGLV7-46	IG_V_gene	Yes	-	2.6031	-1.9015	17.3365	3.13E-05	0.0089
**ENST00000305208**	ENSG00000241635	2	233760269	233773300	UGT1A1	protein_coding	Yes	NM_000463.3	4.6918	-5.5550	17.2384	3.30E-05	0.0091
**ENST00000615155**	ENSG00000236438	3	198180754	198190365	FAM157A	transcribed_unprocessed_pseudogene	Yes	-	-1.0715	-0.7663	17.1057	3.54E-05	0.0096
**ENST00000427236**	ENSG00000238015	1	78666271	78666695	-	processed_pseudogene	Yes	-	-2.7872	-3.3243	17.0873	3.57E-05	0.0097
**ENST00000507317**	ENSG00000251215	5	39169209	39170335	GOLGA5P1	processed_pseudogene	Yes	-	-1.0728	0.0469	16.9856	3.77E-05	0.0100
**ENST00000581261**	ENSG00000284542	17	56891269	56891355	MIR3614	miRNA	Yes	-	-1.1623	-1.8486	16.9780	3.78E-05	0.0100

**ENST00000442435**	ENSG00000237781	1	150548561	150557724	ADAMTSL4-AS2	lncRNA	Yes	-	-1.1451	-0.0109	16.9498	3.84E-05	0.0101
**ENST00000575034**	ENSG00000263069	17	80351827	80415118	RNF213-AS1	lncRNA	Yes	-	-1.1224	-0.3051	16.9149	3.91E-05	0.0102
**ENST00000369384**	ENSG00000291135	1	121087297	121097161	FCGR1BP	lncRNA	Yes	-	-1.3189	1.4319	16.8970	3.95E-05	0.0102
**ENST00000394620**	ENSG00000108700	17	34319434	34321402	CCL8	protein_coding	Yes	NM_005623.3	-4.0007	-3.1168	16.8893	3.96E-05	0.0103
**ENST00000624849**	ENSG00000279320	3	146504569	146506560	-	TEC	Yes	-	-1.4827	-0.8643	16.7749	4.21E-05	0.0107
**ENST00000295934**	ENSG00000163666	3	57197837	57199978	HESX1	protein_coding	Yes	NM_003865.3	-3.4590	-3.0957	16.7203	4.33E-05	0.0108
**ENST00000697334**	ENSG00000198019	1	121087394	121096152	FCGR1BP	transcribed_unprocessed_pseudogene	Yes	-	-1.3136	1.0909	16.6799	4.42E-05	0.0108
**ENST00000370456**	ENSG00000183347	1	89364058	89388160	GBP6	protein_coding	Yes	NM_198460.3	-1.7290	-0.8742	16.6577	4.48E-05	0.0109
**ENST00000436794**	ENSG00000214124	X	24513449	24513687	SNRPEP9	processed_pseudogene	Yes	-	-1.3552	-3.8385	16.5693	4.69E-05	0.0112
**ENST00000622996**	ENSG00000279432	17	2639296	2642418	-	TEC	Yes	-	-1.4265	-1.2973	16.5357	4.77E-05	0.0112
**ENST00000528520**	ENSG00000255097	11	9242447	9245509	-	lncRNA	Yes	-	-1.5148	-2.6501	16.5265	4.80E-05	0.0113
**ENST00000384519**	ENSG00000207248	16	21642053	21642160	RNU6-1005P	snRNA	Yes	-	-1.4227	-4.0477	16.4964	4.87E-05	0.0114
**ENST00000260315**	ENSG00000137757	11	104994242	105023168	CASP5	protein_coding	Yes	NM_004347.5	-1.6464	1.6749	16.4537	4.99E-05	0.0115
**ENST00000361842**	ENSG00000132530	17	6756045	6775647	XAF1	protein_coding	Yes	NM_017523.5	-1.6567	4.1586	16.3962	5.14E-05	0.0116
**ENST00000369645**	ENSG00000155363	1	112674658	112700739	MOV10	protein_coding	Yes	NM_001321324.2	-1.1643	0.8428	16.3462	5.28E-05	0.0118
**ENST00000410180**	ENSG00000222112	1	33336565	33336864	RN7SKP16	misc_RNA	Yes	-	-1.1429	-1.2923	16.3412	5.29E-05	0.0118
**ENST00000445539**	ENSG00000237827	10	103125016	103125416	RPS15AP29	processed_pseudogene	Yes	-	-1.1061	-1.2332	16.3363	5.30E-05	0.0118
**ENST00000525445**	ENSG00000185507	11	612554	615950	IRF7	protein_coding	Yes	NM_001572.5	-1.6143	1.8173	16.3439	5.28E-05	0.0118
**ENST00000499986**	ENSG00000238035	5	181306501	181324130	-	lncRNA	Yes	-	-1.1586	-2.0065	16.2834	5.45E-05	0.0120
**ENST00000580891**	ENSG00000266805	18	9506241	9509726	-	lncRNA	Yes	-	-1.2231	-1.9824	16.1685	5.80E-05	0.0123
**ENST00000579737**	ENSG00000265531	1	143874792	143883575	FCGR1CP	unprocessed_pseudogene	Yes	-	-1.7383	0.1398	16.1462	5.86E-05	0.0124
**ENST00000433072**	ENSG00000225698	14	106790693	106791233	IGHV3-72	IG_V_gene	Yes	-	2.8147	-2.5408	15.8647	6.80E-05	0.0137
**ENST00000410005**	ENSG00000222018	21	34400111	34401072	C21orf140	protein_coding	Yes	NM_001282537.2	3.7952	-5.8064	15.7897	7.08E-05	0.0140
**ENST00000649979**	ENSG00000115267	2	162267073	162318684	IFIH1	protein_coding	Yes	NM_022168.4	-1.4866	3.7421	15.7875	7.09E-05	0.0140

**ENST00000459853**	ENSG00000240233	14	77547565	77547846	RN7SL587P	misc_RNA	Yes	-	-1.6807	-3.7676	15.7100	7.38E-05	0.0143
**ENST00000616832**	ENSG00000276863	17	80333840	80334366	-	lncRNA	Yes	-	-1.2235	-1.7794	15.6840	7.49E-05	0.0144
**ENST00000355754**	ENSG00000162654	1	89181143	89198942	GBP4	protein_coding	Yes	NM_052941.5	-1.2598	3.9151	15.6511	7.62E-05	0.0145
**ENST00000505635**	ENSG00000251158	5	69898866	69903198	-	unprocessed_pseudogene	Yes	-	-1.4472	-2.2685	15.5877	7.88E-05	0.0147
**ENST00000362760**	ENSG00000199630	6	89841328	89841424	Y_RNA	misc_RNA	Yes	-	-2.8898	-5.1410	15.5823	7.90E-05	0.0148
**ENST00000648650**	ENSG00000285994	10	12563150	12567351	-	lncRNA	Yes	-	-1.1808	0.7177	15.5237	8.15E-05	0.0150
**ENST00000547512**	ENSG00000257246	12	51124627	51125381	PHB1P19	transcribed_processed_pseudogene	Yes	-	-1.4942	-2.8668	15.4315	8.55E-05	0.0155
**ENST00000363632**	ENSG00000200502	9	37160136	37160237	Y_RNA	misc_RNA	Yes	-	-1.1016	-3.3848	15.3963	8.72E-05	0.0155
**ENST00000370473**	ENSG00000117228	1	89052318	89065208	GBP1	protein_coding	Yes	NM_002053.3	-1.6953	5.0931	15.4074	8.66E-05	0.0155
**ENST00000681370**	ENSG00000233975	1	27660383	27666279	LINC02574	lncRNA	Yes	-	-2.0146	2.4720	15.4053	8.67E-05	0.0155
**ENST00000568149**	ENSG00000260954	16	1579241	1580308	-	lncRNA	Yes	-	-3.5860	-5.9757	15.3496	8.93E-05	0.0157
**ENST00000371826**	ENSG00000119922	10	89302045	89309271	IFIT2	protein_coding	Yes	NM_001547.5	-1.8881	6.9920	15.2368	9.48E-05	0.0159
**ENST00000518507**	ENSG00000253475	8	51895956	51896374	-	lncRNA	Yes	-	-1.2807	-2.7917	15.1855	9.74E-05	0.0161
**ENST00000380097**	ENSG00000121236	11	5596636	5612952	TRIM6	protein_coding	Yes	NM_001003818.3	-1.6384	-1.9709	15.1216	0.0001008	0.0163
**ENST00000225831**	ENSG00000108691	17	34255284	34257203	CCL2	protein_coding	Yes	NM_002982.4	-3.1935	-3.0809	15.1110	0.00010137	0.0164
**ENST00000531379**	ENSG00000255321	8	60551956	60553036	-	lncRNA	Yes	-	-3.2986	-6.0397	15.0759	0.00010327	0.0164
**ENST00000369168**	ENSG00000150337	1	149782693	149791675	FCGR1A	protein_coding	Yes	NM_000566.4	-1.5571	2.4726	15.0452	0.00010497	0.0165
**ENST00000451143**	ENSG00000225900	2	39098148	39098445	HSPE1P13	processed_pseudogene	Yes	-	-1.4916	-3.2013	14.9488	0.00011047	0.0168
**ENST00000409930**	ENSG00000136689	2	113127597	113134014	IL1RN	protein_coding	Yes	NM_173842.3	-1.2524	1.4092	14.8698	0.0001152	0.0171
**ENST00000433182**	ENSG00000224707	6	20437820	20440178	E2F3-IT1	lncRNA	Yes	-	-1.1654	-2.1306	14.8357	0.00011729	0.0172
**ENST00000395152**	ENSG00000213600	3	50239617	50239984	-	lncRNA	Yes	-	-1.3275	-2.5287	14.7930	0.00011998	0.0174
**ENST00000690187**	ENSG00000289557	12	104644172	104647370	-	lncRNA	Yes	-	-1.0642	-0.6448	14.7347	0.00012375	0.0177
**ENST00000623177**	ENSG00000278949	12	132865854	132867958	-	TEC	Yes	-	-1.4752	-1.8937	14.6867	0.00012694	0.0178

**ENST00000411427**	ENSG00000228318	21	41441055	41445708	-	lncRNA	Yes	-	-2.2001	-2.1970	14.6631	0.0001285 4	0.0179
**ENST00000600110**	ENSG00000268455	19	23596121	23598159	-	unprocessed_ps eudogene	Yes	-	4.1546	-5.7451	14.6201	0.0001315 1	0.0181
**ENST00000342435**	ENSG00000188313	3	146515179	146544607	PLSCR1	protein_coding	Yes	NM_021105.3	-1.4607	3.6540	14.6043	0.0001326 1	0.0181
**ENST00000393264**	ENSG00000213279	22	49572263	49575426	-	lncRNA	Yes	-	-2.1142	-2.9000	14.5770	0.0001345 5	0.0182
**ENST00000657848**	ENSG00000286505	15	84913143	84915705	-	lncRNA	Yes	-	4.3214	-5.6999	14.5688	0.0001351 3	0.0183
**ENST00000631644**	ENSG00000282508	19	209218	224007	LINC01002	lncRNA	Yes	-	-1.0541	-1.5228	14.5462	0.0001367 7	0.0184
**ENST00000510264**	ENSG00000290556	5	69607098	69624049	-	lncRNA	Yes	-	-1.0023	-0.9427	14.5187	0.0001387 8	0.0186
**ENST00000262776**	ENSG00000108679	17	78971254	78979923	LGALS3BP	protein_coding	Yes	NM_005567.4	-1.1131	-1.4523	14.5078	0.0001395 8	0.0186
**ENST00000410344**	ENSG00000222276	14	96384623	96384815	RNU2-33P	snRNA	Yes	-	-1.7313	-5.3597	14.4514	0.0001438 2	0.0187
**ENST00000560711**	ENSG00000259379	15	58152605	58154407	MTND5P32	processed_pseu dogene	Yes	-	-1.3746	-2.4060	14.4289	0.0001455 5	0.0188
**ENST00000257570**	ENSG00000135114	12	121019110	121039246	OASL	protein_coding	Yes	NM_003733.4	-1.9269	3.0728	14.2865	0.0001569 9	0.0195
**ENST00000583426**	ENSG00000264546	17	62549725	62552121	-	lncRNA	Yes	-	-1.2502	-2.8138	14.2085	0.0001636 3	0.0199
**ENST00000458392**	ENSG00000232300	17	46558829	46562795	FAM215B	lncRNA	Yes	-	-1.3978	-1.7380	14.1979	0.0001645 6	0.0200
**ENST00000686249**	ENSG00000289013	2	40394672	40396777	-	lncRNA	Yes	-	-1.1069	0.8011	14.1913	0.0001651 3	0.0200
**ENST00000535679**	ENSG00000138068	2	37167819	37188670	SULT6B1	protein_coding	Yes	NM_00136755 1.1	4.3467	-5.6737	14.0949	0.0001738 1	0.0205
**ENST00000490686**	ENSG00000240382	2	89117341	89117844	IGKV1-17	IG_V_gene	Yes	-	1.7448	-2.3305	14.0868	0.0001745 7	0.0206
**ENST00000373414**	ENSG00000288705	2	233712906	233773300	UGT1A5	protein_coding	Yes	NM_019078.2	3.9727	-5.5209	14.0643	0.0001766 6	0.0207
**ENST00000413552**	ENSG00000234358	7	111971221	111971988	RPL7AP42	processed_pseu dogene	Yes	-	-1.5134	-3.0084	14.0284	0.0001800 7	0.0209
**ENST00000256722**	ENSG00000134326	2	6848315	6865907	CMPK2	protein_coding	Yes	NM_207315.4	-1.8830	3.2149	13.9515	0.0001875 9	0.0214
**ENST00000606809**	ENSG00000272491	1	48227887	48229561	-	lncRNA	Yes	-	-2.2670	-3.4014	13.9187	0.0001908 8	0.0215
**ENST00000226299**	ENSG00000002549	4	17577197	17607970	LAP3	protein_coding	Yes	NM_015907.3	-1.6875	4.4368	13.8338	0.0001997 1	0.0220
**ENST00000666887**	ENSG00000261465	16	19062143	19067432	COQ7-DT	lncRNA	Yes	-	2.9002	-5.6330	13.8280	0.0002003 2	0.0221

**ENST00000341491**	ENSG00000162772	1	212608760	212620775	ATF3	protein_coding	Yes	NM_001674.4	-2.0090	-1.7721	13.7971	0.00020366	0.0221
**ENST00000379883**	ENSG00000107201	9	32455301	32526196	DDX58	protein_coding	Yes	NM_014314.4	-1.4736	5.3788	13.7545	0.00020832	0.0224
**ENST00000513164**	ENSG00000248477	5	69618312	69623446	NAIPP3	transcribed_unprocessed_pseudogene	Yes	-	-1.1271	-1.3957	13.6973	0.00021476	0.0228
**ENST00000687046**	ENSG00000288995	9	126819281	126820987	-	lncRNA	Yes	-	-1.3221	-3.1324	13.6796	0.0002168	0.0229
**ENST00000510918**	ENSG00000249237	1	84344677	84349661	-	transcribed_unitary_pseudogene	Yes	-	3.3379	-5.9322	13.5963	0.00022664	0.0234
**ENST00000385573**	ENSG00000208308	2	135136627	135136755	SNORA40B	snoRNA	Yes	-	-1.9418	-5.1517	13.5357	0.00023407	0.0238
**ENST00000582634**	ENSG00000264653	8	130008333	130008453	MIR5194	miRNA	Yes	-	-1.2128	-2.3340	13.4550	0.00024435	0.0244
**ENST00000493797**	ENSG00000239906	1	139789	140339	-	lncRNA	Yes	-	-1.4867	-1.3247	13.4144	0.0002497	0.0247
**ENST00000603719**	ENSG00000271687	5	100046976	100048785	MTND5P10	processed_pseudogene	Yes	-	3.9785	-5.7883	13.3927	0.0002526	0.0248
**ENST00000482888**	ENSG00000241358	3	146395739	146416577	-	transcribed_processed_pseudogene	Yes	-	3.7551	-5.8360	13.3728	0.0002553	0.0249
**ENST00000370459**	ENSG00000154451	1	89256188	89272860	GBP5	protein_coding	Yes	NM_052942.5	-1.4501	6.5829	13.2529	0.00027216	0.0256
**ENST00000518237**	ENSG00000131203	8	39913890	39928790	IDO1	protein_coding	Yes	NM_002164.6	-1.7940	0.8456	13.2476	0.00027293	0.0256
**ENST00000516107**	ENSG00000251916	6	32549939	32550090	RNU1-61P	snRNA	Yes	-	-3.3558	-5.5633	13.2298	0.00027553	0.0258
**ENST00000365280**	ENSG00000202150	20	35030316	35030420	RNU6-407P	snRNA	Yes	-	-1.4027	-4.2589	13.2079	0.00027877	0.0258
**ENST00000373409**	ENSG00000244474	2	233718735	233773300	UGT1A4	protein_coding	Yes	NM_007120.3	4.2192	-5.4316	13.2009	0.00027982	0.0259
**ENST00000444853**	ENSG00000225195	12	64628343	64629976	-	lncRNA	Yes	-	-1.2962	-2.2504	13.1624	0.00028562	0.0261
**ENST00000681750**	ENSG00000288684	9	32455322	32552568	-	protein_coding	Yes	-	-1.4118	5.4238	13.1159	0.00029279	0.0263
**ENST00000262101**	ENSG00000038945	8	16107880	16192651	MSR1	protein_coding	Yes	NM_138715.3	-1.2525	-0.2621	13.0583	0.00030194	0.0267
**ENST00000474629**	ENSG00000173193	3	122680838	122730840	PARP14	protein_coding	Yes	NM_017554.3	-1.1353	6.7878	13.0588	0.00030187	0.0267
**ENST00000689708**	ENSG00000288948	2	101797889	101800135	-	lncRNA	Yes	-	-1.1942	-1.7560	12.9666	0.0003171	0.0275
**ENST00000664313**	ENSG00000287909	16	53544338	53545689	-	lncRNA	Yes	-	2.9241	-5.6136	12.9280	0.0003237	0.0278

**ENST00000318238**	ENSG00000177409	7	93130055	93148385	SAMD9L	protein_coding	Yes	NM_152703.5	-1.3521	5.9870	12.8365	0.00033992	0.0286
**ENST00000505679**	ENSG00000250381	4	4143457	4150421	UNC93B4	unprocessed_pseudogene	Yes	-	4.6069	-5.5936	12.8215	0.00034265	0.0287
**ENST00000507871**	ENSG00000250727	8	124141887	124143512	-	processed_pseudogene	Yes	-	4.0084	-5.7997	12.7431	0.00035733	0.0292
**ENST00000464848**	ENSG00000105948	7	139133777	139191986	TTC26	protein_coding	Yes	NM_024926.4	-1.1846	-0.0347	12.7213	0.00036152	0.0294
**ENST00000621902**	ENSG00000276015	11	2697850	2698076	-	misc_RNA	Yes	-	-1.8773	-4.3913	12.6534	0.00037488	0.0299
**ENST00000431069**	ENSG00000235917	9	5098340	5099325	MTCO2P11	unprocessed_pseudogene	Yes	-	-1.4430	-4.0484	12.5924	0.00038732	0.0305
**ENST00000659444**	ENSG00000248176	4	29118303	29211696	-	lncRNA	Yes	-	3.2531	-5.9643	12.5519	0.00039581	0.0307
**ENST00000376819**	ENSG00000171819	1	11189354	11195981	ANGPTL7	protein_coding	Yes	NM_021146.4	2.5709	-5.3502	12.5439	0.0003975	0.0307
**ENST00000373426**	ENSG00000244122	2	233681900	233773300	UGT1A7	protein_coding	Yes	NM_019077.3	3.4511	-5.4790	12.5399	0.00039836	0.0307
**ENST00000336125**	ENSG00000185829	17	46552755	46579691	ARL17A	protein_coding	Yes	NM_001113738.2	-1.1067	0.8706	12.5320	0.00040004	0.0308
**ENST00000330714**	ENSG00000183486	21	41362026	41409393	MX2	protein_coding	Yes	NM_002463.2	-1.0014	4.2573	12.5227	0.00040203	0.0309
**ENST00000431518**	ENSG00000229054	7	33036211	33036382	RPS29P14	processed_pseudogene	Yes	-	-1.5347	-3.1951	12.5127	0.00040419	0.0310
**ENST00000375887**	ENSG00000102524	13	108269717	108308478	TNFSF13B	protein_coding	Yes	NM_006573.5	-1.2603	4.4972	12.5093	0.00040493	0.0310
**ENST00000397747**	ENSG00000197646	9	5510530	5571282	PDCD1LG2	protein_coding	Yes	NM_025239.4	-1.7063	-2.0502	12.4494	0.00041813	0.0314
**ENST00000284885**	ENSG00000154646	21	18269115	18403785	TMPRSS15	protein_coding	Yes	NM_002772.3	3.1432	-4.8544	12.4287	0.00042279	0.0316
**ENST00000653887**	ENSG00000233559	7	130853731	130930680	LINC00513	lncRNA	Yes	-	-1.3517	-3.9775	12.4165	0.00042557	0.0316
**ENST00000532626**	ENSG00000254964	11	62606160	62606405	-	lncRNA	Yes	-	2.9361	-6.0333	12.4002	0.00042929	0.0318
**ENST00000533669**	ENSG00000255311	11	83645729	83725390	DLG2-AS2	lncRNA	Yes	-	3.6072	-5.8929	12.3837	0.0004331	0.0319
**ENST00000465940**	ENSG00000243175	4	143425051	143425920	RPSAP36	transcribed_processed_pseudogene	Yes	-	-1.0165	-2.6559	12.3727	0.00043565	0.0320
**ENST00000509999**	ENSG00000251409	5	95835520	95852721	-	lncRNA	Yes	-	3.9032	-5.7966	12.3650	0.00043745	0.0320
**ENST00000414673**	ENSG00000227300	17	16829998	16832753	KRT16P2	transcribed_unprocessed_pseudogene	Yes	-	4.0702	-5.7698	12.2992	0.00045316	0.0325

**ENST00000331982**	ENSG00000183709	19	39268395	39270188	IFNL2	protein_coding	Yes	NM_172138.2	4.1308	-5.7485	12.2862	0.00045632	0.0326
**ENST00000398297**	ENSG00000215120	X	71527813	71530225	SOCS6P1	processed_pseudogene	Yes	-	3.2621	-5.9644	12.2687	0.00046062	0.0327
**ENST00000614007**	ENSG00000278757	1	516375	516479	U6	snRNA	Yes	-	-2.2990	-4.4319	12.2452	0.00046646	0.0330
**ENST00000402856**	ENSG00000219409	6	144397958	144398752	-	processed_pseudogene	Yes	-	-1.0768	-2.6682	12.2249	0.00047155	0.0331
**ENST00000612985**	ENSG00000277610	1	120942599	120942763	RNVU1-4	snRNA	Yes	-	-1.7490	-4.4020	12.2205	0.00047268	0.0331
**ENST00000610012**	ENSG00000272784	4	183094422	183095056	-	lncRNA	Yes	-	3.2987	-5.9601	12.2072	0.00047606	0.0331
**ENST00000455707**	ENSG00000224789	2	120174884	120216544	-	lncRNA	Yes	-	-1.9296	-3.0440	12.1694	0.00048581	0.0335
**ENST00000460552**	ENSG00000242330	14	71448688	71448958	RN7SL683P	misc_RNA	Yes	-	-1.2707	-2.9295	12.1648	0.00048701	0.0335
**ENST00000616960**	ENSG00000274840	3	18013225	18041603	BALR6	lncRNA	Yes	-	3.2616	-5.9637	12.1656	0.0004868	0.0335
**ENST00000648023**	ENSG00000225362	15	71110243	71115494	CT62	lncRNA	Yes	-	3.9657	-4.8086	12.1661	0.00048666	0.0335
**ENST00000670291**	ENSG00000239445	3	98706235	98732757	ST3GAL6-AS1	lncRNA	Yes	-	1.1974	-3.3527	12.1679	0.00048618	0.0335
**ENST00000648074**	ENSG00000285850	16	17453469	17456236	-	lncRNA	Yes	-	-2.3979	-5.4233	12.1389	0.00049382	0.0338
**ENST00000361066**	ENSG00000134202	1	109733936	109741038	GSTM3	protein_coding	Yes	NM_000849.5	1.2634	-0.6617	12.1273	0.00049688	0.0339
**ENST00000442828**	ENSG00000227782	17	16040471	16041273	-	lncRNA	Yes	-	-1.1665	-3.7633	12.1218	0.00049836	0.0339
**ENST00000517095**	ENSG00000252904	5	65961182	65961319	-	snoRNA	Yes	-	-2.3951	-5.7733	12.0670	0.00051322	0.0343
**ENST00000488827**	ENSG00000242251	22	31757201	31757498	RN7SL20P	misc_RNA	Yes	-	-1.5826	-4.9610	12.0564	0.00051616	0.0344
**ENST00000609583**	ENSG00000273481	1	151327948	151328429	-	lncRNA	Yes	-	1.9183	-5.2842	12.0498	0.00051797	0.0345
**ENST00000437128**	ENSG00000237568	1	89260581	89269754	-	lncRNA	Yes	-	-1.4259	0.8610	12.0301	0.00052348	0.0346
**ENST00000423256**	ENSG00000228403	10	48878021	48878649	-	lncRNA	Yes	-	-1.2965	-3.4507	12.0128	0.00052835	0.0348
**ENST00000394834**	ENSG00000203943	1	84301707	84350798	SAMD13	protein_coding	Yes	NM_001134663.2	1.3977	-4.2403	12.0091	0.00052941	0.0349
**ENST00000703003**	ENSG00000289251	7	45187004	45192605	-	lncRNA	Yes	-	-1.1935	-3.5543	11.9581	0.00054411	0.0353
**ENST00000556397**	ENSG00000258654	15	20344735	20359159	-	lncRNA	Yes	-	3.9696	-5.7673	11.9404	0.00054929	0.0354

**ENST00000638164**	ENSG00000214725	16	29864307	29865442	CDIPTOSP	transcribed_unitary_pseudogene	Yes	-	3.1791	-5.9717	11.9170	0.00055623	0.0355
**ENST00000581978**	ENSG00000266467	10	68007717	68008017	RN7SL220P	misc_RNA	Yes	-	-1.2461	-3.3069	11.9133	0.00055736	0.0356
**ENST00000510153**	ENSG00000251093	5	91226474	91227071	-	lncRNA	Yes	-	-1.1108	-2.7377	11.8971	0.00056222	0.0356
**ENST00000269127**	ENSG00000141371	17	60422488	60431426	C17orf64	protein_coding	Yes	NM_181707.3	3.9338	-5.7954	11.8914	0.00056394	0.0357
**ENST00000575953**	ENSG00000262514	16	68199794	68200981	-	lncRNA	Yes	-	-1.3086	-2.5033	11.8932	0.0005634	0.0357
**ENST00000553704**	ENSG00000258609	18	57054557	57072119	LINC-ROR	lncRNA	Yes	-	3.9649	-5.7932	11.8816	0.0005669	0.0358
**ENST00000314586**	ENSG00000179044	16	67184378	67190136	EXOC3L1	protein_coding	Yes	NM_178516.4	-1.9278	-0.7985	11.8658	0.00057175	0.0359
**ENST00000404620**	ENSG00000218689	6	129756297	129757112	RPL5P21	processed_pseudogene	Yes	-	3.7698	-5.8783	11.8599	0.00057355	0.0360
**ENST00000362134**	ENSG00000284190	17	59841265	59841337	MIR21	miRNA	Yes	-	-1.1210	-3.6530	11.8417	0.00057918	0.0361
**ENST00000314128**	ENSG00000170581	12	56341596	56360107	STAT2	protein_coding	Yes	NM_005419.4	-1.0527	4.2825	11.7891	0.00059579	0.0365
**ENST00000455032**	ENSG00000234984	1	166665884	166682196	FMO10P	unprocessed_pseudogene	Yes	-	4.5162	-5.6662	11.7885	0.00059598	0.0365
**ENST00000554335**	ENSG00000020577	14	54567127	54793312	SAMD4A	protein_coding	Yes	NM_015589.6	-1.2370	0.2847	11.7805	0.00059854	0.0366
**ENST00000665823**	ENSG00000287483	6	20329921	20334411	-	lncRNA	Yes	-	2.9710	-6.0279	11.7646	0.00060368	0.0367
**ENST00000265440**	ENSG00000105967	7	115935151	116030763	TFEC	protein_coding	Yes	NM_012252.4	-1.3489	3.6137	11.6917	0.00062779	0.0373
**ENST00000666287**	ENSG00000287141	2	112061083	112063709	-	lncRNA	Yes	-	3.1517	-5.4579	11.6705	0.00063499	0.0375
**ENST00000263321**	ENSG00000077498	11	89177874	89295759	TYR	protein_coding	Yes	NM_000372.5	4.0377	-5.7987	11.6662	0.00063648	0.0375
**ENST00000583549**	ENSG00000263407	8	9048444	9048518	MIR4660	miRNA	Yes	-	-3.2288	-6.0611	11.6562	0.00063991	0.0376
**ENST00000687904**	ENSG00000289192	1	101964792	101967614	-	lncRNA	Yes	-	3.8769	-5.8151	11.6545	0.00064049	0.0376
**ENST00000523525**	ENSG00000254227	8	125998993	126001001	-	lncRNA	Yes	-	3.7923	-5.8492	11.6397	0.00064557	0.0377
**ENST00000378574**	ENSG00000232241	20	17479084	17479431	DYNLT3P1	processed_pseudogene	Yes	-	2.8498	-5.8386	11.6314	0.00064848	0.0378
**ENST00000662327**	ENSG00000287589	1	239205137	239212284	-	lncRNA	Yes	-	4.2106	-5.7553	11.5723	0.0006694	0.0384
**ENST00000581881**	ENSG00000263642	4	40502039	40502119	MIR4802	miRNA	Yes	-	-1.5365	-4.0604	11.5433	0.00067993	0.0386

**ENST00000364695**	ENSG00000201565	7	67297652	67297754	Y_RNA	misc_RNA	Yes	-	2.9051	-6.0378	11.5288	0.00068526	0.0388
**ENST00000410247**	ENSG00000222179	18	45989652	45989945	RN7SKP26	misc_RNA	Yes	-	-1.4611	-4.7111	11.4540	0.0007134	0.0397
**ENST00000454299**	ENSG00000236825	9	62606796	62607748	RAB28P2	processed_pseudogene	Yes	-	3.9833	-5.8014	11.4398	0.00071889	0.0399
**ENST00000603980**	ENSG00000270532	2	85341280	85341838	PEBP1P2	processed_pseudogene	Yes	-	1.2225	-4.5043	11.4114	0.00072994	0.0402
**ENST00000361644**	ENSG00000198798	X	30230656	30237495	MAGEB3	protein_coding	Yes	NM_002365.5	4.1473	-5.7633	11.3959	0.00073608	0.0404
**ENST00000225275**	ENSG00000005381	17	58269854	58280935	MPO	protein_coding	Yes	NM_000250.2	1.2428	0.1289	11.3750	0.0007444	0.0406
**ENST00000702521**	ENSG00000290022	4	139841167	139841542	-	lncRNA	Yes	-	-1.1246	-3.4074	11.3642	0.00074872	0.0407
**ENST00000613359**	ENSG00000275215	21	8395606	8395759	RNA5-8SN3	rRNA	Yes	-	2.5161	-1.7416	11.3612	0.00074994	0.0407
**ENST00000670499**	ENSG00000267215	18	59081910	59085916	-	lncRNA	Yes	-	3.9343	-5.8234	11.3535	0.00075306	0.0407
**ENST00000649774**	ENSG00000285899	X	43279559	43438657	-	lncRNA	Yes	-	3.8528	-5.8317	11.3500	0.00075447	0.0408
**ENST00000519981**	ENSG00000253281	8	58255770	58272101	-	lncRNA	Yes	-	4.0405	-5.7959	11.3470	0.00075568	0.0408
**ENST00000436423**	ENSG00000232464	22	25959073	25983526	-	lncRNA	Yes	-	3.5680	-5.8992	11.3416	0.00075789	0.0408
**ENST00000690581**	ENSG00000289371	17	70191242	70193052	-	lncRNA	Yes	-	-1.1694	-3.9630	11.3179	0.00076765	0.0411
**ENST00000424412**	ENSG00000224067	9	111802979	111803435	-	processed_pseudogene	Yes	-	-1.1395	-3.6630	11.3155	0.00076861	0.0411
**ENST00000372492**	ENSG00000243710	1	43172329	43254358	CFAP57	protein_coding	Yes	NM_001378189.1	1.7117	-4.3853	11.2916	0.00077861	0.0414
**ENST00000579909**	ENSG00000265195	15	68801849	68801925	MIR4312	miRNA	Yes	-	-1.1918	-3.1929	11.2935	0.00077781	0.0414
**ENST00000691951**	ENSG00000289631	11	125112013	125113099	-	lncRNA	Yes	-	1.1920	-4.5699	11.2962	0.00077664	0.0414
**ENST00000517571**	ENSG00000253265	2	89078009	89078784	IGKV2-14	IG_V_pseudogene	Yes	-	3.1778	-5.9846	11.2743	0.00078587	0.0416
**ENST00000342609**	ENSG00000229023	2	190992638	190993567	RAB1AP1	processed_pseudogene	Yes	-	-1.1770	-2.8052	11.2667	0.00078908	0.0416
**ENST00000582765**	ENSG00000266497	17	46503945	46509339	RDM1P2	unprocessed_pseudogene	Yes	-	-1.2261	-1.2786	11.2671	0.00078895	0.0416
**ENST00000577684**	ENSG00000220161	17	18411158	18414380	LINC02076	lncRNA	Yes	-	2.3731	-4.4002	11.2405	0.00080032	0.0420
**ENST00000570082**	ENSG00000260670	3	72321050	72324027	-	lncRNA	Yes	-	3.6545	-5.8760	11.2210	0.00080878	0.0422

**ENST00000556978**	ENSG00000258693	14	99977114	99978098	-	lncRNA	Yes	-	3.3348	-5.9451	11.1807	0.00082651	0.0427
**ENST00000405234**	ENSG00000220586	6	39992814	40000233	TUBBP9	transcribed_processed_pseudogene	Yes	-	3.3877	-5.9131	11.1281	0.00085032	0.0433
**ENST00000508806**	ENSG00000251568	5	5375722	5376836	ALG3P1	processed_pseudogene	Yes	-	-3.0104	-6.0909	11.1256	0.00085142	0.0433
**ENST00000626977**	ENSG00000280543	8	130082737	130084768	ASAP1-IT2	lncRNA	Yes	-	-1.3552	-2.7755	11.1198	0.0008541	0.0434
**ENST00000363205**	ENSG00000200075	8	129868589	129868716	-	snoRNA	Yes	-	-1.0746	-3.4014	11.0620	0.00088117	0.0440
**ENST00000510230**	ENSG00000249639	5	135450612	135458697	-	lncRNA	Yes	-	2.6956	-5.5837	11.0510	0.00088641	0.0441
**ENST00000409874**	ENSG00000156414	14	103928455	104052667	TDRD9	protein_coding	Yes	NM_153046.3	-1.0904	0.5752	11.0478	0.00088792	0.0441
**ENST00000674143**	ENSG00000288536	21	15102312	15138649	-	lncRNA	Yes	-	4.3802	-5.6834	11.0387	0.0008923	0.0442
**ENST00000478664**	ENSG00000240589	17	44409058	44409358	RN7SL258P	misc_RNA	Yes	-	-1.3040	-3.6995	11.0275	0.00089772	0.0443
**ENST00000629816**	ENSG00000091879	8	6499631	6563245	ANGPT2	protein_coding	Yes	NM_001118887.2	1.1590	-3.9131	10.9693	0.00092632	0.0450
**ENST00000313624**	ENSG00000133106	13	42886387	42992241	EPSTI1	protein_coding	Yes	NM_033255.5	-1.3816	4.0830	10.9656	0.0009282	0.0451
**ENST00000337530**	ENSG00000165185	9	112486826	112669397	KIAA1958	protein_coding	Yes	NM_133465.4	-1.0026	0.2984	10.9148	0.00095398	0.0456
**ENST00000447834**	ENSG00000206552	3	42934251	42936785	KRBOX1-AS1	lncRNA	Yes	-	3.8000	-5.4879	10.9060	0.00095851	0.0457
**ENST00000595059**	ENSG00000269473	19	58440447	58445849	ZNF132-DT	lncRNA	Yes	-	1.6264	-4.0694	10.8920	0.0009658	0.0459
**ENST00000600665**	ENSG00000268636	19	49625993	49626439	-	lncRNA	Yes	-	3.7119	-5.8876	10.8884	0.00096771	0.0460
**ENST00000411797**	ENSG00000225000	7	18429061	18430738	-	lncRNA	Yes	-	4.2833	-5.7094	10.8739	0.00097531	0.0462
**ENST00000590850**	ENSG00000267637	17	59784812	59785035	-	lncRNA	Yes	-	-1.3824	-2.7647	10.8694	0.00097766	0.0462
**ENST00000392713**	ENSG00000213172	1	40364765	40365183	-	processed_pseudogene	Yes	-	-1.8120	-4.4172	10.8208	0.00100363	0.0470
**ENST00000508112**	ENSG00000249341	4	53659207	53737156	-	lncRNA	Yes	-	3.6693	-5.8907	10.7868	0.00102228	0.0475
**ENST00000547580**	ENSG00000257169	12	96386817	96387194	-	processed_pseudogene	Yes	-	-1.2001	-2.6230	10.7847	0.00102345	0.0475
**ENST00000243347**	ENSG00000123610	2	151357591	151380046	TNFAIP6	protein_coding	Yes	NM_007115.4	-1.5969	2.7937	10.7695	0.00103185	0.0477

**ENST00000634410**	ENSG00000240661	3	120357127	120367874	LRRC58-DT	transcribed_unitary_pseudogene	Yes	-	4.8636	-3.8683	10.7649	0.00103445	0.0478
**ENST00000682922**	ENSG00000181381	4	168356734	168480492	DDX60L	protein_coding	Yes	NM_001012967.3	-1.1232	5.9401	10.7423	0.00104715	0.0480
**ENST00000685440**	ENSG00000288940	4	30717368	30718929	-	lncRNA	Yes	-	4.0985	-5.4261	10.7261	0.00105637	0.0482
**ENST00000263621**	ENSG00000197561	19	852302	856243	ELANE	protein_coding	Yes	NM_001972.4	1.5894	-1.6005	10.7248	0.0010571	0.0482
**ENST00000648470**	ENSG00000285562	11	18142340	18145142	-	lncRNA	Yes	-	4.3727	-5.6752	10.7230	0.00105815	0.0483
**ENST00000520443**	ENSG00000253965	5	140347867	140357794	-	lncRNA	Yes	-	3.6843	-5.6219	10.7136	0.00106348	0.0484
**ENST00000585369**	ENSG00000267263	17	77469067	77471045	-	lncRNA	Yes	-	-1.4085	-3.3164	10.7095	0.00106588	0.0484
**ENST00000420915**	ENSG00000259849	X	26557674	26558330	VENTXP1	transcribed_processed_pseudogene	Yes	-	4.3740	-5.6981	10.7044	0.00106884	0.0485
**ENST00000567093**	ENSG00000260417	16	85592265	85595720	-	lncRNA	Yes	-	-1.2337	-2.3671	10.7009	0.00107085	0.0485
**ENST00000595809**	ENSG00000268580	2	144877733	144882033	LINC01966	lncRNA	Yes	-	3.0860	-6.0191	10.6909	0.00107664	0.0487
**ENST00000421645**	ENSG00000227914	9	673477	685555	-	lncRNA	Yes	-	3.5747	-5.9250	10.6867	0.00107908	0.0487
**ENST00000357068**	ENSG00000100033	22	18912780	18936293	PRODH	protein_coding	Yes	NM_016335.6	3.3865	-5.3336	10.6508	0.00110022	0.0493
**ENST00000264346**	ENSG00000138642	4	88378851	88443097	HERC6	protein_coding	Yes	NM_017912.4	-1.0814	2.2218	10.6476	0.00110215	0.0493
**ENST00000686452**	ENSG00000289102	19	52558016	52559570	-	lncRNA	Yes	-	-1.0283	-2.0123	10.6468	0.00110259	0.0493
**ENST00000552900**	ENSG00000258262	12	92530459	92531324	-	processed_pseudogene	Yes	-	2.9455	-6.0422	10.6225	0.00111722	0.0497
**ENST00000648447**	ENSG00000285885	3	39494836	39502567	-	lncRNA	Yes	-	3.6571	-5.3939	10.6235	0.00111658	0.0497
**ENST00000322630**	ENSG00000178404	17	78890578	78903201	CEP295NL	protein_coding	Yes	NM_001243540.2	-1.0659	-1.2072	10.6092	0.00112529	0.0498

Supplementary table 2: Differentially expressed genes (MwoA patients vs. controls). Log_2FC – Log 2-fold change; logCPM – log counts per million; LR – likelihood ratio; FDR – False discovery rate

Transcript	Trancript stable ID	chr	start	end	Gene name	Gene type	Canonical	MANE.Select	log_2FC	logCPM	LR	P-Value	FDR
**ENST00000305208**	ENSG00000241635	2	233760269	233773300	UGT1A1	protein_coding	Yes	NM_000463.3	5.2483	-5.4231	20.9475	4.72E-06	0.0495
**ENST00000373450**	ENSG00000242366	2	233617632	233773300	UGT1A8	protein_coding	Yes	NM_019076.5	5.3850	-5.3347	23.1175	1.52E-06	0.0495
**ENST00000390295**	ENSG00000211649	22	22369613	22370087	IGLV7-46	IG_V_gene	Yes	-	3.1923	-1.6674	26.3275	2.88E-07	0.0495
**ENST00000433072**	ENSG00000225698	14	106790693	106791233	IGHV3-72	IG_V_gene	Yes	-	3.4279	-2.2927	22.4000	2.21E-06	0.0495
**ENST00000656637**	ENSG00000228358	4	117371529	117412712	LINC02263	lncRNA	Yes	-	4.4295	-5.6430	20.2789	6.69E-06	0.0495

Supplementary table 3: Differentially expressed genes (Female migraine patients vs. Female controls). Log_2FC – Log 2-fold change; logCPM – log counts per million; LR – likelihood ratio; FDR – False discovery rate

Transcript	Transcript stable ID	chr	start	end	Gene name	Gene type	Canonical	MANE Select	log_2FC	logCPM	LR	P-Value	FDR
**ENST00000315576**	ENSG00000127507	19	14732391	14778560	ADGRE2	protein_coding	Yes	NM_013447.4	-1.0647	3.5665	37.5570	8.9E-10	9.1E-05
**ENST00000433113**	ENSG00000240527	10	95833507	95873758	-	lncRNA	Yes	-	-1.8012	-1.2207	38.8702	4.5E-10	9.1E-05
**ENST00000547512**	ENSG00000257246	12	51124627	51125381	PHB1P19	transcribed_processed_pseudogene	Yes	-	-2.7343	-2.4970	35.4674	2.6E-09	1.3E-04
**ENST00000689584**	ENSG00000181218	1	228457363	228457873	H2AW	protein_coding	Yes	NM_033445.3	1.3953	-0.0192	33.9668	5.6E-09	1.8E-04
**ENST00000400299**	ENSG00000198832	22	31104776	31107568	SELENOM	protein_coding	Yes	NM_080430.4	1.0420	-0.4110	29.9827	4.4E-08	3.7E-04
**ENST00000233057**	ENSG00000055332	2	37099209	37156980	EIF2AK2	protein_coding	Yes	NM_001135651.3	-1.0051	3.9527	28.7630	8.2E-08	4.5E-04
**ENST00000454486**	ENSG00000223509	15	32523025	32536926	WHAMMP1	transcribed_processed_pseudogene	Yes	-	1.1051	4.5360	27.1827	1.9E-07	6.9E-04
**ENST00000518552**	ENSG00000254325	8	55893594	55895739	-	lncRNA	Yes	-	-2.1079	-0.2097	27.1601	1.9E-07	6.9E-04
**ENST00000466086**	ENSG00000234975	1	228687414	228687826	FTH1P2	processed_pseudogene	Yes	-	-1.4023	-1.6960	26.6555	2.4E-07	7.4E-04
**ENST00000342058**	ENSG00000140092	14	91869410	91947694	FBLN5	protein_coding	Yes	NM_006329.4	1.3030	-0.5096	26.5832	2.5E-07	7.5E-04
**ENST00000651111**	ENSG00000241489	X	149477103	149540926	-	protein_coding	Yes	-	-1.1975	4.2591	26.3036	2.9E-07	7.9E-04
**ENST00000258381**	ENSG00000135899	2	230165185	230219984	SP110	protein_coding	Yes	NM_080424.4	-1.1487	3.5283	25.9859	3.4E-07	8.0E-04

**ENST00000615155**	ENSG00000236438	3	198180754	198190365	FAM157A	transcribed_unprocessed_pseudogene	Yes	-	-1.5883	-0.4993	25.9100	3.6E-07	8.0E-04
**ENST00000335750**	ENSG00000021645	14	78170372	79868291	NRXN3	protein_coding	Yes	NM_001330195.2	1.7006	0.0191	25.7794	3.8E-07	8.4E-04
**ENST00000644246**	ENSG00000272449	1	2530063	2547460	-	lncRNA	Yes	-	1.0726	-0.4236	25.6009	4.2E-07	8.8E-04
**ENST00000414273**	ENSG00000237973	1	631073	632616	MTCO1P12	unprocessed_pseudogene	Yes	-	-1.1085	1.7600	25.2875	4.9E-07	9.0E-04
**ENST00000264033**	ENSG00000110395	11	119206338	119308149	CBL	protein_coding	Yes	NM_005188.4	-1.3431	4.3551	25.1741	5.2E-07	9.2E-04
**ENST00000581811**	ENSG00000274963	1	150568970	150569269	RN7SL600P	misc_RNA	Yes	-	-1.7568	-0.5068	25.1543	5.3E-07	9.2E-04
**ENST00000340855**	ENSG00000010404	X	149476987	149505306	IDS	protein_coding	Yes	NM_000202.8	-1.1698	4.3474	25.0057	5.7E-07	9.4E-04
**ENST00000296591**	ENSG00000164176	5	83940553	84384880	EDIL3	protein_coding	Yes	NM_005711.5	3.5076	-2.7361	24.6215	7.0E-07	1.0E-03
**ENST00000450128**	ENSG00000227262	6	29925982	29926973	HCG4B	unprocessed_pseudogene	Yes	-	2.8438	-1.9985	24.4876	7.5E-07	1.0E-03
**ENST00000440769**	ENSG00000234292	5	91280096	91281142	-	lncRNA	Yes	-	-1.5912	-0.4001	24.3666	8.0E-07	1.1E-03
**ENST00000663752**	ENSG00000286489	10	101694400	101705119	-	lncRNA	Yes	-	2.2766	-3.0665	24.0322	9.5E-07	1.1E-03
**ENST00000429111**	ENSG00000224083	9	5096665	5098193	MTCO1P11	unprocessed_pseudogene	Yes	-	-2.3149	-2.5083	23.8020	1.1E-06	1.2E-03
**ENST00000317633**	ENSG00000172058	5	70900668	70908115	SERF1A	protein_coding	Yes	NM_022968.2	1.1522	-1.2618	23.4837	1.3E-06	1.3E-03
**ENST00000433186**	ENSG00000230789	5	143192499	143194166	ARHGAP26-IT1	lncRNA	Yes	-	-1.2704	-0.6091	23.4040	1.3E-06	1.4E-03
**ENST00000327134**	ENSG00000180370	3	196739856	196832647	PAK2	protein_coding	Yes	NM_002577.4	-1.0730	4.8238	23.2041	1.5E-06	1.4E-03
**ENST00000611191**	ENSG00000273797	14	69617121	69617648	-	lncRNA	Yes	-	-1.0917	-1.0230	23.0850	1.5E-06	1.4E-03
**ENST00000369096**	ENSG00000057657	6	106086335	106109938	PRDM1	protein_coding	Yes	NM_001198.4	-1.0287	2.1691	22.7343	1.9E-06	1.5E-03
**ENST00000580891**	ENSG00000266805	18	9506241	9509726	-	lncRNA	Yes	-	-1.7920	-1.5560	22.7001	1.9E-06	1.5E-03
**ENST00000288502**	ENSG00000157693	9	114611290	114646422	TMEM268	protein_coding	Yes	NM_153045.4	-1.0904	0.5214	22.5885	2.0E-06	1.5E-03
**ENST00000393087**	ENSG00000197249	14	94376746	94388602	SERPINA1	protein_coding	Yes	NM_000295.5	-1.3665	3.9979	22.5460	2.1E-06	1.5E-03
**ENST00000524091**	ENSG00000253570	8	38600660	38601200	RNF5P1	processed_pseudogene	Yes	-	1.1790	0.1441	22.2239	2.4E-06	1.6E-03
**ENST00000343986**	ENSG00000171858	20	62387102	62388520	RPS21	protein_coding	Yes	NM_001024.4	1.3362	4.9609	21.6413	3.3E-06	1.9E-03

**ENST00000634862**	ENSG00000290937	3	198167738	198222513	-	lncRNA	Yes	-	-1.6093	1.4535	21.5515	3.4E-06	1.9E-03
**ENST00000449672**	ENSG00000230539	7	36597833	36600120	AOAH-IT1	lncRNA	Yes	-	-1.1573	-0.1895	21.2743	4.0E-06	2.1E-03
**ENST00000525634**	ENSG00000110700	11	17074387	17077667	RPS13	protein_coding	Yes	NM_001017.3	1.0221	5.5310	21.2244	4.1E-06	2.1E-03
**ENST00000437890**	ENSG00000228929	1	52772193	52772648	RPS13P2	processed_pseudogene	Yes	-	1.0015	3.6716	21.0081	4.6E-06	2.2E-03
**ENST00000590850**	ENSG00000267637	17	59784812	59785035	-	lncRNA	Yes	-	-1.8908	-2.3136	20.9434	4.7E-06	2.3E-03
**ENST00000420353**	ENSG00000170653	12	53512053	53626382	ATF7	protein_coding	Yes	NM_006856.3	-1.3702	0.8503	20.8675	4.9E-06	2.3E-03
**ENST00000687452**	ENSG00000259366	8	29055928	29057395	-	lncRNA	Yes	-	-2.1677	-0.8891	20.8695	4.9E-06	2.3E-03
**ENST00000691717**	ENSG00000289353	16	66567155	66568898	-	lncRNA	Yes	-	-1.0905	1.6477	20.7231	5.3E-06	2.4E-03
**ENST00000515156**	ENSG00000250138	5	69631962	69636399	-	unprocessed_pseudogene	Yes	-	-2.5187	-1.0957	20.5268	5.9E-06	2.6E-03
**ENST00000495392**	ENSG00000227081	12	3211662	3211917	-	processed_pseudogene	Yes	-	1.9058	6.9239	20.4681	6.1E-06	2.6E-03
**ENST00000578539**	ENSG00000264853	17	75370946	75373736	-	lncRNA	Yes	-	-1.5605	0.6649	20.4768	6.0E-06	2.6E-03
**ENST00000624318**	ENSG00000279476	16	81517823	81520617	-	TEC	Yes	-	-1.7182	0.0819	20.3878	6.3E-06	2.7E-03
**ENST00000338222**	ENSG00000188021	X	56563626	56567868	UBQLN2	protein_coding	Yes	NM_013444.4	-1.0393	1.9174	20.3652	6.4E-06	2.7E-03
**ENST00000377122**	ENSG00000078114	10	20779972	20897311	NEBL	protein_coding	Yes	NM_006393.3	-3.5492	-0.6278	19.9490	8.0E-06	3.0E-03
**ENST00000593581**	ENSG00000269386	19	8374371	8390685	RAB11B-AS1	lncRNA	Yes	-	1.0461	-0.9262	19.8322	8.5E-06	3.1E-03
**ENST00000349945**	ENSG00000102908	16	69565965	69704654	NFAT5	protein_coding	Yes	NM_138713.4	-1.0245	4.2586	19.6473	9.3E-06	3.2E-03
**ENST00000397195**	ENSG00000007168	17	2593653	2685615	PAFAH1B1	protein_coding	Yes	NM_000430.4	-1.0420	3.6363	19.6291	9.4E-06	3.3E-03
**ENST00000507317**	ENSG00000251215	5	39169209	39170335	GOLGA5P1	processed_pseudogene	Yes	-	-1.4056	0.4030	19.2438	1.2E-05	3.7E-03
**ENST00000602669**	ENSG00000269940	14	103694559	103695170	-	lncRNA	Yes	-	-1.6514	-1.3494	19.2302	1.2E-05	3.7E-03
**ENST00000700791**	ENSG00000289829	6	31826840	31827443	-	lncRNA	Yes	-	2.3507	-3.6404	19.1507	1.2E-05	3.7E-03
**ENST00000592908**	ENSG00000267074	17	35499689	35510270	-	lncRNA	Yes	-	-1.2673	0.1874	19.0254	1.3E-05	3.9E-03
**ENST00000351328**	ENSG00000062485	12	56271698	56300330	CS	protein_coding	Yes	NM_004077.3	-1.1081	1.9134	18.9457	1.3E-05	4.0E-03
**ENST00000262031**	ENSG00000076067	12	56521819	56596193	RBMS2	protein_coding	Yes	NM_002898.4	-1.2913	0.0836	18.8942	1.4E-05	4.0E-03
**ENST00000432992**	ENSG00000007237	17	9910605	10198606	GAS7	protein_coding	Yes	NM_201433.2	-1.0142	3.2747	18.7383	1.5E-05	4.2E-03
**ENST00000424573**	ENSG00000235174	6	73373107	73373263	RPL39P3	processed_pseudogene	Yes	-	1.0111	4.7511	18.6668	1.6E-05	4.3E-03

**ENST00000683076**	ENSG00000047849	3	47850694	48016416	MAP4	protein_coding	Yes	NM_001385682.1	-1.1282	2.1547	18.6580	1.6E-05	4.3E-03
**ENST00000624384**	ENSG00000280202	12	3610306	3612003	-	TEC	Yes	-	-1.3200	-1.2591	18.4890	1.7E-05	4.4E-03
**ENST00000702841**	ENSG00000289130	3	24137865	24294374	-	lncRNA	Yes	-	1.0649	1.2396	18.4304	1.8E-05	4.5E-03
**ENST00000582279**	ENSG00000266127	18	9785107	9787805	ZNF415P1	processed_pseudogene	Yes	-	-1.3157	-1.3362	18.3480	1.8E-05	4.6E-03
**ENST00000615164**	ENSG00000276966	6	26204609	26205021	H4C5	protein_coding	Yes	NM_003545.4	1.0798	3.9616	18.3354	1.9E-05	4.6E-03
**ENST00000410180**	ENSG00000222112	1	33336565	33336864	RN7SKP16	misc_RNA	Yes	-	-1.6489	-1.1021	18.0652	2.1E-05	4.8E-03
**ENST00000666926**	ENSG00000255197	11	47381508	47409271	-	lncRNA	Yes	-	-1.2866	1.0696	17.9974	2.2E-05	4.9E-03
**ENST00000560711**	ENSG00000259379	15	58152605	58154407	MTND5P32	processed_pseudogene	Yes	-	-1.8198	-1.9054	17.9219	2.3E-05	5.0E-03
**ENST00000615353**	ENSG00000276180	6	27139281	27139678	H4C9	protein_coding	Yes	NM_003495.3	1.0247	1.3723	17.9029	2.3E-05	5.0E-03
**ENST00000364711**	ENSG00000201581	10	6149622	6149940	RN7SKP78	misc_RNA	Yes	-	-3.7019	-4.6729	17.8183	2.4E-05	5.2E-03
**ENST00000612978**	ENSG00000278558	22	18527801	18530573	TMEM191B	protein_coding	Yes	NM_001242313.1	2.4328	-2.3274	17.7444	2.5E-05	5.3E-03
**ENST00000301908**	ENSG00000168081	8	28317267	28343351	PNOC	protein_coding	Yes	NM_006228.5	1.5042	-2.7815	17.5424	2.8E-05	5.5E-03
**ENST00000622996**	ENSG00000279432	17	2639296	2642418	-	TEC	Yes	-	-2.0247	-0.8225	17.4722	2.9E-05	5.6E-03
**ENST00000580341**	ENSG00000277452	1	150566561	150566860	RN7SL473P	misc_RNA	Yes	-	-1.6199	-0.9025	17.4391	3.0E-05	5.6E-03
**ENST00000614247**	ENSG00000277157	6	26188709	26189112	H4C4	protein_coding	Yes	NM_003539.4	1.3120	2.2667	17.3856	3.1E-05	5.7E-03
**ENST00000690187**	ENSG00000289557	12	104644172	104647370	-	lncRNA	Yes	-	-1.4436	-0.3181	17.3353	3.1E-05	5.8E-03
**ENST00000605249**	ENSG00000271204	7	130930208	130932206	-	lncRNA	Yes	-	-1.3179	1.4779	17.2724	3.2E-05	5.9E-03
**ENST00000301740**	ENSG00000167978	16	2752637	2771412	SRRM2	protein_coding	Yes	NM_016333.4	-1.3501	4.8347	17.1820	3.4E-05	6.0E-03
**ENST00000326586**	ENSG00000181524	6	42956344	42956765	RPL24P4	processed_pseudogene	Yes	-	1.0356	4.1990	17.0318	3.7E-05	6.2E-03
**ENST00000687578**	ENSG00000289176	2	64337102	64341709	-	lncRNA	Yes	-	1.5148	0.0236	16.9973	3.7E-05	6.2E-03
**ENST00000624428**	ENSG00000279884	2	174545384	174546335	-	TEC	Yes	-	-1.0508	0.4035	16.9601	3.8E-05	6.3E-03
**ENST00000441146**	ENSG00000229331	X	30671634	30672166	GK-IT1	lncRNA	Yes	-	-2.5963	-3.0643	16.8801	4.0E-05	6.3E-03
**ENST00000653887**	ENSG00000233559	7	130853731	130930680	LINC00513	lncRNA	Yes	-	-2.0213	-3.7632	16.8712	4.0E-05	6.3E-03
**ENST00000521025**	ENSG00000253200	8	22613907	22616657	-	lncRNA	Yes	-	-1.0139	-0.8130	16.8238	4.1E-05	6.3E-03
**ENST00000368655**	ENSG00000143614	1	153804724	153922972	GATAD2B	protein_coding	Yes	NM_020699.4	-1.2775	2.3467	16.7948	4.2E-05	6.4E-03

**ENST00000459853**	ENSG00000240233	14	77547565	77547846	RN7SL58 7P	misc_RNA	Yes	-	-2.2955	-3.4104	16.7816	4.2E-05	6.4E-03
**ENST00000428191**	ENSG00000236977	2	197250857	197302519	ANKRD44 -IT1	lncRNA	Yes	-	-1.2456	1.7788	16.6006	4.6E-05	6.7E-03
**ENST00000624840**	ENSG00000280205	18	76385641	76389406	-	TEC	Yes	-	-1.3568	-1.3048	16.5853	4.7E-05	6.7E-03
**ENST00000659311**	ENSG00000215244	10	6271168	6353827	LINC0264 9	lncRNA	Yes	-	-1.3526	-0.5618	16.5621	4.7E-05	6.7E-03
**ENST00000244537**	ENSG00000274618	6	26240392	26240793	H4C6	protein_coding	Yes	NM_003540.4	1.2233	2.9799	16.5519	4.7E-05	6.7E-03
**ENST00000653023**	ENSG00000235257	3	37693654	37861771	ITGA9- AS1	lncRNA	Yes	-	1.3033	-2.4121	16.5240	4.8E-05	6.8E-03
**ENST00000536718**	ENSG00000206140	22	21467386	21469935	TMEM191 C	protein_coding	Yes	NM_00138835 4.1	1.5708	-2.6056	16.5036	4.9E-05	6.8E-03
**ENST00000460535**	ENSG00000243544	3	121653993	121654296	RN7SL17 2P	misc_RNA	Yes	-	-1.3121	-1.7730	16.4774	4.9E-05	6.8E-03
**ENST00000674977**	ENSG00000181222	17	7484365	7514179	POLR2A	protein_coding	Yes	-	-1.3081	3.0882	16.4072	5.1E-05	6.9E-03
**ENST00000288050**	ENSG00000090857	16	70114331	70162537	PDPR	protein_coding	Yes	NM_017990.5	-1.2234	1.6385	16.3556	5.2E-05	7.0E-03
**ENST00000245458**	ENSG00000213741	14	49583576	49586380	RPS29	protein_coding	Yes	NM_001032.5	1.0410	4.8825	16.2796	5.5E-05	7.1E-03
**ENST00000604052**	ENSG00000270640	2	28396814	28397110	-	lncRNA	Yes	-	-1.4564	-2.5747	16.1599	5.8E-05	7.3E-03
**ENST00000303910**	ENSG00000277075	6	26216920	26217437	H2AC8	protein_coding	Yes	NM_021052.4	1.0012	2.7604	16.1353	5.9E-05	7.4E-03
**ENST00000434618**	ENSG00000231925	6	33299693	33314078	TAPBP	protein_coding	Yes	NM_003190.5	-1.0299	2.8830	16.1152	6.0E-05	7.4E-03
**ENST00000692099**	ENSG00000289465	11	109803904	110088173	-	lncRNA	Yes	-	1.0382	-1.7140	16.0368	6.2E-05	7.5E-03
**ENST00000374080**	ENSG00000184634	X	71118595	71142450	MED12	protein_coding	Yes	NM_005120.3	-1.1718	2.1080	16.0166	6.3E-05	7.5E-03
**ENST00000311111**	ENSG00000172809	17	74203677	74210655	RPL38	protein_coding	Yes	NM_000999.4	1.2085	6.0357	15.9867	6.4E-05	7.6E-03
**ENST00000631190**	ENSG00000280798	11	33076148	33079454	LINC0029 4	lncRNA	Yes	-	-1.2508	-0.7410	15.9833	6.4E-05	7.6E-03
**ENST00000301246**	ENSG00000167644	19	38304250	38305006	C19orf33	protein_coding	Yes	NM_033520.3	2.1199	-1.1189	15.9525	6.5E-05	7.6E-03
**ENST00000360270**	ENSG00000147065	X	65667653	65741931	MSN	protein_coding	Yes	NM_002444.3	-1.0108	5.1629	15.9666	6.4E-05	7.6E-03
**ENST00000490496**	ENSG00000240767	13	46169457	46169748	RN7SL28 8P	misc_RNA	Yes	-	-1.2452	-1.1521	15.9589	6.5E-05	7.6E-03
**ENST00000591899**	ENSG00000127540	19	1597168	1605462	UQCR11	protein_coding	Yes	NM_006830.4	1.2990	3.3183	15.9727	6.4E-05	7.6E-03
**ENST00000516685**	ENSG00000252494	11	78133419	78133522	RNU6- 126P	snRNA	Yes	-	-5.0060	-5.3847	15.9393	6.5E-05	7.6E-03
**ENST00000312966**	ENSG00000180221	10	58212540	58213036	TPT1P10	processed_ps eudogene	Yes	-	-1.3236	-2.0844	15.9179	6.6E-05	7.7E-03
**ENST00000390981**	ENSG00000212283	2	101272935	101273049	SNORD89	snoRNA	Yes	-	-1.1493	-0.2801	15.9076	6.7E-05	7.7E-03

**ENST00000648650**	ENSG00000285994	10	12563150	12567351	-	lncRNA	Yes	-	-1.6776	1.0753	15.8732	6.8E-05	7.7E-03
**ENST00000563751**	ENSG00000261332	16	30498765	30499554	-	lncRNA	Yes	-	-1.4535	-2.6305	15.8352	6.9E-05	7.8E-03
**ENST00000444853**	ENSG00000225195	12	64628343	64629976	-	lncRNA	Yes	-	-1.7243	-1.7858	15.7637	7.2E-05	8.0E-03
**ENST00000585937**	ENSG00000267059	19	1578338	1605445	-	protein_coding	Yes	-	1.2886	3.2628	15.7395	7.3E-05	8.0E-03
**ENST00000278407**	ENSG00000149131	11	57597684	57614848	SERPING 1	protein_coding	Yes	NM_000062.3	-1.3059	-1.7263	15.7003	7.4E-05	8.1E-03
**ENST00000009180**	ENSG00000010278	12	6200399	6238266	CD9	protein_coding	Yes	NM_001769.4	1.0748	2.1658	15.6415	7.7E-05	8.2E-03
**ENST00000560199**	ENSG00000259238	15	59688516	59689418	-	lncRNA	Yes	-	1.6204	-2.5443	15.6112	7.8E-05	8.3E-03
**ENST00000433182**	ENSG00000224707	6	20437820	20440178	E2F3-IT1	lncRNA	Yes	-	-1.5725	-1.8050	15.4944	8.3E-05	8.6E-03
**ENST00000458797**	ENSG00000238741	3	160514906	160515236	SCARNA7	scaRNA	Yes	-	1.2561	5.2288	15.4109	8.6E-05	8.8E-03
**ENST00000209875**	ENSG00000094916	12	54230941	54280122	CBX5	protein_coding	Yes	NM_012117.3	-1.0882	3.3408	15.4040	8.7E-05	8.8E-03
**ENST00000607928**	ENSG00000273456	2	202374768	202376142	-	lncRNA	Yes	-	1.3213	-2.5081	15.3857	8.8E-05	8.8E-03
**ENST00000427740**	ENSG00000227653	2	128518787	128519177	ISCA1P6	processed_ps eudogene	Yes	-	1.3903	3.2216	15.3658	8.9E-05	8.8E-03
**ENST00000558334**	ENSG00000259291	15	90074514	90082207	ZNF710-AS1	lncRNA	Yes	-	-1.2453	-2.1126	15.3607	8.9E-05	8.8E-03
**ENST00000441346**	ENSG00000234073	3	36880183	36880729	-	processed_ps eudogene	Yes	-	-1.0400	-0.9071	15.3354	9.0E-05	8.9E-03
**ENST00000685045**	ENSG00000289017	15	92908003	92909131	-	lncRNA	Yes	-	-1.2028	-0.6383	15.2203	9.6E-05	9.2E-03
**ENST00000657854**	ENSG00000245552	11	95151692	95234391	LNCRNA-IUR	lncRNA	Yes	-	1.3200	1.4292	15.1836	9.8E-05	9.2E-03
**ENST00000618305**	ENSG00000275126	6	27873147	27873534	H4C13	protein_coding	Yes	NM_003546.3	1.1639	0.9337	15.1671	9.8E-05	9.3E-03
**ENST00000363632**	ENSG00000200502	9	37160136	37160237	Y_RNA	misc_RNA	Yes	-	-1.3986	-3.0685	15.1137	1.0E-04	9.4E-03
**ENST00000372555**	ENSG00000101470	20	45823213	45827312	TNNC2	protein_coding	Yes	NM_003279.3	1.3777	-1.3272	15.0800	1.0E-04	9.5E-03
**ENST00000324288**	ENSG00000179583	16	10877201	10936394	CIITA	protein_coding	Yes	NM_000246.4	-1.0771	3.0232	14.9535	1.1E-04	9.8E-03
**ENST00000622989**	ENSG00000279330	16	3273608	3275807	-	TEC	Yes	-	-1.0863	-0.7385	14.9227	1.1E-04	9.9E-03
**ENST00000514568**	ENSG00000249790	12	8788273	8795789	LINC0297 2	lncRNA	Yes	-	2.2061	4.5496	14.8951	1.1E-04	9.9E-03
**ENST00000582634**	ENSG00000264653	8	130008333	130008453	MIR5194	miRNA	Yes	-	-1.6691	-2.0464	14.8447	1.2E-04	9.9E-03
**ENST00000623177**	ENSG00000278949	12	132865854	132867958	-	TEC	Yes	-	-2.1655	-1.4697	14.8595	1.2E-04	9.9E-03
**ENST00000377803**	ENSG00000197061	6	26103932	26104337	H4C3	protein_coding	Yes	NM_003542.4	1.0522	3.6973	14.8327	1.2E-04	1.0E-02
**ENST00000504686**	ENSG00000250254	4	37960397	37961128	PTTG2	protein_coding	Yes	NM_006607.3	-1.0690	-1.2408	14.8178	1.2E-04	1.0E-02

**ENST00000609432**	ENSG00000273243	22	45155538	45156011	-	lncRNA	Yes	-	-1.3218	-1.6190	14.8208	1.2E-04	1.0E-02
**ENST00000626977**	ENSG00000280543	8	130082737	130084768	ASAP1-IT2	lncRNA	Yes	-	-2.0845	-2.2949	14.8091	1.2E-04	1.0E-02
**ENST00000334018**	ENSG00000186998	22	29205895	29259597	EMID1	protein_coding	Yes	NM_133455.4	1.3627	-1.8370	14.7860	1.2E-04	1.0E-02
**ENST00000475661**	ENSG00000243704	17	1604373	1604672	RN7SL105P	misc_RNA	Yes	-	-1.6862	-2.7454	14.7673	1.2E-04	1.0E-02
**ENST00000634783**	ENSG00000283045	17	44486152	44486815	-	lncRNA	Yes	-	-2.1220	-3.2949	14.7302	1.2E-04	1.0E-02
**ENST00000258873**	ENSG00000103740	15	78167467	78234565	ACSBG1	protein_coding	Yes	NM_015162.5	1.0334	-1.0835	14.7179	1.2E-04	1.0E-02
**ENST00000582492**	ENSG00000263584	10	12578752	12578823	MIR4480	miRNA	Yes	-	-3.1542	-4.5854	14.7126	1.3E-04	1.0E-02
**ENST00000344423**	ENSG00000186918	8	28345589	28386460	ZNF395	protein_coding	Yes	NM_018660.3	-1.1877	0.5121	14.6986	1.3E-04	1.0E-02
**ENST00000330889**	ENSG00000184060	17	30921944	30959322	ADAP2	protein_coding	Yes	NM_018404.3	-1.0275	-0.2751	14.6933	1.3E-04	1.0E-02
**ENST00000568457**	ENSG00000260997	7	44958998	44960909	-	lncRNA	Yes	-	-1.2326	-1.1876	14.6822	1.3E-04	1.0E-02
**ENST00000333643**	ENSG00000184515	X	102153711	102155977	BEX5	protein_coding	Yes	NM_001012978.3	1.1499	-2.0453	14.6698	1.3E-04	1.0E-02
**ENST00000373789**	ENSG00000182010	10	62193085	62268844	RTKN2	protein_coding	Yes	NM_145307.4	-1.0769	1.0952	14.6623	1.3E-04	1.0E-02
**ENST00000293973**	ENSG00000162068	16	2471296	2474145	NTN3	protein_coding	Yes	NM_006181.3	3.8432	-2.7596	14.5828	1.3E-04	1.1E-02
**ENST00000533203**	ENSG00000255186	11	36386520	36388250	-	lncRNA	Yes	-	-2.5549	-4.1073	14.5347	1.4E-04	1.1E-02
**ENST00000460552**	ENSG00000242330	14	71448688	71448958	RN7SL683P	misc_RNA	Yes	-	-1.5380	-2.4982	14.5051	1.4E-04	1.1E-02
**ENST00000423539**	ENSG00000228079	2	64086352	64088246	-	lncRNA	Yes	-	-1.0492	-1.3811	14.4766	1.4E-04	1.1E-02
**ENST00000497468**	ENSG00000243260	4	39760999	39761298	RN7SL558P	misc_RNA	Yes	-	-2.4643	-3.6277	14.4398	1.4E-04	1.1E-02
**ENST00000582881**	ENSG00000265625	17	29644795	29645847	-	lncRNA	Yes	-	-1.1440	0.5899	14.4231	1.5E-04	1.1E-02
**ENST00000614811**	ENSG00000284184	16	15611029	15611095	MIR6506	miRNA	Yes	-	-1.1300	-1.9208	14.4219	1.5E-04	1.1E-02
**ENST00000689970**	ENSG00000223855	7	522572	530163	PDGFA-DT	lncRNA	Yes	-	1.3614	-0.4990	14.3809	1.5E-04	1.1E-02
**ENST00000689708**	ENSG00000288948	2	101797889	101800135	-	lncRNA	Yes	-	-1.4956	-1.3298	14.3249	1.5E-04	1.1E-02
**ENST00000268058**	ENSG00000140464	15	73994715	74047827	PML	protein_coding	Yes	NM_033238.3	-1.1227	0.2315	14.2988	1.6E-04	1.2E-02
**ENST00000429036**	ENSG00000125895	20	1180569	1184981	TMEM74B	protein_coding	Yes	NM_001304748.2	6.0814	-4.7024	14.1283	1.7E-04	1.2E-02
**ENST00000618107**	ENSG00000276089	13	50082294	50082484	-	misc_RNA	Yes	-	1.1407	-1.4018	14.1233	1.7E-04	1.2E-02
**ENST00000438127**	ENSG00000234268	11	117035796	117036051	-	processed_pseudogene	Yes	-	-1.3886	-1.9270	14.1142	1.7E-04	1.2E-02

**ENST00000510264**	ENSG00000290556	5	69607098	69624049	-	lncRNA	Yes	-	-1.0045	-0.9606	14.1155	1.7E-04	1.2E-02
**ENST00000493797**	ENSG00000239906	1	139789	140339	-	lncRNA	Yes	-	-2.1912	-0.9439	14.0933	1.7E-04	1.2E-02
**ENST00000413552**	ENSG00000234358	7	111971221	111971988	RPL7AP42	processed_pseudogene	Yes	-	-1.5400	-2.9345	14.0030	1.8E-04	1.2E-02
**ENST00000357045**	ENSG00000237655	2	177603088	177618572	-	lncRNA	Yes	-	5.1378	-5.0619	13.8844	1.9E-04	1.3E-02
**ENST00000376033**	ENSG00000204469	6	31620714	31637771	PRRC2A	protein_coding	Yes	NM_004638.4	-1.3077	2.2948	13.8605	2.0E-04	1.3E-02
**ENST00000686249**	ENSG00000289013	2	40394672	40396777	-	lncRNA	Yes	-	-1.4620	1.0716	13.8510	2.0E-04	1.3E-02
**ENST00000692682**	ENSG00000289082	2	42795325	42796750	-	lncRNA	Yes	-	-1.7106	-2.6125	13.8222	2.0E-04	1.3E-02
**ENST00000642557**	ENSG00000235169	1	3772748	3775956	SMIM1	protein_coding	Yes	NM_001288583.2	2.0651	-0.7597	13.8050	2.0E-04	1.3E-02
**ENST00000458235**	ENSG00000105639	19	17824781	17847982	JAK3	protein_coding	Yes	NM_000215.4	-1.0592	3.3975	13.7130	2.1E-04	1.3E-02
**ENST00000453262**	ENSG00000251578	7	142636923	142637384	TRBV21-1	TR_V_pseudogene	Yes	-	1.2382	-2.7697	13.7076	2.1E-04	1.3E-02
**ENST00000361066**	ENSG00000134202	1	109733936	109741038	GSTM3	protein_coding	Yes	NM_000849.5	1.8298	-0.8929	13.7027	2.1E-04	1.3E-02
**ENST00000478965**	ENSG00000243806	5	94825960	94826694	RPL7P18	processed_pseudogene	Yes	-	-1.1342	-1.8145	13.6624	2.2E-04	1.3E-02
**ENST00000452840**	ENSG00000233723	2	58428337	59063766	LINC01122	lncRNA	Yes	-	1.2103	-2.5011	13.6537	2.2E-04	1.3E-02
**ENST00000620884**	ENSG00000284067	7	44051765	44051829	MIR6837	miRNA	Yes	-	1.7627	0.9994	13.6296	2.2E-04	1.4E-02
**ENST00000318562**	ENSG00000177106	11	706230	727727	EPS8L2	protein_coding	Yes	NM_022772.4	1.1769	-0.9076	13.6096	2.3E-04	1.4E-02
**ENST00000439539**	ENSG00000240288	3	10285753	10293449	GHRLOS	lncRNA	Yes	-	-1.2835	-0.1175	13.5989	2.3E-04	1.4E-02
**ENST00000622688**	ENSG00000274964	12	32339367	32340724	-	lncRNA	Yes	-	-1.4660	-2.9321	13.5769	2.3E-04	1.4E-02
**ENST00000575953**	ENSG00000262514	16	68199794	68200981	-	lncRNA	Yes	-	-1.8922	-2.0122	13.5714	2.3E-04	1.4E-02
**ENST00000451143**	ENSG00000225900	2	39098148	39098445	HSPE1P13	processed_pseudogene	Yes	-	-1.9581	-2.8617	13.5467	2.3E-04	1.4E-02
**ENST00000262506**	ENSG00000070770	16	58157906	58198106	CSNK2A2	protein_coding	Yes	NM_001896.4	-1.1872	0.2450	13.4823	2.4E-04	1.4E-02
**ENST00000355057**	ENSG00000197238	6	27824091	27824480	H4C11	protein_coding	Yes	NM_021968.4	1.3270	0.3679	13.4314	2.5E-04	1.4E-02
**ENST00000378585**	ENSG00000187730	1	2019344	2030758	GABRD	protein_coding	Yes	NM_000815.5	4.0842	-3.2538	13.4156	2.5E-04	1.4E-02
**ENST00000580979**	ENSG00000264456	17	30971651	30973312	-	lncRNA	Yes	-	-1.6030	-2.5909	13.3904	2.5E-04	1.5E-02
**ENST00000365328**	ENSG00000202198	6	52995619	52995950	7SK	misc_RNA	Yes	-	1.2171	11.3532	13.3681	2.6E-04	1.5E-02
**ENST00000636484**	ENSG00000283293	6	52995620	52995948	RN7SK	snRNA	Yes	-	1.2171	11.3532	13.3681	2.6E-04	1.5E-02

**ENST00000513164**	ENSG00000248477	5	69618312	69623446	NAIPP3	transcribed_unprocessed_pseudogene	Yes	-	-1.1408	-1.3868	13.3522	2.6E-04	1.5E-02
**ENST00000358395**	ENSG00000101236	20	3927310	4015558	RNF24	protein_coding	Yes	NM_001134337.3	-1.0099	4.0102	13.3359	2.6E-04	1.5E-02
**ENST00000581881**	ENSG00000263642	4	40502039	40502119	MIR4802	miRNA	Yes	-	-2.4359	-3.7162	13.3338	2.6E-04	1.5E-02
**ENST00000473944**	ENSG00000232871	19	48680080	48681020	SEC1P	transcribed_unitary_pseudogene	Yes	-	4.4935	-4.2809	13.3317	2.6E-04	1.5E-02
**ENST00000274181**	ENSG00000145536	5	5140329	5320304	ADAMTS16	protein_coding	Yes	NM_139056.4	6.5610	-4.3886	13.3279	2.6E-04	1.5E-02
**ENST00000613826**	ENSG00000277687	10	118692360	118693535	-	lncRNA	Yes	-	-1.0053	-0.8264	13.3271	2.6E-04	1.5E-02
**ENST00000603520**	ENSG00000271461	20	1756755	1758940	CKAP2LP1	processed_pseudogene	Yes	-	-1.2520	-0.6331	13.2818	2.7E-04	1.5E-02
**ENST00000493687**	ENSG00000242256	9	112242549	112242839	RN7SL57P	misc_RNA	Yes	-	-2.0978	-3.2695	13.2683	2.7E-04	1.5E-02
**ENST00000606640**	ENSG00000272219	7	101960115	101961894	-	lncRNA	Yes	-	-1.7626	-2.1353	13.2403	2.7E-04	1.5E-02
**ENST00000425264**	ENSG00000225936	10	117238761	117241998	SLC18A2-AS1	lncRNA	Yes	-	1.0522	-0.0155	13.2327	2.8E-04	1.5E-02
**ENST00000542354**	ENSG00000255569	14	21621837	21622567	TRAV1-1	TR_V_gene	Yes	-	1.4969	-2.9649	13.1950	2.8E-04	1.5E-02
**ENST00000607434**	ENSG00000272356	6	111309202	111313517	-	lncRNA	Yes	-	-1.3079	-1.0316	13.1937	2.8E-04	1.5E-02
**ENST00000473748**	ENSG00000227097	11	82689558	82689768	RPS28P7	processed_pseudogene	Yes	-	1.3467	4.6633	13.1777	2.8E-04	1.5E-02
**ENST00000561147**	ENSG00000259378	15	50944662	50945996	DCAF13P3	processed_pseudogene	Yes	-	-1.1544	-2.2281	13.1407	2.9E-04	1.5E-02
**ENST00000491826**	ENSG00000229585	4	53986586	53987058	RPL21P44	processed_pseudogene	Yes	-	-1.3017	-1.9058	13.1269	2.9E-04	1.5E-02
**ENST00000280904**	ENSG00000134755	18	31058839	31102421	DSC2	protein_coding	Yes	NM_024422.6	-1.0423	2.9671	13.1110	2.9E-04	1.5E-02
**ENST00000581978**	ENSG00000266467	10	68007717	68008017	RN7SL220P	misc_RNA	Yes	-	-1.8577	-3.0317	13.1000	3.0E-04	1.5E-02
**ENST00000686452**	ENSG00000289102	19	52558016	52559570	-	lncRNA	Yes	-	-1.5917	-1.6230	13.0785	3.0E-04	1.5E-02
**ENST00000624440**	ENSG00000279235	15	78906126	78906809	-	TEC	Yes	-	-1.5389	-1.8448	13.0354	3.1E-04	1.6E-02
**ENST00000625059**	ENSG00000279679	5	143375627	143375883	-	TEC	Yes	-	-1.4273	-2.4160	13.0079	3.1E-04	1.6E-02
**ENST00000693095**	ENSG00000196890	1	228458102	228458558	H2BU1	protein_coding	Yes	NM_175055.3	1.1846	2.1625	12.9939	3.1E-04	1.6E-02
**ENST00000505325**	ENSG00000251682	5	61347125	61347716	-	processed_pseudogene	Yes	-	-1.0958	-0.6547	12.9889	3.1E-04	1.6E-02
**ENST00000243997**	ENSG00000124172	20	59025474	59032335	ATP5F1E	protein_coding	Yes	NM_006886.4	1.1161	5.3345	12.9859	3.1E-04	1.6E-02

**ENST00000691901**	ENSG00000145198	3	184229584	184242329	VWA5B2	protein_coding	Yes	NM_001390846.1	4.7210	-3.1887	12.9302	3.2E-04	1.6E-02
**ENST00000606908**	ENSG00000272040	5	75608816	75609983	-	lncRNA	Yes	-	-1.1535	-2.3992	12.9017	3.3E-04	1.6E-02
**ENST00000565768**	ENSG00000291105	16	56617422	56618832	-	lncRNA	Yes	-	2.5397	-2.0384	12.8795	3.3E-04	1.6E-02
**ENST00000360656**	ENSG00000197180	X	154424377	154428526	ATP6AP1-DT	lncRNA	Yes	-	1.4773	-3.1159	12.8736	3.3E-04	1.6E-02
**ENST00000624650**	ENSG00000279394	15	79875919	79878179	-	TEC	Yes	-	-1.4383	-1.1628	12.8637	3.4E-04	1.6E-02
**ENST00000529656**	ENSG00000254985	11	77717711	77718411	RSF1-IT2	lncRNA	Yes	-	-1.4155	-2.4957	12.8386	3.4E-04	1.6E-02
**ENST00000419899**	ENSG00000229325	3	195280722	195282741	ACAP2-IT1	lncRNA	Yes	-	-1.1128	-0.4456	12.8307	3.4E-04	1.6E-02
**ENST00000493028**	ENSG00000240531	17	29716278	29716685	RPL21P123	transcribed_processed_pseudogene	Yes	-	-1.3465	0.1512	12.8099	3.4E-04	1.6E-02
**ENST00000691244**	ENSG00000225127	20	21085577	21106358	LINC00237	lncRNA	Yes	-	4.9583	-5.1919	12.8039	3.5E-04	1.6E-02
**ENST00000400335**	ENSG00000183762	22	29073034	29146820	KREMEN1	protein_coding	Yes	NM_001039570.3	-1.1811	1.5346	12.7994	3.5E-04	1.6E-02
**ENST00000614007**	ENSG00000278757	1	516375	516479	U6	snRNA	Yes	-	-3.0890	-4.1780	12.7950	3.5E-04	1.7E-02
**ENST00000668204**	ENSG00000266401	18	3653029	3656269	-	lncRNA	Yes	-	1.0334	-0.8026	12.7697	3.5E-04	1.7E-02
**ENST00000647948**	ENSG00000262265	17	47160397	47167723	-	lncRNA	Yes	-	-1.2882	-1.3596	12.7624	3.5E-04	1.7E-02
**ENST00000684204**	ENSG00000288716	13	77326904	77326988	-	protein_coding	Yes	-	1.3696	-2.1806	12.7578	3.5E-04	1.7E-02
**ENST00000303746**	ENSG00000131650	16	2964274	2968380	KREMEN2	protein_coding	Yes	NM_172229.3	3.9542	-3.0735	12.7028	3.7E-04	1.7E-02
**ENST00000339180**	ENSG00000166091	14	23377045	23379772	CMTM5	protein_coding	Yes	NM_001288746.2	1.4336	0.5321	12.6914	3.7E-04	1.7E-02
**ENST00000355995**	ENSG00000148737	10	112950246	113167678	TCF7L2	protein_coding	Yes	NM_001367943.1	-1.0634	0.0304	12.6878	3.7E-04	1.7E-02
**ENST00000455103**	ENSG00000237711	9	5100235	5101009	MTCO3P11	unprocessed_pseudogene	Yes	-	-1.1307	-1.9262	12.6658	3.7E-04	1.7E-02
**ENST00000352861**	ENSG00000132432	7	54752252	54759211	SEC61G	protein_coding	Yes	NM_014302.4	1.0750	1.9058	12.6116	3.8E-04	1.7E-02
**ENST00000333323**	ENSG00000185614	3	49803260	49805030	INKA1	protein_coding	Yes	NM_203370.2	1.3759	-2.0130	12.5708	3.9E-04	1.8E-02
**ENST00000631644**	ENSG00000282508	19	209218	224007	LINC01002	lncRNA	Yes	-	-1.1316	-1.3270	12.5610	3.9E-04	1.8E-02
**ENST00000411999**	ENSG00000224442	2	197028710	197030143	RPL4P7	processed_pseudogene	Yes	-	-1.1438	-1.2560	12.5372	4.0E-04	1.8E-02
**ENST00000479087**	ENSG00000239419	7	143290614	143290910	RN7SL535P	misc_RNA	Yes	-	-2.5631	-4.3614	12.5195	4.0E-04	1.8E-02

**ENST00000370355**	ENSG00000099194	10	100347232	100364826	SCD	protein_coding	Yes	NM_005063.5	-1.3606	-0.6022	12.4778	4.1E-04	1.8E-02
**ENST00000390295**	ENSG00000211649	22	22369613	22370087	IGLV7-46	IG_V_gene	Yes	-	2.5229	-2.4556	12.4616	4.2E-04	1.8E-02
**ENST00000371175**	ENSG00000112077	6	49605174	49636839	RHAG	protein_coding	Yes	NM_000324.3	1.0755	1.5214	12.4570	4.2E-04	1.8E-02
**ENST00000692612**	ENSG00000289334	8	30094323	30095370	-	lncRNA	Yes	-	1.1919	-2.4046	12.4248	4.2E-04	1.8E-02
**ENST00000623975**	ENSG00000280181	12	64709457	64710513	-	TEC	Yes	-	-1.3069	-2.3252	12.4054	4.3E-04	1.9E-02
**ENST00000670694**	ENSG00000287680	18	77042863	77044739	-	lncRNA	Yes	-	-1.1991	-2.1825	12.3980	4.3E-04	1.9E-02
**ENST00000583426**	ENSG00000264546	17	62549725	62552121	-	lncRNA	Yes	-	-1.7364	-2.5260	12.3880	4.3E-04	1.9E-02
**ENST00000581296**	ENSG00000214425	17	45508108	45550335	LRRC37A4P	transcribed_unprocessed_pseudogene	Yes	-	-1.2366	2.8931	12.3823	4.3E-04	1.9E-02
**ENST00000501122**	ENSG00000245532	11	65422797	65445540	NEAT1	lncRNA	Yes	-	-1.0540	9.5619	12.3804	4.3E-04	1.9E-02
**ENST00000478664**	ENSG00000240589	17	44409058	44409358	RN7SL258P	misc_RNA	Yes	-	-1.9226	-3.3546	12.3752	4.4E-04	1.9E-02
**ENST00000395152**	ENSG00000213600	3	50239617	50239984	-	lncRNA	Yes	-	-1.6567	-2.1058	12.3684	4.4E-04	1.9E-02
**ENST00000363046**	ENSG00000277027	9	35657753	35658017	RMRP	ribozyme	Yes	-	1.1411	7.6521	12.3593	4.4E-04	1.9E-02
**ENST00000602361**	ENSG00000269900	9	35657750	35658018	RMRP	lncRNA	Yes	-	1.1404	7.6525	12.3460	4.4E-04	1.9E-02
**ENST00000606596**	ENSG00000272256	8	30082757	30083467	-	lncRNA	Yes	-	1.1023	1.5838	12.3419	4.4E-04	1.9E-02
**ENST00000366273**	ENSG00000213073	6	160093422	160093993	CHP1P2	transcribed_processed_pseudogene	Yes	-	-1.0242	-1.0491	12.3362	4.4E-04	1.9E-02
**ENST00000302057**	ENSG00000170561	5	2746164	2751677	IRX2	protein_coding	Yes	NM_033267.5	7.0706	-4.0195	12.3347	4.4E-04	1.9E-02
**ENST00000459460**	ENSG00000238829	2	201141903	201141966	RNU7-45P	snRNA	Yes	-	-1.6657	-2.4038	12.3054	4.5E-04	1.9E-02
**ENST00000308167**	ENSG00000102034	X	130063954	130110497	ELF4	protein_coding	Yes	NM_001421.4	-1.0439	2.0156	12.2868	4.6E-04	1.9E-02
**ENST00000533128**	ENSG00000254765	11	9958743	9959790	-	processed_pseudogene	Yes	-	-1.5750	-2.9750	12.2656	4.6E-04	1.9E-02
**ENST00000343958**	ENSG00000180263	12	95076748	95217467	FGD6	protein_coding	Yes	NM_018351.4	-1.0249	1.0653	12.2402	4.7E-04	1.9E-02
**ENST00000296029**	ENSG00000163737	4	73980810	73982027	PF4	protein_coding	Yes	NM_002619.4	1.6218	4.7556	12.2355	4.7E-04	1.9E-02
**ENST00000637617**	ENSG00000089159	12	120210446	120265730	PXN	protein_coding	Yes	NM_001385981.1	-1.1627	2.9266	12.2051	4.8E-04	2.0E-02
**ENST00000432323**	ENSG00000235363	1	205351246	205351471	SNRPGP10	processed_pseudogene	Yes	-	1.0604	-0.2469	12.2038	4.8E-04	2.0E-02
**ENST00000499986**	ENSG00000238035	5	181306501	181324130	-	lncRNA	Yes	-	-1.4390	-1.6371	12.1912	4.8E-04	2.0E-02

**ENST00000561241**	ENSG00000259498	15	63046033	63049387	TPM1-AS	lncRNA	Yes	-	1.4120	-1.1025	12.0870	5.1E-04	2.0E-02
**ENST00000624179**	ENSG00000279202	16	24098238	24099971	-	TEC	Yes	-	-1.4824	-1.4846	12.0720	5.1E-04	2.0E-02
**ENST00000665650**	ENSG00000227373	1	174110267	174159334	RABGAP1L-DT	lncRNA	Yes	-	1.2246	-2.2712	12.0134	5.3E-04	2.1E-02
**ENST00000602827**	ENSG00000270108	14	103687575	103688127	-	lncRNA	Yes	-	-1.2432	-0.9246	11.9935	5.3E-04	2.1E-02
**ENST00000390374**	ENSG00000211727	7	142492131	142492673	TRBV7-6	TR_V_gene	Yes	-	1.2497	-2.0576	11.9603	5.4E-04	2.1E-02
**ENST00000663947**	ENSG00000286314	5	65965372	65969143	-	lncRNA	Yes	-	-1.0757	0.4685	11.9152	5.6E-04	2.1E-02
**ENST00000364773**	ENSG00000201643	7	75943781	75943916	SNORA14A	snoRNA	Yes	-	-3.9887	-5.7463	11.9141	5.6E-04	2.1E-02
**ENST00000613359**	ENSG00000275215	21	8395606	8395759	RNA5-8SN3	rRNA	Yes	-	3.5446	-1.6942	11.8946	5.6E-04	2.1E-02
**ENST00000329267**	ENSG00000185198	19	685545	695452	PRSS57	protein_coding	Yes	NM_001308209.2	1.7827	-2.2766	11.8913	5.6E-04	2.1E-02
**ENST00000528520**	ENSG00000255097	11	9242447	9245509	-	lncRNA	Yes	-	-1.7916	-2.2839	11.8797	5.7E-04	2.1E-02
**ENST00000477922**	ENSG00000257743	7	142111717	142222324	MGAM2	protein_coding	Yes	NM_001293626.2	-1.1133	0.9101	11.8752	5.7E-04	2.1E-02
**ENST00000580909**	ENSG00000264350	18	54406888	54407114	SNRPGP2	processed_pseudogene	Yes	-	1.3271	1.2994	11.8728	5.7E-04	2.1E-02
**ENST00000687046**	ENSG00000288995	9	126819281	126820987	-	lncRNA	Yes	-	-1.8696	-2.7939	11.8733	5.7E-04	2.1E-02
**ENST00000264187**	ENSG00000116962	1	235975829	236065090	NID1	protein_coding	Yes	NM_002508.3	-1.0397	0.1699	11.8139	5.9E-04	2.2E-02
**ENST00000623719**	ENSG00000280069	17	30738181	30740275	-	TEC	Yes	-	-1.3547	-0.9295	11.7915	6.0E-04	2.2E-02
**ENST00000578186**	ENSG00000270882	1	149832656	149833052	H4C14	protein_coding	Yes	NM_003548.2	1.0563	3.9947	11.7845	6.0E-04	2.2E-02
**ENST00000504490**	ENSG00000251441	4	112356134	112359819	RTEL1P1	transcribed_processed_pseudogene	Yes	-	-1.4061	-1.0051	11.7728	6.0E-04	2.2E-02
**ENST00000330676**	ENSG00000185561	17	1702815	1710377	TLCD2	protein_coding	Yes	NM_001164407.2	1.4562	-1.6922	11.7578	6.1E-04	2.2E-02
**ENST00000358894**	ENSG00000101203	20	63293185	63334806	COL20A1	protein_coding	Yes	NM_020882.4	5.7105	-3.3749	11.7580	6.1E-04	2.2E-02
**ENST00000326577**	ENSG00000006327	16	3020367	3022383	TNFRSF12A	protein_coding	Yes	NM_016639.3	1.1963	0.0702	11.6984	6.3E-04	2.2E-02
**ENST00000393597**	ENSG00000175591	11	73218280	73242427	P2RY2	protein_coding	Yes	NM_002564.4	-1.1886	0.5525	11.6442	6.4E-04	2.3E-02
**ENST00000559334**	ENSG00000259600	15	58151032	58152513	-	processed_pseudogene	Yes	-	-1.2507	-1.7226	11.6311	6.5E-04	2.3E-02
**ENST00000566367**	ENSG00000260549	16	56617562	56618680	MT1L	transcribed_unprocessed_pseudogene	Yes	-	2.6516	-2.1429	11.6284	6.5E-04	2.3E-02

**ENST00000682224**	ENSG00000288719	22	42183826	42207600	-	lncRNA	Yes	-	-1.2154	-0.6629	11.6043	6.6E-04	2.3E-02
**ENST00000693616**	ENSG00000289514	8	41797776	41799172	-	lncRNA	Yes	-	1.2315	0.5271	11.5753	6.7E-04	2.3E-02
**ENST00000381568**	ENSG00000284554	22	39014362	39033276	-	protein_coding	Yes	-	-1.2656	-0.9240	11.5664	6.7E-04	2.3E-02
**ENST00000233948**	ENSG00000115596	2	218859804	218874233	WNT6	protein_coding	Yes	NM_006522.4	4.6092	-3.6599	11.5652	6.7E-04	2.3E-02
**ENST00000266735**	ENSG00000139343	12	95858951	95866140	SNRPF	protein_coding	Yes	NM_003095.5	1.0520	1.3151	11.5003	7.0E-04	2.4E-02
**ENST00000517330**	ENSG00000253558	5	78041887	78044136	-	transcribed_processed_pseudogene	Yes	-	-1.1564	-1.1664	11.4605	7.1E-04	2.4E-02
**ENST00000623398**	ENSG00000279294	16	81618504	81620563	-	TEC	Yes	-	-1.1106	-2.2629	11.4098	7.3E-04	2.4E-02
**ENST00000565469**	ENSG00000260286	6	24797334	24798917	ARMH2	protein_coding	Yes	NM_001282492.2	-1.1336	-1.8735	11.4090	7.3E-04	2.4E-02
**ENST00000399581**	ENSG00000278530	X	80272064	80335227	CHMP1B2P	transcribed_unitary_pseudogene	Yes	-	2.1471	-2.8179	11.4033	7.3E-04	2.4E-02
**ENST00000362860**	ENSG00000199730	15	58703851	58704154	RN7SKP95	misc_RNA	Yes	-	-1.8165	-3.2012	11.3844	7.4E-04	2.4E-02
**ENST00000322630**	ENSG00000178404	17	78890578	78903201	CEP295NL	protein_coding	Yes	NM_001243540.2	-1.5399	-0.7900	11.3830	7.4E-04	2.4E-02
**ENST00000622824**	ENSG00000198488	11	77034397	77041973	B3GNT6	protein_coding	Yes	NM_138706.5	6.6304	-4.3611	11.3767	7.4E-04	2.5E-02
**ENST00000456273**	ENSG00000224975	X	47204920	47205865	INE1	lncRNA	Yes	-	-1.0233	-1.7889	11.3598	7.5E-04	2.5E-02
**ENST00000293255**	ENSG00000105507	19	48029382	48044079	CABP5	protein_coding	Yes	NM_019855.5	1.1499	-0.4012	11.3419	7.6E-04	2.5E-02
**ENST00000393264**	ENSG00000213279	22	49572263	49575426	-	lncRNA	Yes	-	-2.5637	-2.2265	11.3322	7.6E-04	2.5E-02
**ENST00000370859**	ENSG00000137968	1	75202128	75611114	SLC44A5	protein_coding	Yes	NM_001130058.2	2.5180	-2.2496	11.3283	7.6E-04	2.5E-02
**ENST00000581917**	ENSG00000264895	17	51249578	51251748	-	lncRNA	Yes	-	-1.0218	-0.2500	11.2944	7.8E-04	2.5E-02
**ENST00000407683**	ENSG00000204099	2	241809192	241817413	NEU4	protein_coding	Yes	NM_001167600.3	7.6144	-3.5456	11.2872	7.8E-04	2.5E-02
**ENST00000611923**	ENSG00000278713	16	29862759	29863417	-	lncRNA	Yes	-	1.0238	-0.8665	11.2748	7.9E-04	2.5E-02
**ENST00000623742**	ENSG00000279425	19	24078480	24080669	-	TEC	Yes	-	-1.0245	-1.2578	11.2661	7.9E-04	2.5E-02
**ENST00000591533**	ENSG00000267124	19	305572	306467	-	lncRNA	Yes	-	6.3246	-4.6126	11.2524	8.0E-04	2.6E-02
**ENST00000407595**	ENSG00000055813	2	56183989	56386172	CCDC85A	protein_coding	Yes	NM_001080433.2	3.6592	-3.1867	11.2441	8.0E-04	2.6E-02
**ENST00000607344**	ENSG00000272469	8	73833772	73834042	RN7SL760P	misc_RNA	Yes	-	-1.6942	-2.6095	11.2393	8.0E-04	2.6E-02

**ENST00000263735**	ENSG00000119888	2	47369310	47387020	EPCAM	protein_coding	Yes	NM_002354.3	1.0240	-0.9395	11.2257	8.1E-04	2.6E-02
**ENST00000608450**	ENSG00000272768	7	44884952	44886393	-	lncRNA	Yes	-	1.1619	-2.3936	11.2066	8.2E-04	2.6E-02
**ENST00000530158**	ENSG00000254916	11	90051836	90053736	-	unprocessed_pseudogene	Yes	-	6.8291	-4.1722	11.2059	8.2E-04	2.6E-02
**ENST00000451496**	ENSG00000229666	5	67001382	67004080	MAST4-AS1	lncRNA	Yes	-	1.4674	-2.5296	11.1814	8.3E-04	2.6E-02
**ENST00000565433**	ENSG00000260401	11	73238974	73242335	-	lncRNA	Yes	-	-1.1494	-0.9065	11.1788	8.3E-04	2.6E-02
**ENST00000590469**	ENSG00000115266	19	1450120	1473244	APC2	protein_coding	Yes	NM_005883.3	3.1381	-0.7994	11.1330	8.5E-04	2.7E-02
**ENST00000439099**	ENSG00000227383	9	34191554	34191881	RPL35AP2	processed_pseudogene	Yes	-	-1.7514	-3.1193	11.1197	8.5E-04	2.7E-02
**ENST00000651898**	ENSG00000260625	16	31508463	31509560	RUSF1-DT	lncRNA	Yes	-	1.3648	-3.0417	11.1108	8.6E-04	2.7E-02
**ENST00000263610**	ENSG00000125492	9	132582605	132590252	BARHL1	protein_coding	Yes	NM_020064.4	6.8561	-4.2185	11.1039	8.6E-04	2.7E-02
**ENST00000410926**	ENSG00000222858	17	29641610	29641702	RNU6-920P	snRNA	Yes	-	-1.5140	-2.6616	11.1010	8.6E-04	2.7E-02
**ENST00000623116**	ENSG00000279544	2	32563327	32566436	-	TEC	Yes	-	-1.3546	-0.7596	11.0888	8.7E-04	2.7E-02
**ENST00000547580**	ENSG00000257169	12	96386817	96387194	-	processed_pseudogene	Yes	-	-1.6964	-2.3495	11.0862	8.7E-04	2.7E-02
**ENST00000601752**	ENSG00000268051	19	37963852	37964790	-	lncRNA	Yes	-	-2.6031	-3.9794	11.0603	8.8E-04	2.7E-02
**ENST00000654339**	ENSG00000287195	8	129953728	129964634	-	lncRNA	Yes	-	-1.8060	-3.0746	11.0389	8.9E-04	2.7E-02
**ENST00000384638**	ENSG00000207368	3	195419435	195419546	Y_RNA	misc_RNA	Yes	-	-1.5887	-3.3941	11.0344	8.9E-04	2.7E-02
**ENST00000264246**	ENSG00000121594	3	119524292	119559614	CD80	protein_coding	Yes	NM_005191.4	-1.1650	-1.9679	11.0321	9.0E-04	2.7E-02
**ENST00000614819**	ENSG00000275807	16	28822430	28823969	-	lncRNA	Yes	-	1.1331	-1.8701	11.0299	9.0E-04	2.7E-02
**ENST00000559068**	ENSG00000259626	15	58150634	58150962	MTND3P12	processed_pseudogene	Yes	-	-1.0724	-2.4795	11.0246	9.0E-04	2.7E-02
**ENST00000616182**	ENSG00000274641	6	27893424	27893891	H2BC17	protein_coding	Yes	NM_003527.4	1.1881	0.1996	11.0209	9.0E-04	2.7E-02
**ENST00000569722**	ENSG00000267127	18	80034345	80097088	-	protein_coding	Yes	-	1.3608	-2.2014	10.9624	9.3E-04	2.8E-02
**ENST00000468439**	ENSG00000240890	3	132386521	132389074	-	processed_pseudogene	Yes	-	-1.5542	-2.4312	10.9536	9.3E-04	2.8E-02
**ENST00000420161**	ENSG00000243179	2	113979908	113983258	-	lncRNA	Yes	-	-1.1038	-2.3602	10.9423	9.4E-04	2.8E-02
**ENST00000658492**	ENSG00000259070	14	38747957	38948243	LINC00639	lncRNA	Yes	-	1.5080	-1.3236	10.9420	9.4E-04	2.8E-02
**ENST00000624567**	ENSG00000279957	2	113904085	113905258	-	TEC	Yes	-	-1.5294	-2.5344	10.9371	9.4E-04	2.8E-02

**ENST00000455617**	ENSG00000229492	22	17418696	17419828	-	processed_pseudogene	Yes	-	6.2489	-4.6257	10.9352	9.4E-04	2.8E-02
**ENST00000567091**	ENSG00000260394	16	678503	679777	STUB1-DT	lncRNA	Yes	-	5.2726	-5.1835	10.9217	9.5E-04	2.8E-02
**ENST00000412472**	ENSG00000206066	22	25318255	25320080	IGLL3P	unprocessed_pseudogene	Yes	-	-5.1958	-4.2317	10.9205	9.5E-04	2.8E-02
**ENST00000689966**	ENSG00000289391	11	121418873	121419179	-	lncRNA	Yes	-	1.7805	-2.6841	10.9155	9.5E-04	2.8E-02
**ENST00000622683**	ENSG00000275832	17	38428463	38512385	ARHGAP23	protein_coding	Yes	NM_001199417.2	2.5455	-2.3197	10.9124	9.6E-04	2.8E-02
**ENST00000607572**	ENSG00000271989	1	10429880	10430677	-	lncRNA	Yes	-	3.5232	-4.1061	10.8973	9.6E-04	2.8E-02
**ENST00000568149**	ENSG00000260954	16	1579241	1580308	-	lncRNA	Yes	-	-4.1199	-5.7080	10.8937	9.6E-04	2.9E-02
**ENST00000518507**	ENSG00000253475	8	51895956	51896374	-	lncRNA	Yes	-	-1.6566	-2.4983	10.8904	9.7E-04	2.9E-02
**ENST00000624936**	ENSG00000280136	17	88640	118578	-	lncRNA	Yes	-	-1.1051	-0.7957	10.8784	9.7E-04	2.9E-02
**ENST00000631455**	ENSG00000282100	15	58691105	58693125	HSP90AB4P	processed_pseudogene	Yes	-	-1.3865	-0.8737	10.8446	9.9E-04	2.9E-02
**ENST00000637683**	ENSG00000255363	11	76607852	76630427	LINC02757	lncRNA	Yes	-	1.4378	-3.1783	10.8180	1.0E-03	2.9E-02
**ENST00000670966**	ENSG00000287878	3	9693181	9721399	-	lncRNA	Yes	-	-1.7673	-3.8368	10.8110	1.0E-03	2.9E-02
**ENST00000468074**	ENSG00000240695	3	136736499	136737229	-	processed_pseudogene	Yes	-	-1.0446	-1.5320	10.7963	1.0E-03	2.9E-02
**ENST00000621730**	ENSG00000274776	18	46786698	46789297	-	lncRNA	Yes	-	-1.1066	-3.0363	10.7850	1.0E-03	2.9E-02
**ENST00000245185**	ENSG00000125148	16	56608583	56609497	MT2A	protein_coding	Yes	NM_005953.5	1.2187	1.1587	10.7818	1.0E-03	2.9E-02
**ENST00000465940**	ENSG00000243175	4	143425051	143425920	RPSAP36	transcribed_processed_pseudogene	Yes	-	-1.2435	-2.4230	10.7792	1.0E-03	2.9E-02
**ENST00000374840**	ENSG00000162551	1	21509422	21578410	ALPL	protein_coding	Yes	NM_000478.6	-1.0572	2.5605	10.7773	1.0E-03	2.9E-02
**ENST00000561363**	ENSG00000259651	15	58150274	58150563	MTCO3P23	processed_pseudogene	Yes	-	-1.0258	-1.5156	10.7668	1.0E-03	3.0E-02
**ENST00000262768**	ENSG00000035862	17	78852976	78925387	TIMP2	protein_coding	Yes	NM_003255.5	-1.0177	1.9290	10.7483	1.0E-03	3.0E-02
**ENST00000364473**	ENSG00000201343	3	177120690	177120800	Y_RNA	misc_RNA	Yes	-	-1.2952	-2.4813	10.7382	1.0E-03	3.0E-02
**ENST00000262776**	ENSG00000108679	17	78971254	78979923	LGALS3BP	protein_coding	Yes	NM_005567.4	-1.0797	-1.4685	10.7096	1.1E-03	3.0E-02
**ENST00000484900**	ENSG00000242182	18	23004563	23004862	RN7SL745P	misc_RNA	Yes	-	-4.1343	-5.7058	10.7031	1.1E-03	3.0E-02
**ENST00000284523**	ENSG00000154342	1	228006997	228061271	WNT3A	protein_coding	Yes	NM_033131.4	5.6542	-4.0204	10.7006	1.1E-03	3.0E-02

**ENST00000585369**	ENSG00000267263	17	77469067	77471045	-	lncRNA	Yes	-	-1.9734	-2.8018	10.6889	1.1E-03	3.0E-02
**ENST00000602658**	ENSG00000227888	8	12362381	12388296	FAM66A	lncRNA	Yes	-	2.0566	-4.2964	10.6743	1.1E-03	3.0E-02
**ENST00000617574**	ENSG00000274093	16	69632140	69632571	-	lncRNA	Yes	-	-1.5490	-1.9763	10.6603	1.1E-03	3.0E-02
**ENST00000638795**	ENSG00000284391	X	70427449	70435350	-	lncRNA	Yes	-	4.9769	-5.2955	10.6595	1.1E-03	3.0E-02
**ENST00000401216**	ENSG00000216035	10	29602263	29602346	MIR938	miRNA	Yes	-	-2.5076	-4.2062	10.6464	1.1E-03	3.0E-02
**ENST00000319653**	ENSG00000155816	1	240091882	240475187	FMN2	protein_coding	Yes	NM_020066.5	5.7911	-3.9657	10.6428	1.1E-03	3.1E-02
**ENST00000671267**	ENSG00000229980	17	50866753	50910774	TOB1-AS1	lncRNA	Yes	-	1.0108	-2.1445	10.6378	1.1E-03	3.1E-02
**ENST00000517099**	ENSG00000252908	5	17240610	17240715	RNU6-1003P	snRNA	Yes	-	-1.5087	-3.3470	10.6304	1.1E-03	3.1E-02
**ENST00000592032**	ENSG00000267225	18	57027831	57038845	WDR7-OT1	lncRNA	Yes	-	-1.0289	-1.5073	10.6224	1.1E-03	3.1E-02
**ENST00000516413**	ENSG00000252222	1	184821427	184821489	RNU7-13P	snRNA	Yes	-	-1.4306	-3.4988	10.6103	1.1E-03	3.1E-02
**ENST00000358078**	ENSG00000072182	2	219514481	219538772	ASIC4	protein_coding	Yes	NM_018674.6	5.6154	-4.9986	10.6060	1.1E-03	3.1E-02
**ENST00000623054**	ENSG00000279281	17	57448217	57450291	-	TEC	Yes	-	-1.4374	-2.0705	10.6041	1.1E-03	3.1E-02
**ENST00000688886**	ENSG00000289006	13	25096139	25098229	-	lncRNA	Yes	-	-1.2480	-1.1465	10.5868	1.1E-03	3.1E-02
**ENST00000505635**	ENSG00000251158	5	69898866	69903198	-	unprocessed_pseudogene	Yes	-	-1.7233	-1.9864	10.5659	1.2E-03	3.1E-02
**ENST00000402856**	ENSG00000219409	6	144397958	144398752	-	processed_pseudogene	Yes	-	-1.1706	-2.3691	10.5586	1.2E-03	3.1E-02
**ENST00000308874**	ENSG00000172889	9	136662915	136672678	EGFL7	protein_coding	Yes	NM_016215.5	1.3603	-2.0874	10.5556	1.2E-03	3.1E-02
**ENST00000310581**	ENSG00000164362	5	1253166	1295068	TERT	protein_coding	Yes	NM_198253.3	4.0599	-2.6248	10.5496	1.2E-03	3.1E-02
**ENST00000389561**	ENSG00000183495	12	131949941	132080460	EP400	protein_coding	Yes	NM_015409.5	-1.0640	2.4301	10.5297	1.2E-03	3.1E-02
**ENST00000624600**	ENSG00000279434	14	35086034	35088059	-	TEC	Yes	-	-1.6741	-2.8929	10.5042	1.2E-03	3.2E-02
**ENST00000607611**	ENSG00000272033	1	167379107	167381000	-	lncRNA	Yes	-	-1.2028	-1.7501	10.4842	1.2E-03	3.2E-02
**ENST00000493458**	ENSG00000239763	16	74312608	74313390	-	processed_pseudogene	Yes	-	-1.5735	-2.6527	10.4825	1.2E-03	3.2E-02
**ENST00000431297**	ENSG00000229231	21	13762337	13764332	FEM1AP1	processed_pseudogene	Yes	-	5.7236	-4.9657	10.4788	1.2E-03	3.2E-02
**ENST00000608444**	ENSG00000272599	10	73124572	73125405	-	lncRNA	Yes	-	-1.1557	-1.8991	10.4711	1.2E-03	3.2E-02
**ENST00000372583**	ENSG00000127124	1	41506364	41918922	HIVEP3	protein_coding	Yes	NM_024503.5	-1.1903	-0.1671	10.4652	1.2E-03	3.2E-02

**ENST00000566796**	ENSG00000261056	16	53298223	53299792	-	transcribed_processed_pseudogene	Yes	-	-1.1196	-2.1817	10.4641	1.2E-03	3.2E-02
**ENST00000584701**	ENSG00000265203	10	47348362	47357881	RBP3	protein_coding	Yes	NM_002900.3	5.1385	-5.1976	10.4473	1.2E-03	3.2E-02
**ENST00000617563**	ENSG00000275580	15	49343607	49344254	-	lncRNA	Yes	-	-1.2082	-2.8463	10.4404	1.2E-03	3.2E-02
**ENST00000703003**	ENSG00000289251	7	45187004	45192605	-	lncRNA	Yes	-	-1.4074	-3.1465	10.4370	1.2E-03	3.2E-02
**ENST00000507617**	ENSG00000248785	4	109673842	109674124	HIGD1AP14	processed_pseudogene	Yes	-	-1.4013	-2.7470	10.4278	1.2E-03	3.2E-02
**ENST00000204604**	ENSG00000090539	3	184380053	184389829	CHRD	protein_coding	Yes	NM_003741.4	6.9309	-4.1435	10.4246	1.2E-03	3.2E-02
**ENST00000526902**	ENSG00000255358	11	130285022	130285833	NDUFAF2P2	processed_pseudogene	Yes	-	-1.0812	-2.2198	10.4154	1.2E-03	3.2E-02
**ENST00000446590**	ENSG00000234174	2	113171534	113175337	-	lncRNA	Yes	-	4.1612	-5.4672	10.4069	1.3E-03	3.3E-02
**ENST00000623278**	ENSG00000279741	16	53389885	53390563	-	TEC	Yes	-	-1.2335	-1.7998	10.3937	1.3E-03	3.3E-02
**ENST00000702521**	ENSG00000290022	4	139841167	139841542	-	lncRNA	Yes	-	-1.5947	-3.1588	10.3898	1.3E-03	3.3E-02
**ENST00000381480**	ENSG00000205744	19	6467206	6481787	DENND1C	protein_coding	Yes	NM_024898.4	-1.0461	0.0431	10.3618	1.3E-03	3.3E-02
**ENST00000445059**	ENSG00000236375	10	68010204	68010862	POU5F1P5	processed_pseudogene	Yes	-	-1.0493	-1.6403	10.3580	1.3E-03	3.3E-02
**ENST00000567563**	ENSG00000259827	16	56682469	56687807	-	lncRNA	Yes	-	1.0314	-0.1373	10.3527	1.3E-03	3.3E-02
**ENST00000392607**	ENSG00000197177	10	133087923	133131675	ADGRA1	protein_coding	Yes	NM_001083909.3	5.8326	-3.2541	10.3420	1.3E-03	3.3E-02
**ENST00000454743**	ENSG00000227536	X	71043213	71045470	SOCS5P4	processed_pseudogene	Yes	-	-1.5299	-2.6957	10.3422	1.3E-03	3.3E-02
**ENST00000304672**	ENSG00000171611	6	42916052	42925838	PTCRA	protein_coding	Yes	NM_138296.3	1.0550	0.0011	10.3123	1.3E-03	3.3E-02
**ENST00000529166**	ENSG00000254852	16	14742532	14765462	NPIPA2	protein_coding	Yes	NM_001395485.2	1.0105	-1.8375	10.2863	1.3E-03	3.4E-02
**ENST00000702113**	ENSG00000289180	14	69069307	69105927	-	lncRNA	Yes	-	1.2585	-2.2225	10.2767	1.3E-03	3.4E-02
**ENST00000624147**	ENSG00000279133	17	62003699	62007518	-	TEC	Yes	-	-1.1737	-0.8077	10.2491	1.4E-03	3.4E-02
**ENST00000240328**	ENSG00000121068	17	61399842	61409466	TBX2	protein_coding	Yes	NM_005994.4	5.1362	-3.8317	10.2393	1.4E-03	3.4E-02
**ENST00000429843**	ENSG00000234293	21	29496046	29500386	BACH1-IT3	lncRNA	Yes	-	-1.1852	-2.6059	10.2300	1.4E-03	3.4E-02
**ENST00000439958**	ENSG00000149532	11	61402728	61429975	CPSF7	protein_coding	Yes	NM_001142565.3	-1.0084	1.3760	10.2293	1.4E-03	3.4E-02
**ENST00000597582**	ENSG00000152475	19	58367622	58381030	ZNF837	protein_coding	Yes	NM_138466.2	1.4827	-1.3583	10.2269	1.4E-03	3.4E-02
**ENST00000572103**	ENSG00000262803	6	70693838	70694619	-	processed_pseudogene	Yes	-	-1.4130	-2.5018	10.2260	1.4E-03	3.4E-02

**ENST00000621102**	ENSG00000274954	15	51833133	51833426	-	lncRNA	Yes	-	-2.3948	-4.4158	10.1996	1.4E-03	3.5E-02
**ENST00000606361**	ENSG00000272037	8	102256391	102257821	-	lncRNA	Yes	-	-1.0349	-1.9192	10.1965	1.4E-03	3.5E-02
**ENST00000384307**	ENSG00000207034	18	2778940	2779057	Y_RNA	misc_RNA	Yes	-	-1.0043	-1.0142	10.1932	1.4E-03	3.5E-02
**ENST00000458392**	ENSG00000232300	17	46558829	46562795	FAM215B	lncRNA	Yes	-	-1.6298	-1.2164	10.1855	1.4E-03	3.5E-02
**ENST00000411054**	ENSG00000222986	1	231670634	231670750	RNU5A-5P	snRNA	Yes	-	-3.9205	-5.7539	10.1772	1.4E-03	3.5E-02
**ENST00000610217**	ENSG00000273353	22	45178989	45179310	-	lncRNA	Yes	-	-1.3958	-3.3851	10.1775	1.4E-03	3.5E-02
**ENST00000696174**	ENSG00000188897	16	11372014	11527247	-	protein_coding	Yes	NM_001370704.1	-1.1522	2.8796	10.1620	1.4E-03	3.5E-02
**ENST00000417001**	ENSG00000233765	9	66253423	66254464	CDRT15P12	unprocessed_pseudogene	Yes	-	4.4372	-5.4216	10.1601	1.4E-03	3.5E-02
**ENST00000338033**	ENSG00000181085	8	143716348	143722458	MAPK15	protein_coding	Yes	NM_139021.3	6.9928	-4.1110	10.1579	1.4E-03	3.5E-02
**ENST00000510714**	ENSG00000188242	5	466123	472876	PP7080	lncRNA	Yes	-	1.6165	-2.9319	10.1561	1.4E-03	3.5E-02
**ENST00000565390**	ENSG00000260920	1	40464318	40466767	-	lncRNA	Yes	-	-1.2243	-2.8246	10.1479	1.4E-03	3.5E-02
**ENST00000382723**	ENSG00000163132	4	4859664	4863936	MSX1	protein_coding	Yes	NM_002448.3	6.5848	-4.3857	10.1290	1.5E-03	3.5E-02
**ENST00000397770**	ENSG00000076344	16	268306	275944	RGS11	protein_coding	Yes	NM_183337.3	5.6835	-3.3570	10.1258	1.5E-03	3.5E-02
**ENST00000652939**	ENSG00000248455	5	17403960	17442542	LINC02217	lncRNA	Yes	-	1.6582	-3.7587	10.1190	1.5E-03	3.5E-02
**ENST00000612531**	ENSG00000273824	12	68426330	68427737	-	lncRNA	Yes	-	-1.9236	-1.2655	10.1162	1.5E-03	3.5E-02
**ENST00000414890**	ENSG00000223525	1	174896957	174897996	RABGAP1L-IT1	lncRNA	Yes	-	-1.6754	-3.0199	10.1029	1.5E-03	3.5E-02
**ENST00000162330**	ENSG00000050820	16	75228180	75251624	BCAR1	protein_coding	Yes	NM_014567.5	5.4115	-3.6535	10.0933	1.5E-03	3.5E-02
**ENST00000585212**	ENSG00000265791	17	30781492	30782221	-	lncRNA	Yes	-	-1.3659	-2.7597	10.0824	1.5E-03	3.6E-02
**ENST00000623342**	ENSG00000279078	7	127997596	128000077	SND1-IT1	lncRNA	Yes	-	-1.2930	-1.7264	10.0788	1.5E-03	3.6E-02
**ENST00000435074**	ENSG00000291111	6	33112439	33119163	-	lncRNA	Yes	-	1.6046	-3.5139	10.0645	1.5E-03	3.6E-02
**ENST00000365280**	ENSG00000202150	20	35030316	35030420	RNU6-407P	snRNA	Yes	-	-1.9278	-4.1357	10.0483	1.5E-03	3.6E-02
**ENST00000606601**	ENSG00000283736	16	15643293	15643372	MIR484	miRNA	Yes	-	4.0561	-5.5760	10.0403	1.5E-03	3.6E-02
**ENST00000584643**	ENSG00000266538	17	15530772	15531089	-	lncRNA	Yes	-	-1.5977	-2.6365	10.0365	1.5E-03	3.6E-02
**ENST00000693241**	ENSG00000289548	20	62533956	62536728	-	lncRNA	Yes	-	1.5879	-2.2338	10.0332	1.5E-03	3.6E-02
**ENST00000541888**	ENSG00000256433	12	6393904	6396148	-	lncRNA	Yes	-	-1.1513	-2.7836	10.0291	1.5E-03	3.6E-02

**ENST00000592400**	ENSG00000267735	19	12880968	12884088	-	lncRNA	Yes	-	1.2397	-2.4946	10.0196	1.5E-03	3.6E-02
**ENST00000257572**	ENSG00000135116	12	116856143	116881441	HRK	protein_coding	Yes	NM_003806.4	1.3909	-2.2917	10.0153	1.6E-03	3.6E-02
**ENST00000244241**	ENSG00000124391	16	88638571	88640468	IL17C	protein_coding	Yes	NM_013278.4	5.9876	-4.8029	9.9997	1.6E-03	3.6E-02
**ENST00000507448**	ENSG00000251535	4	336257	336786	-	processed_pseudogene	Yes	-	2.6415	-3.4600	9.9950	1.6E-03	3.6E-02
**ENST00000445539**	ENSG00000237827	10	103125016	103125416	RPS15AP29	processed_pseudogene	Yes	-	-1.0971	-1.0600	9.9941	1.6E-03	3.6E-02
**ENST00000684052**	ENSG00000125522	20	64103801	64107565	NPBWR2	protein_coding	Yes	NM_005286.4	6.0826	-4.7409	9.9809	1.6E-03	3.7E-02
**ENST00000687171**	ENSG00000289447	6	26285351	26286234	-	lncRNA	Yes	-	1.1308	-1.9033	9.9701	1.6E-03	3.7E-02
**ENST00000665702**	ENSG00000286408	5	133051064	133051893	-	lncRNA	Yes	-	1.1112	-1.7149	9.9577	1.6E-03	3.7E-02
**ENST00000580031**	ENSG00000265713	17	29775746	29776701	-	transcribed_processed_pseudogene	Yes	-	-1.7550	-2.4230	9.9507	1.6E-03	3.7E-02
**ENST00000701353**	ENSG00000289887	13	95273962	95278207	-	lncRNA	Yes	-	1.4430	-2.1563	9.9448	1.6E-03	3.7E-02
**ENST00000361971**	ENSG00000198753	X	153764248	153779341	PLXNB3	protein_coding	Yes	NM_005393.3	3.4538	-2.5591	9.9310	1.6E-03	3.7E-02
**ENST00000432120**	ENSG00000244332	10	94577438	94611238	-	lncRNA	Yes	-	-1.3574	-2.8765	9.9311	1.6E-03	3.7E-02
**ENST00000702436**	ENSG00000260592	16	19459974	19487964	-	lncRNA	Yes	-	1.2375	3.6913	9.9249	1.6E-03	3.7E-02
**ENST00000371126**	ENSG00000198889	X	126549382	126552814	DCAF12L1	protein_coding	Yes	NM_178470.5	6.6633	-4.3472	9.9217	1.6E-03	3.7E-02
**ENST00000564016**	ENSG00000260378	16	12745872	12757835	-	lncRNA	Yes	-	-1.6286	-2.9658	9.9152	1.6E-03	3.7E-02
**ENST00000367155**	ENSG00000162873	1	205336060	205356922	KLHDC8A	protein_coding	Yes	NM_018203.3	1.6597	-3.1012	9.9112	1.6E-03	3.7E-02
**ENST00000605991**	ENSG00000272076	8	51810109	51810681	-	lncRNA	Yes	-	-1.0944	-2.4580	9.9090	1.6E-03	3.7E-02
**ENST00000251453**	ENSG00000105193	19	39433136	39435949	RPS16	protein_coding	Yes	NM_001020.6	1.1819	4.8414	9.9079	1.6E-03	3.7E-02
**ENST00000442828**	ENSG00000227782	17	16040471	16041273	-	lncRNA	Yes	-	-1.5585	-3.4646	9.9039	1.6E-03	3.8E-02
**ENST00000289957**	ENSG00000147432	8	42697365	42737407	CHRNB3	protein_coding	Yes	NM_000749.5	5.6655	-5.0154	9.8862	1.7E-03	3.8E-02
**ENST00000579909**	ENSG00000265195	15	68801849	68801925	MIR4312	miRNA	Yes	-	-1.5343	-2.7411	9.8785	1.7E-03	3.8E-02
**ENST00000334928**	ENSG00000179168	19	38384266	38388034	GGN	protein_coding	Yes	NM_152657.4	4.1129	-3.3576	9.8716	1.7E-03	3.8E-02
**ENST00000582765**	ENSG00000266497	17	46503945	46509339	RDM1P2	unprocessed_pseudogene	Yes	-	-1.5780	-0.8537	9.8561	1.7E-03	3.8E-02
**ENST00000415903**	ENSG00000230240	10	31983884	31984554	RPL34P19	processed_pseudogene	Yes	-	-3.5705	-5.4854	9.8554	1.7E-03	3.8E-02

**ENST00000384519**	ENSG00000207248	16	21642053	21642160	RNU6-1005P	snRNA	Yes	-	-1.7289	-3.9048	9.8490	1.7E-03	3.8E-02
**ENST00000448097**	ENSG00000132510	17	7834216	7854796	KDM6B	protein_coding	Yes	NM_001348716.2	-1.0974	2.9897	9.8483	1.7E-03	3.8E-02
**ENST00000286953**	ENSG00000156411	14	103912287	103921529	ATP5MJ	protein_coding	Yes	NM_004894.3	1.0078	2.6560	9.8299	1.7E-03	3.8E-02
**ENST00000515673**	ENSG00000170153	4	140859806	141133469	RNF150	protein_coding	Yes	NM_020724.2	3.7819	-2.6048	9.8297	1.7E-03	3.8E-02
**ENST00000544463**	ENSG00000255776	12	9036343	9037244	VDAC2P2	processed_pseudogene	Yes	-	-1.7326	-1.6815	9.8259	1.7E-03	3.8E-02
**ENST00000371390**	ENSG00000203987	9	137867924	137892570	-	lncRNA	Yes	-	5.9770	-4.8240	9.8173	1.7E-03	3.9E-02
**ENST00000369500**	ENSG00000173212	1	116111398	116138149	MAB21L3	protein_coding	Yes	NM_152367.3	-1.2665	-1.4475	9.8118	1.7E-03	3.9E-02
**ENST00000652589**	ENSG00000286127	22	21737447	21744108	-	lncRNA	Yes	-	-1.1950	-3.0444	9.8089	1.7E-03	3.9E-02
**ENST00000454123**	ENSG00000229955	22	38387917	38388857	-	lncRNA	Yes	-	-1.0229	-1.6804	9.8027	1.7E-03	3.9E-02
**ENST00000455437**	ENSG00000214651	9	121238116	121240089	-	processed_pseudogene	Yes	-	4.7475	-5.3734	9.7852	1.8E-03	3.9E-02
**ENST00000624035**	ENSG00000279880	17	67973933	67976072	-	TEC	Yes	-	-1.0661	-0.5306	9.7813	1.8E-03	3.9E-02
**ENST00000664890**	ENSG00000287920	6	11291298	11296224	-	lncRNA	Yes	-	-1.2434	-2.0721	9.7794	1.8E-03	3.9E-02
**ENST00000424112**	ENSG00000244502	3	13476981	13480053	HDAC11-AS1	lncRNA	Yes	-	2.8119	-4.8986	9.7714	1.8E-03	3.9E-02
**ENST00000335295**	ENSG00000244734	11	5225463	5227071	HBB	protein_coding	Yes	NM_000518.5	2.8037	2.6725	9.7588	1.8E-03	3.9E-02
**ENST00000690015**	ENSG00000226321	2	240906335	240993311	CROCC2	protein_coding	Yes	NM_001351305.2	3.5150	-2.8330	9.7530	1.8E-03	3.9E-02
**ENST00000362011**	ENSG00000198892	1	201888897	201892587	SHISA4	protein_coding	Yes	NM_198149.3	1.3129	-0.6386	9.7492	1.8E-03	3.9E-02
**ENST00000406574**	ENSG00000217512	6	79067691	79068138	-	processed_pseudogene	Yes	-	-1.0184	-1.8072	9.7406	1.8E-03	3.9E-02
**ENST00000609910**	ENSG00000273017	21	29359001	29359453	-	lncRNA	Yes	-	-1.4835	-2.6034	9.7148	1.8E-03	4.0E-02
**ENST00000563286**	ENSG00000259959	4	47840121	47844339	-	lncRNA	Yes	-	-1.1638	0.6829	9.6959	1.8E-03	4.0E-02
**ENST00000688494**	ENSG00000289236	20	62120045	62121762	-	lncRNA	Yes	-	4.5210	-4.7888	9.6937	1.8E-03	4.0E-02
**ENST00000590968**	ENSG00000267175	18	61592374	61748832	-	lncRNA	Yes	-	1.2178	-1.0870	9.6866	1.9E-03	4.0E-02
**ENST00000272134**	ENSG00000243709	1	225886281	225889146	LEFTY1	protein_coding	Yes	NM_020997.4	1.3906	-2.0695	9.6839	1.9E-03	4.0E-02
**ENST00000550637**	ENSG00000257639	16	21532944	21550373	-	unprocessed_pseudogene	Yes	-	4.7022	-5.3622	9.6789	1.9E-03	4.0E-02
**ENST00000270631**	ENSG00000142538	19	49422418	49423441	PTH2	protein_coding	Yes	NM_178449.4	6.1436	-4.7438	9.6506	1.9E-03	4.1E-02

**ENST00000540052**	ENSG00000085185	X	129982634	130058071	BCORL1	protein_coding	Yes	NM_001379451.1	-1.0977	-0.4113	9.6446	1.9E-03	4.1E-02
**ENST00000339579**	ENSG00000187762	6	35023521	35023831	HSPE1P11	processed_pseudogene	Yes	-	-2.0812	-4.1151	9.6386	1.9E-03	4.1E-02
**ENST00000602889**	ENSG00000269967	1	31851912	31921841	-	lncRNA	Yes	-	-1.3387	-1.6699	9.6088	1.9E-03	4.1E-02
**ENST00000281589**	ENSG00000151846	13	25096135	25099254	PABPC3	protein_coding	Yes	NM_030979.3	-1.1963	-1.1280	9.5970	1.9E-03	4.1E-02
**ENST00000607315**	ENSG00000271869	8	30155827	30156633	DCTN6-DT	lncRNA	Yes	-	1.1861	-1.8567	9.5966	1.9E-03	4.1E-02
**ENST00000690275**	ENSG00000289380	1	13757777	13758465	-	lncRNA	Yes	-	-1.0966	-1.7434	9.5895	2.0E-03	4.1E-02
**ENST00000222145**	ENSG00000105538	19	48720584	48740610	RASIP1	protein_coding	Yes	NM_017805.3	4.0780	-2.8588	9.5800	2.0E-03	4.1E-02
**ENST00000619945**	ENSG00000276398	12	97560855	97561048	-	misc_RNA	Yes	-	2.9567	-5.0769	9.5648	2.0E-03	4.2E-02
**ENST00000362760**	ENSG00000199630	6	89841328	89841424	Y_RNA	misc_RNA	Yes	-	-3.5342	-4.8086	9.5577	2.0E-03	4.2E-02
**ENST00000289968**	ENSG00000140750	16	24919388	25015369	ARHGAP17	protein_coding	Yes	NM_001006634.3	-1.0044	1.3286	9.5284	2.0E-03	4.2E-02
**ENST00000429893**	ENSG00000237788	20	49829124	49831085	-	processed_pseudogene	Yes	-	-1.5391	-2.1883	9.5234	2.0E-03	4.2E-02
**ENST00000685046**	ENSG00000288803	1	110680507	110682674	-	lncRNA	Yes	-	-1.2711	-2.6685	9.5197	2.0E-03	4.2E-02
**ENST00000406610**	ENSG00000116117	2	204545474	205620162	PARD3B	protein_coding	Yes	NM_001302769.2	1.7473	-2.0440	9.4859	2.1E-03	4.3E-02
**ENST00000621054**	ENSG00000273573	1	181668748	181669028	Metazoa_SRP	misc_RNA	Yes	-	-3.1787	-5.4510	9.4695	2.1E-03	4.3E-02
**ENST00000443558**	ENSG00000229273	9	39809561	39810019	-	processed_pseudogene	Yes	-	1.2546	-3.1163	9.4611	2.1E-03	4.3E-02
**ENST00000259254**	ENSG00000136732	2	126656157	126696667	GYPC	protein_coding	Yes	NM_002101.5	1.0228	7.1409	9.4446	2.1E-03	4.3E-02
**ENST00000602312**	ENSG00000183921	16	22162505	22191751	SDR42E2	protein_coding	Yes	NM_001394319.2	1.8730	-3.5317	9.4403	2.1E-03	4.3E-02
**ENST00000683648**	ENSG00000114654	3	129001303	129040742	EFCC1	protein_coding	Yes	NM_001377500.1	2.8480	-3.1126	9.4393	2.1E-03	4.3E-02
**ENST00000494408**	ENSG00000224858	3	37016522	37017014	RPL29P11	processed_pseudogene	Yes	-	1.0203	2.5900	9.4372	2.1E-03	4.3E-02
**ENST00000384919**	ENSG00000207652	13	40810765	40810861	MIR621	miRNA	Yes	-	1.2891	-3.4899	9.4290	2.1E-03	4.3E-02
**ENST00000620215**	ENSG00000274611	17	38181658	38192555	TBC1D3	protein_coding	Yes	NM_001123391.4	-1.2235	-2.1299	9.4163	2.2E-03	4.3E-02
**ENST00000371069**	ENSG00000116675	1	65309542	65415871	DNAJC6	protein_coding	Yes	NM_001256864.2	1.0494	5.2329	9.4061	2.2E-03	4.4E-02
**ENST00000409874**	ENSG00000156414	14	103928455	104052667	TDRD9	protein_coding	Yes	NM_153046.3	-1.3857	0.8315	9.4000	2.2E-03	4.4E-02
**ENST00000585656**	ENSG00000267477	19	11466239	11505698	-	protein_coding	Yes	-	1.0261	-2.1268	9.3949	2.2E-03	4.4E-02

**ENST00000642861**	ENSG00000285486	17	44276367	44281182	-	lncRNA	Yes	-	1.5600	-2.9504	9.3927	2.2E-03	4.4E-02
**ENST00000334575**	ENSG00000243955	6	52791370	52803816	GSTA1	protein_coding	Yes	NM_145740.5	2.1056	-3.3068	9.3888	2.2E-03	4.4E-02
**ENST00000565058**	ENSG00000260711	14	91752855	91759798	-	lncRNA	Yes	-	-1.0115	0.9632	9.3876	2.2E-03	4.4E-02
**ENST00000331495**	ENSG00000184922	17	45221884	45247318	FMNL1	protein_coding	Yes	NM_005892.4	-1.0336	3.4244	9.3812	2.2E-03	4.4E-02
**ENST00000686301**	ENSG00000214401	17	46193567	46197842	KANSL1-AS1	lncRNA	Yes	-	1.0262	-0.2034	9.3216	2.3E-03	4.5E-02
**ENST00000688446**	ENSG00000289630	7	113118665	113119137	-	lncRNA	Yes	-	4.3078	-5.4994	9.3168	2.3E-03	4.5E-02
**ENST00000542817**	ENSG00000169246	16	21402236	21405319	NPIPB3	protein_coding	Yes	-	-1.1800	-1.6850	9.3164	2.3E-03	4.5E-02
**ENST00000685826**	ENSG00000289480	4	82348251	82349441	-	lncRNA	Yes	-	-1.0907	-2.2244	9.3127	2.3E-03	4.5E-02
**ENST00000510153**	ENSG00000251093	5	91226474	91227071	-	lncRNA	Yes	-	-1.1346	-2.4219	9.2927	2.3E-03	4.5E-02
**ENST00000624864**	ENSG00000280035	8	141224287	141226947	-	TEC	Yes	-	-1.0182	-0.2309	9.2706	2.3E-03	4.5E-02
**ENST00000335678**	ENSG00000185974	13	113667218	113737736	GRK1	protein_coding	Yes	NM_002929.3	6.1991	-4.7242	9.2612	2.3E-03	4.6E-02
**ENST00000563477**	ENSG00000260517	16	29139660	29216706	-	lncRNA	Yes	-	-1.0198	-2.3575	9.2588	2.3E-03	4.6E-02
**ENST00000460608**	ENSG00000240729	5	177264765	177265241	-	processed_pseudogene	Yes	-	-1.1696	-3.3202	9.2411	2.4E-03	4.6E-02
**ENST00000516201**	ENSG00000252010	2	233275726	233276002	SCARNA5	scaRNA	Yes	-	1.1489	1.9018	9.2219	2.4E-03	4.6E-02
**ENST00000585129**	ENSG00000265123	6	34685354	34685642	RN7SL200P	misc_RNA	Yes	-	-2.4772	-4.6269	9.1944	2.4E-03	4.6E-02
**ENST00000611748**	ENSG00000290425	22	18538055	18577968	PI4KAP1	lncRNA	Yes	-	1.8105	-0.7037	9.1919	2.4E-03	4.6E-02
**ENST00000624849**	ENSG00000279320	3	146504569	146506560	-	TEC	Yes	-	-1.0496	-1.0284	9.1912	2.4E-03	4.6E-02
**ENST00000408887**	ENSG00000221949	12	62601750	62603690	LINC01465	lncRNA	Yes	-	1.1433	-2.2485	9.1564	2.5E-03	4.7E-02
**ENST00000612579**	ENSG00000274602	22	18534004	18548798	PI4KAP1	transcribed_unprocessed_pseudogene	Yes	-	1.2253	-0.2198	9.1479	2.5E-03	4.7E-02
**ENST00000624597**	ENSG00000279940	12	130956383	130958031	-	TEC	Yes	-	5.4973	-5.1251	9.1435	2.5E-03	4.7E-02
**ENST00000373414**	ENSG00000288705	2	233712906	233773300	UGT1A5	protein_coding	Yes	NM_019078.2	5.3262	-5.1981	9.1360	2.5E-03	4.7E-02
**ENST00000558717**	ENSG00000259671	15	58154959	58156061	MTCYBP23	processed_pseudogene	Yes	-	-1.7683	-3.0283	9.1292	2.5E-03	4.7E-02
**ENST00000624324**	ENSG00000279198	19	16074291	16077395	-	TEC	Yes	-	1.0235	1.0432	9.1273	2.5E-03	4.7E-02
**ENST00000377574**	ENSG00000197891	11	64591219	64602344	SLC22A12	protein_coding	Yes	NM_144585.4	5.8110	-4.9304	9.1242	2.5E-03	4.7E-02
**ENST00000580121**	ENSG00000265784	17	38918800	38921769	-	lncRNA	Yes	-	-1.0238	-2.5225	9.1200	2.5E-03	4.7E-02

**ENST00000501387**	ENSG00000247131	12	69713632	69738574	-	lncRNA	Yes	-	5.9151	-4.8865	9.1181	2.5E-03	4.7E-02
**ENST00000569407**	ENSG00000261158	16	12759281	12761162	-	lncRNA	Yes	-	-1.7628	-3.6513	9.1182	2.5E-03	4.7E-02
**ENST00000359106**	ENSG00000006283	17	50560714	50627474	CACNA1G	protein_coding	Yes	NM_018896.5	4.9229	-3.0842	9.1177	2.5E-03	4.7E-02
**ENST00000516107**	ENSG00000251916	6	32549939	32550090	RNU1-61P	snRNA	Yes	-	-3.7691	-5.1695	9.1144	2.5E-03	4.7E-02
**ENST00000603829**	ENSG00000270804	19	57867969	57868834	-	transcribed_processed_pseudogene	Yes	-	-1.1152	-2.3785	9.1052	2.5E-03	4.7E-02
**ENST00000524768**	ENSG00000254826	11	75775903	75776929	-	lncRNA	Yes	-	-1.3081	-2.2220	9.1047	2.5E-03	4.7E-02
**ENST00000669254**	ENSG00000272695	13	113864111	113866834	GAS6-DT	lncRNA	Yes	-	6.2085	-4.6735	9.0947	2.6E-03	4.8E-02
**ENST00000379268**	ENSG00000186891	1	1203507	1206592	TNFRSF18	protein_coding	Yes	NM_004195.3	1.0998	-1.2068	9.0822	2.6E-03	4.8E-02
**ENST00000540069**	ENSG00000105509	19	51713111	51723991	HAS1	protein_coding	Yes	NM_001297436.2	3.6702	-3.4466	9.0617	2.6E-03	4.8E-02
**ENST00000524047**	ENSG00000253641	8	10474564	10481974	LINC03022	lncRNA	Yes	-	-1.2879	-2.9893	9.0534	2.6E-03	4.8E-02
**ENST00000515777**	ENSG00000187821	4	185018489	185020953	HELT	protein_coding	Yes	NM_001300781.2	5.5697	-5.0531	9.0514	2.6E-03	4.8E-02
**ENST00000361824**	ENSG00000143452	1	150698059	150720895	HORMAD1	protein_coding	Yes	NM_032132.5	-1.0312	-0.5129	9.0401	2.6E-03	4.8E-02
**ENST00000549373**	ENSG00000258337	12	116174501	116181295	-	lncRNA	Yes	-	-1.1699	-0.8193	9.0363	2.6E-03	4.8E-02
**ENST00000424928**	ENSG00000244510	7	66480393	66490108	-	transcribed_unprocessed_pseudogene	Yes	-	1.0737	-1.4696	9.0340	2.7E-03	4.8E-02
**ENST00000316967**	ENSG00000290689	15	84199311	84230136	-	lncRNA	Yes	-	1.0166	-2.1636	9.0328	2.7E-03	4.8E-02
**ENST00000514074**	ENSG00000250053	4	190037915	190038399	RARRES2P4	processed_pseudogene	Yes	-	3.8482	-4.0135	9.0246	2.7E-03	4.9E-02
**ENST00000622948**	ENSG00000279276	16	23962950	23963351	-	TEC	Yes	-	-1.2224	-2.1210	9.0206	2.7E-03	4.9E-02
**ENST00000689830**	ENSG00000289416	7	142795707	142795980	-	lncRNA	Yes	-	1.3484	-3.4017	9.0075	2.7E-03	4.9E-02
**ENST00000422216**	ENSG00000226252	1	47225796	47230750	-	lncRNA	Yes	-	1.1555	-0.3830	8.9964	2.7E-03	4.9E-02
**ENST00000307886**	ENSG00000172543	11	65879836	65883741	CTSW	protein_coding	Yes	NM_001335.4	-1.0812	1.2838	8.9884	2.7E-03	4.9E-02
**ENST00000430912**	ENSG00000229848	17	81514046	81527243	-	lncRNA	Yes	-	2.3837	-4.6085	8.9855	2.7E-03	4.9E-02
**ENST00000511627**	ENSG00000253102	17	50396437	50397888	-	lncRNA	Yes	-	1.3155	-3.1125	8.9528	2.8E-03	5.0E-02
**ENST00000622206**	ENSG00000278299	17	38057692	38068634	TBC1D3C	protein_coding	Yes	NM_001001418.6	-1.1806	-2.8960	8.9477	2.8E-03	5.0E-02

**ENST00000691638**	ENSG00000188766	19	38388696	38399587	SPRED3	protein_coding	Yes	NM_001394336.1	3.2624	-3.3994	8.9437	2.8E-03	5.0E-02
**ENST00000484033**	ENSG00000240853	9	131725167	131725467	RN7SL328P	misc_RNA	Yes	-	-1.2151	-2.4285	8.9430	2.8E-03	5.0E-02
**ENST00000480487**	ENSG00000240463	14	34568688	34569129	RPS19P3	processed_pseudogene	Yes	-	-1.2037	-2.9186	8.9350	2.8E-03	5.0E-02
**ENST00000689488**	ENSG00000289468	11	3706680	3707754	-	lncRNA	Yes	-	-1.1814	-2.2705	8.9302	2.8E-03	5.0E-02

Supplementary table 4: Differentially expressed genes (Female MwoA patients vs. Female controls). Log_2FC – Log 2-fold change; logCPM – log counts per million; LR – likelihood ratio; FDR – False discovery rate

**Transcript**	**Transcript stable ID**	**chr**	**start**	**end**	**Gene name**	**Gene type**	**Canonical**	**MANE Select**	**log_2FC**	**logCPM**	**LR**	**P-Value**	**FDR**
**ENST00000315576**	ENSG00000127507	19	14732391	14778560	ADGRE2	protein_coding	Yes	NM_013447.4	-1.2319	3.7268	36.4175	1.6E-09	0.0003
**ENST00000433113**	ENSG00000240527	10	95833507	95873758	-	lncRNA	Yes	-	-2.0246	-0.9573	28.6340	8.7E-08	0.0044
**ENST00000293973**	ENSG00000162068	16	2471296	2474145	NTN3	protein_coding	Yes	NM_006181.3	4.6814	-2.3723	23.6522	1.2E-06	0.0072
**ENST00000437890**	ENSG00000228929	1	52772193	52772648	RPS13P2	processed_pseudogene	Yes	-	1.0850	3.5771	25.5926	4.2E-07	0.0072
**ENST00000495392**	ENSG00000227081	12	3211662	3211917	-	processed_pseudogene	Yes	-	2.0657	6.7716	22.2705	2.4E-06	0.0105
**ENST00000689584**	ENSG00000181218	1	228457363	228457873	H2AW	protein_coding	Yes	NM_033445.3	1.2629	-0.3002	22.2949	2.3E-06	0.0105
**ENST00000358894**	ENSG00000101203	20	63293185	63334806	COL20A1	protein_coding	Yes	NM_020882.4	6.6614	-2.8444	21.1739	4.2E-06	0.0136
**ENST00000317633**	ENSG00000172058	5	70900668	70908115	SERF1A	protein_coding	Yes	NM_022968.2	1.3324	-1.3141	20.9854	4.6E-06	0.0140
**ENST00000495531**	ENSG00000220842	10	120354700	120355183	RPL21P16	processed_pseudogene	Yes	-	1.0855	7.8337	20.8917	4.9E-06	0.0140
**ENST00000610913**	ENSG00000278195	22	37204236	37212477	SSTR3	protein_coding	Yes	NM_001051.5	1.1679	0.0335	20.8999	4.8E-06	0.0140
**ENST00000342058**	ENSG00000140092	14	91869410	91947694	FBLN5	protein_coding	Yes	NM_006329.4	1.4165	-0.6212	20.4889	6.0E-06	0.0143
**ENST00000378585**	ENSG00000187730	1	2019344	2030758	GABRD	protein_coding	Yes	NM_000815.5	4.9189	-2.8786	20.5036	6.0E-06	0.0143
**ENST00000407683**	ENSG00000204099	2	241809192	241817413	NEU4	protein_coding	Yes	NM_001167600.3	8.5235	-2.9862	20.5236	5.9E-06	0.0143
**ENST00000454486**	ENSG00000223509	15	32523025	32536926	WHAMMP1	transcribed_processed_pseudogene	Yes	-	1.1080	4.3790	20.6841	5.4E-06	0.0143

**ENST00000530158**	ENSG00000254916	11	90051836	90053736	-	unprocessed_pseudogene	Yes	-	7.7051	-3.7099	20.6284	5.6E-06	0.0143
**ENST00000547512**	ENSG00000257246	12	51124627	51125381	PHB1P19	transcribed_processed_pseudogene	Yes	-	-2.8819	-2.1255	20.4236	6.2E-06	0.0144
**ENST00000642557**	ENSG00000235169	1	3772748	3775956	SMIM1	protein_coding	Yes	NM_001288583.2	2.3331	-0.8440	20.1678	7.1E-06	0.0154
**ENST00000296591**	ENSG00000164176	5	83940553	84384880	EDIL3	protein_coding	Yes	NM_005711.5	3.8116	-2.8736	19.9794	7.8E-06	0.0161
**ENST00000302057**	ENSG00000170561	5	2746164	2751677	IRX2	protein_coding	Yes	NM_033267.5	7.9599	-3.5165	19.8529	8.4E-06	0.0162
**ENST00000525634**	ENSG00000110700	11	17074387	17077667	RPS13	protein_coding	Yes	NM_001017.3	1.0956	5.4275	19.5736	9.7E-06	0.0162
**ENST00000590469**	ENSG00000115266	19	1450120	1473244	APC2	protein_coding	Yes	NM_005883.3	4.0059	-0.3915	19.8001	8.6E-06	0.0162
**ENST00000613359**	ENSG00000275215	21	8395606	8395759	RNA5-8SN3	rRNA	Yes	-	4.3479	-1.3453	19.5604	9.7E-06	0.0162
**ENST00000343986**	ENSG00000171858	20	62387102	62388520	RPS21	protein_coding	Yes	NM_001024.4	1.4027	4.8100	19.4926	1.0E-05	0.0162
**ENST00000334018**	ENSG00000186998	22	29205895	29259597	EMID1	protein_coding	Yes	NM_133455.4	1.6086	-1.8705	19.4087	1.1E-05	0.0166
**ENST00000433186**	ENSG00000230789	5	143192499	143194166	ARHGAP26-IT1	lncRNA	Yes	-	-1.4442	-0.4182	19.3022	1.1E-05	0.0166
**ENST00000413027**	ENSG00000227939	6	31280316	31281519	RPL3P2	processed_pseudogene	Yes	-	1.0044	1.1225	18.6659	1.6E-05	0.0188
**ENST00000390295**	ENSG00000211649	22	22369613	22370087	IGLV7-46	IG_V_gene	Yes	-	3.1434	-2.2772	18.5402	1.7E-05	0.0194
**ENST00000645284**	ENSG00000187017	1	6424775	6460944	ESPN	protein_coding	Yes	NM_031475.3	1.5428	0.8454	18.5418	1.7E-05	0.0194
**ENST00000518552**	ENSG00000254325	8	55893594	55895739	-	lncRNA	Yes	-	-2.3094	0.0967	18.5227	1.7E-05	0.0194
**ENST00000311111**	ENSG00000172809	17	74203677	74210655	RPL38	protein_coding	Yes	NM_000999.4	1.3766	5.9658	18.3939	1.8E-05	0.0202
**ENST00000644246**	ENSG00000272449	1	2530063	2547460	-	lncRNA	Yes	-	1.1844	-0.5099	18.2992	1.9E-05	0.0207
**ENST00000622824**	ENSG00000198488	11	77034397	77041973	B3GNT6	protein_coding	Yes	NM_138706.5	7.5073	-3.9068	18.0451	2.2E-05	0.0220
**ENST00000682832**	ENSG00000142609	1	1921956	2003786	CFAP74	protein_coding	Yes	NM_001304360.2	6.0689	-2.9477	18.0641	2.1E-05	0.0220
**ENST00000406610**	ENSG00000116117	2	204545474	205620162	PARD3B	protein_coding	Yes	NM_001302769.2	2.3544	-1.8502	17.9708	2.2E-05	0.0224
**ENST00000400299**	ENSG00000198832	22	31104776	31107568	SELENOM	protein_coding	Yes	NM_080430.4	1.0477	-0.5535	17.8312	2.4E-05	0.0234
**ENST00000392607**	ENSG00000197177	10	133087923	133131675	ADGRA1	protein_coding	Yes	NM_001083909.3	6.7476	-2.7643	17.6307	2.7E-05	0.0246
**ENST00000382723**	ENSG00000163132	4	4859664	4863936	MSX1	protein_coding	Yes	NM_002448.3	7.4538	-3.9471	17.5887	2.7E-05	0.0249

**ENST00000449051**	ENSG00000224807	4	190021406	190022665	DUX4L9	unprocessed_ pseudogene	Yes	-	6.9202	-3.2817	17.5503	2.8E-05	0.0250
**ENST00000335750**	ENSG00000021645	14	78170372	79868291	NRXN3	protein_coding	Yes	NM_00133019 5.2	1.5780	-0.3031	17.5132	2.9E-05	0.0252
**ENST00000310581**	ENSG00000164362	5	1253166	1295068	TERT	protein_coding	Yes	NM_198253.3	4.9800	-2.1425	17.5005	2.9E-05	0.0253
**ENST00000569722**	ENSG00000267127	18	80034345	80097088	-	protein_coding	Yes	-	1.8013	-2.1064	17.4023	3.0E-05	0.0262
**ENST00000393087**	ENSG00000197249	14	94376746	94388602	SERPINA 1	protein_coding	Yes	NM_000295.5	-1.6277	4.1905	17.3598	3.1E-05	0.0263
**ENST00000427575**	ENSG00000129932	19	3490823	3500674	DOHH	protein_coding	Yes	NM_00114516 5.2	1.0346	-0.1731	17.2943	3.2E-05	0.0264
**ENST00000651111**	ENSG00000241489	X	149477103	149540926	-	protein_coding	Yes	-	-1.3648	4.4415	17.3041	3.2E-05	0.0264
**ENST00000222145**	ENSG00000105538	19	48720584	48740610	RASIP1	protein_coding	Yes	NM_017805.3	4.9694	-2.4039	17.1072	3.5E-05	0.0272
**ENST00000445877**	ENSG00000178966	9	83980804	84004074	RMI1	protein_coding	Yes	NM_00135829 1.2	-1.0067	1.2479	17.1074	3.5E-05	0.0272
**ENST00000264554**	ENSG00000129946	19	416588	461033	SHC2	protein_coding	Yes	NM_012435.3	4.3216	-2.7232	16.8389	4.1E-05	0.0285
**ENST00000611191**	ENSG00000273797	14	69617121	69617648	-	lncRNA	Yes	-	-1.2389	-0.8549	16.8087	4.1E-05	0.0288
**ENST00000593581**	ENSG00000269386	19	8374371	8390685	RAB11B-AS1	lncRNA	Yes	-	1.1794	-0.9933	16.7594	4.2E-05	0.0291
**ENST00000683648**	ENSG00000114654	3	129001303	129040742	EFCC1	protein_coding	Yes	NM_00137750 0.1	3.6818	-2.7318	16.7301	4.3E-05	0.0291
**ENST00000340855**	ENSG00000010404	X	149476987	149505306	IDS	protein_coding	Yes	NM_000202.8	-1.3437	4.5240	16.6343	4.5E-05	0.0298
**ENST00000429036**	ENSG00000125895	20	1180569	1184981	TMEM74B	protein_coding	Yes	NM_00130474 8.2	6.8895	-4.3822	16.5571	4.7E-05	0.0303
**ENST00000597582**	ENSG00000152475	19	58367622	58381030	ZNF837	protein_coding	Yes	NM_138466.2	2.0155	-1.2040	16.5514	4.7E-05	0.0303
**ENST00000663947**	ENSG00000286314	5	65965372	65969143	-	lncRNA	Yes	-	-1.4300	0.5919	16.5206	4.8E-05	0.0304
**ENST00000263610**	ENSG00000125492	9	132582605	132590252	BARHL1	protein_coding	Yes	NM_020064.4	7.7229	-3.7517	16.4151	5.1E-05	0.0311
**ENST00000318562**	ENSG00000177106	11	706230	727727	EPS8L2	protein_coding	Yes	NM_022772.4	1.4972	-0.8763	16.4215	5.1E-05	0.0311
**ENST00000569446**	ENSG00000261678	8	144330564	144336482	SCRT1	protein_coding	Yes	NM_031309.6	5.8763	-2.8488	16.4161	5.1E-05	0.0311
**ENST00000274181**	ENSG00000145536	5	5140329	5320304	ADAMTS1 6	protein_coding	Yes	NM_139056.4	7.3149	-4.0810	16.3670	5.2E-05	0.0313
**ENST00000258381**	ENSG00000135899	2	230165185	230219984	SP110	protein_coding	Yes	NM_080424.4	-1.2538	3.7166	16.1145	6.0E-05	0.0320
**ENST00000429111**	ENSG00000224083	9	5096665	5098193	MTCO1P1 1	unprocessed_ pseudogene	Yes	-	-2.4856	-2.1811	16.2044	5.7E-05	0.0320

**ENST00000545450**	ENSG00000102854	16	760733	768862	MSLN	protein_coding	Yes	NM_005823.6	7.2810	-2.9513	16.2678	5.5E-05	0.0320
**ENST00000594472**	ENSG00000229692	2	38992278	38993836	SOS1-IT1	lncRNA	Yes	-	-1.1294	-0.5170	16.1284	5.9E-05	0.0320
**ENST00000611590**	ENSG00000182612	17	81637195	81648749	TSPAN10	protein_coding	Yes	NM_001290212.2	4.3145	-3.1496	16.1539	5.8E-05	0.0320
**ENST00000665353**	ENSG00000286940	6	31479972	31494935	MICB-DT	lncRNA	Yes	-	-1.1818	-1.2116	16.3106	5.4E-05	0.0320
**ENST00000691638**	ENSG00000188766	19	38388696	38399587	SPRED3	protein_coding	Yes	NM_001394336.1	4.1011	-3.0216	16.0336	6.2E-05	0.0320
**ENST00000691717**	ENSG00000289353	16	66567155	66568898	-	lncRNA	Yes	-	-1.3102	1.8011	16.1847	5.7E-05	0.0320
**ENST00000691901**	ENSG00000145198	3	184229584	184242329	VWA5B2	protein_coding	Yes	NM_001390846.1	5.5685	-2.7933	16.1558	5.8E-05	0.0320
**ENST00000264033**	ENSG00000110395	11	119206338	119308149	CBL	protein_coding	Yes	NM_005188.4	-1.4977	4.5633	15.9852	6.4E-05	0.0321
**ENST00000507317**	ENSG00000251215	5	39169209	39170335	GOLGA5P1	processed_pseudogene	Yes	-	-1.5974	0.6128	15.8922	6.7E-05	0.0330
**ENST00000300146**	ENSG00000166889	11	59636715	59669037	PATL1	protein_coding	Yes	NM_152716.3	-1.1017	1.7298	15.8701	6.8E-05	0.0331
**ENST00000605424**	ENSG00000271447	17	35765864	35795641	MMP28	protein_coding	Yes	NM_024302.5	1.7907	-2.1403	15.8616	6.8E-05	0.0331
**ENST00000669254**	ENSG00000272695	13	113864111	113866834	GAS6-DT	lncRNA	Yes	-	7.0780	-4.2537	15.8653	6.8E-05	0.0331
**ENST00000343516**	ENSG00000167971	16	2177179	2196605	CASKIN1	protein_coding	Yes	NM_020764.4	3.6691	-1.1552	15.8562	6.8E-05	0.0332
**ENST00000440769**	ENSG00000234292	5	91280096	91281142	-	lncRNA	Yes	-	-1.7803	-0.1601	15.8296	6.9E-05	0.0332
**ENST00000233948**	ENSG00000115596	2	218859804	218874233	WNT6	protein_coding	Yes	NM_006522.4	5.4767	-3.2468	15.7792	7.1E-05	0.0337
**ENST00000379289**	ENSG00000162571	1	1173879	1197936	TTLL10	protein_coding	Yes	NM_001130045.2	4.8318	-2.7298	15.7777	7.1E-05	0.0337
**ENST00000473748**	ENSG00000227097	11	82689558	82689768	RPS28P7	processed_pseudogene	Yes	-	1.4355	4.5260	15.7218	7.3E-05	0.0343
**ENST00000598249**	ENSG00000160460	19	40467000	40576464	SPTBN4	protein_coding	Yes	NM_020971.3	3.7987	-1.3884	15.7275	7.3E-05	0.0343
**ENST00000293441**	ENSG00000161681	19	50659254	50719802	SHANK1	protein_coding	Yes	NM_016148.5	4.0329	-1.3106	15.6792	7.5E-05	0.0344
**ENST00000240328**	ENSG00000121068	17	61399842	61409466	TBX2	protein_coding	Yes	NM_005994.4	6.0362	-3.3855	15.6394	7.7E-05	0.0344
**ENST00000319653**	ENSG00000155816	1	240091882	240475187	FMN2	protein_coding	Yes	NM_020066.5	6.6915	-3.5009	15.6382	7.7E-05	0.0344
**ENST00000659311**	ENSG00000215244	10	6271168	6353827	LINC02649	lncRNA	Yes	-	-1.4587	-0.3436	15.6495	7.6E-05	0.0344
**ENST00000689970**	ENSG00000223855	7	522572	530163	PDGFA-DT	lncRNA	Yes	-	1.5750	-0.5547	15.6392	7.7E-05	0.0344
**ENST00000684052**	ENSG00000125522	20	64103801	64107565	NPBWR2	protein_coding	Yes	NM_005286.4	6.9674	-4.3284	15.5917	7.9E-05	0.0347
**ENST00000622683**	ENSG00000275832	17	38428463	38512385	ARHGAP23	protein_coding	Yes	NM_001199417.2	3.2432	-2.0715	15.5635	8.0E-05	0.0350

**ENST00000303746**	ENSG00000131650	16	2964274	2968380	KREMEN2	protein_coding	Yes	NM_172229.3	4.7118	-2.7789	15.5231	8.2E-05	0.0353
**ENST00000371757**	ENSG00000107147	9	135702184	135795502	KCNT1	protein_coding	Yes	NM_020822.3	4.5792	-2.1438	15.5191	8.2E-05	0.0353
**ENST00000361971**	ENSG00000198753	X	153764248	153779341	PLXNB3	protein_coding	Yes	NM_005393.3	4.3219	-2.1351	15.4521	8.5E-05	0.0359
**ENST00000390981**	ENSG00000212283	2	101272935	101273049	SNORD89	snoRNA	Yes	-	-1.4196	-0.1267	15.3430	9.0E-05	0.0359
**ENST00000432992**	ENSG00000007237	17	9910605	10198606	GAS7	protein_coding	Yes	NM_201433.2	-1.2117	3.4189	15.3868	8.8E-05	0.0359
**ENST00000445535**	ENSG00000225217	1	161606290	161608217	HSPA7	unprocessed_ pseudogene	Yes	-	-1.0856	1.0135	15.3474	8.9E-05	0.0359
**ENST00000450128**	ENSG00000227262	6	29925982	29926973	HCG4B	unprocessed_ pseudogene	Yes	-	2.3339	-2.7246	15.4731	8.4E-05	0.0359
**ENST00000451143**	ENSG00000225900	2	39098148	39098445	HSPE1P1 3	processed_ps eudogene	Yes	-	-2.8295	-2.6495	15.3761	8.8E-05	0.0359
**ENST00000514074**	ENSG00000250053	4	190037915	190038399	RARRES2 P4	processed_ps eudogene	Yes	-	4.7117	-3.6328	15.3437	9.0E-05	0.0359
**ENST00000561440**	ENSG00000284803	15	82533174	82540008	-	lncRNA	Yes	-	1.1109	6.0094	15.3567	8.9E-05	0.0359
**ENST00000657854**	ENSG00000245552	11	95151692	95234391	LNCRNA- IUR	lncRNA	Yes	-	1.4949	1.3504	15.3372	9.0E-05	0.0359
**ENST00000244537**	ENSG00000274618	6	26240392	26240793	H4C6	protein_coding	Yes	NM_003540.4	1.3203	2.8641	15.2566	9.4E-05	0.0365
**ENST00000369096**	ENSG00000057657	6	106086335	106109938	PRDM1	protein_coding	Yes	NM_001198.4	-1.1595	2.3325	15.2659	9.3E-05	0.0365
**ENST00000410180**	ENSG00000222112	1	33336565	33336864	RN7SKP1 6	misc_RNA	Yes	-	-2.0027	-0.8798	15.2693	9.3E-05	0.0365
**ENST00000615155**	ENSG00000236438	3	198180754	198190365	FAM157A	transcribed_un processed_ps eudogene	Yes	-	-1.6526	-0.2378	15.2701	9.3E-05	0.0365
**ENST00000638797**	ENSG00000260220	9	136249972	136304094	CCDC187	protein_coding	Yes	NM_00137818 8.1	4.4660	-2.0751	15.2664	9.3E-05	0.0365
**ENST00000229281**	ENSG00000111678	12	6944021	6946002	C12orf57	protein_coding	Yes	NM_138425.4	1.0187	2.6396	15.2207	9.6E-05	0.0366
**ENST00000284523**	ENSG00000154342	1	228006997	228061271	WNT3A	protein_coding	Yes	NM_033131.4	6.5334	-3.5935	15.2182	9.6E-05	0.0366
**ENST00000289746**	ENSG00000129910	16	89171747	89195492	CDH15	protein_coding	Yes	NM_004933.3	6.0279	-2.8029	15.1171	1.0E-04	0.0367
**ENST00000310613**	ENSG00000173597	4	69721166	69760620	SULT1B1	protein_coding	Yes	NM_014465.4	-1.0363	4.2338	15.1064	1.0E-04	0.0367
**ENST00000328957**	ENSG00000182870	12	132196371	132329589	GALNT9	protein_coding	Yes	NM_00112263 6.2	4.2382	-2.6152	15.1280	1.0E-04	0.0367
**ENST00000373631**	ENSG00000148204	9	123356196	123378753	CRB2	protein_coding	Yes	NM_173689.7	4.6205	-2.2800	15.1321	1.0E-04	0.0367
**ENST00000397770**	ENSG00000076344	16	268306	275944	RGS11	protein_coding	Yes	NM_183337.3	6.6020	-2.8642	15.1008	1.0E-04	0.0367

**ENST00000581811**	ENSG00000274963	1	150568970	150569269	RN7SL600P	misc_RNA	Yes	-	-1.8084	-0.2241	15.1394	1.0E-04	0.0367
**ENST00000624034**	ENSG00000196366	9	136483494	136486067	C9orf163	lncRNA	Yes	-	3.1249	-2.7081	15.1829	9.8E-05	0.0367
**ENST00000204604**	ENSG00000090539	3	184380053	184389829	CHRD	protein_coding	Yes	NM_003741.4	7.8009	-3.6753	15.0667	1.0E-04	0.0368
**ENST00000663752**	ENSG00000286489	10	101694400	101705119	-	lncRNA	Yes	-	2.4027	-3.2576	15.0823	1.0E-04	0.0368
**ENST00000690015**	ENSG00000226321	2	240906335	240993311	CROCC2	protein_coding	Yes	NM_001351305.2	4.3858	-2.4033	15.0505	1.0E-04	0.0368
**ENST00000338448**	ENSG00000099864	19	708934	748329	PALM	protein_coding	Yes	NM_002579.3	5.1224	-2.3601	15.0207	1.1E-04	0.0368
**ENST00000565768**	ENSG00000291105	16	56617422	56618832	-	lncRNA	Yes	-	2.6648	-2.2516	15.0161	1.1E-04	0.0368
**ENST00000581659**	ENSG00000266017	9	63819573	63819654	MIR4477B	miRNA	Yes	-	-1.3470	-1.5699	15.0164	1.1E-04	0.0368
**ENST00000291495**	ENSG00000160161	19	19538264	19546659	CILP2	protein_coding	Yes	NM_153221.2	5.7384	-1.9746	14.9604	1.1E-04	0.0369
**ENST00000359106**	ENSG00000006283	17	50560714	50627474	CACNA1G	protein_coding	Yes	NM_018896.5	5.8488	-2.5822	14.9551	1.1E-04	0.0369
**ENST00000590071**	ENSG00000142279	19	34481757	34512304	WTIP	protein_coding	Yes	NM_001080436.2	3.7665	-2.6590	14.9994	1.1E-04	0.0369
**ENST00000215582**	ENSG00000099812	19	751111	764318	MISP	protein_coding	Yes	NM_173481.4	7.8735	-3.6167	14.9129	1.1E-04	0.0373
**ENST00000425639**	ENSG00000215474	18	47206168	47251660	SKOR2	protein_coding	Yes	NM_001278063.4	5.3275	-3.0077	14.9126	1.1E-04	0.0373
**ENST00000304613**	ENSG00000171798	10	133160218	133226412	KNDC1	protein_coding	Yes	NM_152643.8	3.2472	-1.2033	14.8542	1.2E-04	0.0382
**ENST00000233057**	ENSG00000055332	2	37099209	37156980	EIF2AK2	protein_coding	Yes	NM_001135651.3	-1.0126	4.1401	14.7591	1.2E-04	0.0383
**ENST00000528841**	ENSG00000174672	11	1389933	1462689	BRSK2	protein_coding	Yes	NM_001256627.2	3.6004	-1.9888	14.7320	1.2E-04	0.0384
**ENST00000162330**	ENSG00000050820	16	75228180	75251624	BCAR1	protein_coding	Yes	NM_014567.5	6.3185	-3.1799	14.7238	1.2E-04	0.0384
**ENST00000620884**	ENSG00000284067	7	44051765	44051829	MIR6837	miRNA	Yes	-	1.7662	0.7528	14.7266	1.2E-04	0.0384
**ENST00000423312**	ENSG00000066735	14	104138586	104180894	KIF26A	protein_coding	Yes	NM_015656.2	3.7329	-1.3698	14.6815	1.3E-04	0.0386
**ENST00000274853**	ENSG00000146112	6	30676388	30686645	PPP1R18	protein_coding	Yes	NM_133471.4	-1.1155	2.7127	14.6235	1.3E-04	0.0390
**ENST00000562833**	ENSG00000260836	15	82536787	82571741	-	protein_coding	Yes	-	1.0013	6.3112	14.6221	1.3E-04	0.0390
**ENST00000373347**	ENSG00000116544	1	34865435	34929650	DLGAP3	protein_coding	Yes	NM_001080418.3	3.4517	-2.2206	14.5891	1.3E-04	0.0392
**ENST00000315677**	ENSG00000170579	18	3496031	4455307	DLGAP1	protein_coding	Yes	NM_004746.4	3.3637	-2.5741	14.5531	1.4E-04	0.0395
**ENST00000381605**	ENSG00000101307	20	1561384	1620009	SIRPB1	protein_coding	Yes	NM_006065.5	-1.0548	2.8565	14.5323	1.4E-04	0.0396
**ENST00000396373**	ENSG00000139083	12	11649673	11895377	ETV6	protein_coding	Yes	NM_001987.5	-1.1339	2.3243	14.5288	1.4E-04	0.0396

**ENST00000325351**	ENSG00000179922	19	55620740	55624566	ZNF784	protein_coding	Yes	NM_203374.2	1.1093	-0.7081	14.5102	1.4E-04	0.0397
**ENST00000301908**	ENSG00000168081	8	28317267	28343351	PNOC	protein_coding	Yes	NM_006228.5	1.7038	-2.8535	14.4397	1.4E-04	0.0402
**ENST00000311308**	ENSG00000174521	19	40216057	40218384	TTC9B	protein_coding	Yes	NM_152479.6	7.0569	-4.2983	14.4331	1.5E-04	0.0402
**ENST00000376708**	ENSG00000204612	9	77019654	77020953	FOXB2	protein_coding	Yes	NM_001013735.1	7.1444	-4.2308	14.4273	1.5E-04	0.0402
**ENST00000466086**	ENSG00000234975	1	228687414	228687826	FTH1P2	processed_pseudogene	Yes	-	-1.4286	-1.4562	14.4360	1.5E-04	0.0402
**ENST00000335624**	ENSG00000186806	19	51331540	51342139	VSIG10L	protein_coding	Yes	NM_001163922.3	3.6746	-2.9303	14.4096	1.5E-04	0.0403
**ENST00000442466**	ENSG00000162006	16	769427	783370	MSLNL	protein_coding	Yes	-	6.0829	-2.6633	14.3965	1.5E-04	0.0405
**ENST00000313683**	ENSG00000182013	19	46466502	46471563	PNMA8A	protein_coding	Yes	NM_018215.4	3.9756	-2.9852	14.3406	1.5E-04	0.0407
**ENST00000329492**	ENSG00000008735	22	50600792	50613978	MAPK8IP2	protein_coding	Yes	NM_012324.6	4.6144	-1.7510	14.3390	1.5E-04	0.0407
**ENST00000510714**	ENSG00000188242	5	466123	472876	PP7080	lncRNA	Yes	-	2.1125	-2.8206	14.3505	1.5E-04	0.0407
**ENST00000215530**	ENSG00000070388	19	639878	644373	FGF22	protein_coding	Yes	NM_020637.2	4.0690	-2.5465	14.3105	1.5E-04	0.0409
**ENST00000254695**	ENSG00000132359	17	2796437	3037741	RAP1GAP2	protein_coding	Yes	NM_015085.5	-1.2931	2.9752	14.3026	1.6E-04	0.0409
**ENST00000251973**	ENSG00000100065	22	37490361	37519415	CARD10	protein_coding	Yes	NM_014550.4	4.5871	-2.7117	14.2744	1.6E-04	0.0409
**ENST00000431719**	ENSG00000235368	7	57424502	57425676	SAPCD2P2	processed_pseudogene	Yes	-	5.2424	-3.4332	14.2748	1.6E-04	0.0409
**ENST00000614247**	ENSG00000277157	6	26188709	26189112	H4C4	protein_coding	Yes	NM_003539.4	1.3037	2.0731	14.2889	1.6E-04	0.0409
**ENST00000650847**	ENSG00000124302	19	33621952	33773506	CHST8	protein_coding	Yes	NM_001127895.2	3.3683	-2.6569	14.2876	1.6E-04	0.0409
**ENST00000460917**	ENSG00000239516	3	180827976	180829850	FLYWCH1P1	processed_pseudogene	Yes	-	7.0178	-4.3282	14.2610	1.6E-04	0.0410
**ENST00000624318**	ENSG00000279476	16	81517823	81520617	-	TEC	Yes	-	-1.9640	0.3321	14.2313	1.6E-04	0.0415
**ENST00000501122**	ENSG00000245532	11	65422797	65445540	NEAT1	lncRNA	Yes	-	-1.3141	9.7007	14.2015	1.6E-04	0.0417
**ENST00000607928**	ENSG00000273456	2	202374768	202376142	-	lncRNA	Yes	-	1.5857	-2.5264	14.1689	1.7E-04	0.0421
**ENST00000505325**	ENSG00000251682	5	61347125	61347716	-	processed_pseudogene	Yes	-	-1.3745	-0.5111	14.1463	1.7E-04	0.0422
**ENST00000473944**	ENSG00000232871	19	48680080	48681020	SEC1P	transcribed_unitary_pseudogene	Yes	-	5.1863	-4.1032	14.1213	1.7E-04	0.0424
**ENST00000337231**	ENSG00000109089	17	74987631	75005800	CDR2L	protein_coding	Yes	NM_014603.3	3.7147	-2.4998	14.1158	1.7E-04	0.0424
**ENST00000371126**	ENSG00000198889	X	126549382	126552814	DCAF12L1	protein_coding	Yes	NM_178470.5	7.5061	-3.9267	14.1039	1.7E-04	0.0424

**ENST00000449590**	ENSG00000160801	3	46877720	46903799	PTH1R	protein_coding	Yes	NM_000316.3	7.0638	-4.2935	14.0761	1.8E-04	0.0426
**ENST00000455617**	ENSG00000229492	22	17418696	17419828	-	processed_pseudogene	Yes	-	6.9743	-4.3610	14.0741	1.8E-04	0.0426
**ENST00000499521**	ENSG00000230551	5	149494313	149504670	-	lncRNA	Yes	-	-1.1302	3.6168	14.0555	1.8E-04	0.0428
**ENST00000397195**	ENSG00000007168	17	2593653	2685615	PAFAH1B1	protein_coding	Yes	NM_000430.4	-1.1356	3.8099	14.0472	1.8E-04	0.0429
**ENST00000616016**	ENSG00000187634	1	923922	944574	SAMD11	protein_coding	Yes	NM_001385641.1	3.9906	-2.0334	14.0283	1.8E-04	0.0431
**ENST00000377898**	ENSG00000173673	1	6244178	6245578	HES3	protein_coding	Yes	NM_001024598.4	6.8151	-4.4608	14.0063	1.8E-04	0.0432
**ENST00000314672**	ENSG00000173826	17	63523357	63546727	KCNH6	protein_coding	Yes	NM_001278919.2	7.1910	-4.2073	14.0006	1.8E-04	0.0432
**ENST00000612928**	ENSG00000275305	20	30816155	30816274	RNA5SP528	rRNA	Yes	-	6.5487	-3.6408	13.9991	1.8E-04	0.0432
**ENST00000634862**	ENSG00000290937	3	198167738	198222513	-	lncRNA	Yes	-	-1.7778	1.7015	13.9813	1.8E-04	0.0434
**ENST00000407595**	ENSG00000055813	2	56183989	56386172	CCDC85A	protein_coding	Yes	NM_001080433.2	4.1485	-3.1663	13.9691	1.9E-04	0.0435
**ENST00000338033**	ENSG00000181085	8	143716348	143722458	MAPK15	protein_coding	Yes	NM_139021.3	7.8584	-3.6383	13.9571	1.9E-04	0.0436
**ENST00000628401**	ENSG00000281183	15	73567011	73569294	NPTN-IT1	lncRNA	Yes	-	-1.1624	-0.7371	13.9558	1.9E-04	0.0436
**ENST00000448869**	ENSG00000229953	1	156646506	156661424	-	lncRNA	Yes	-	6.6002	-4.5876	13.9507	1.9E-04	0.0437
**ENST00000296452**	ENSG00000164061	3	49554476	49671549	BSN	protein_coding	Yes	NM_003458.4	4.2923	-2.5141	13.8740	2.0E-04	0.0438
**ENST00000329198**	ENSG00000183072	5	173232108	173235206	NKX2-5	protein_coding	Yes	NM_004387.4	6.4096	-3.7088	13.8904	1.9E-04	0.0438
**ENST00000334928**	ENSG00000179168	19	38384266	38388034	GGN	protein_coding	Yes	NM_152657.4	4.9952	-2.9258	13.8513	2.0E-04	0.0438
**ENST00000350889**	ENSG00000015592	8	27235307	27258404	STMN4	protein_coding	Yes	NM_030795.4	6.1651	-4.8540	13.9095	1.9E-04	0.0438
**ENST00000371390**	ENSG00000203987	9	137867924	137892570	-	lncRNA	Yes	-	6.8021	-4.4780	13.8517	2.0E-04	0.0438
**ENST00000446477**	ENSG00000224940	7	128350325	128361685	PRRT4	protein_coding	Yes	-	3.9071	-2.5262	13.9042	1.9E-04	0.0438
**ENST00000591533**	ENSG00000267124	19	305572	306467	-	lncRNA	Yes	-	7.1634	-4.2307	13.8708	2.0E-04	0.0438
**ENST00000665650**	ENSG00000227373	1	174110267	174159334	RABGAP1L-DT	lncRNA	Yes	-	1.5556	-2.2406	13.9098	1.9E-04	0.0438
**ENST00000677665**	ENSG00000288649	20	33666942	33668525	ACTL10	protein_coding	Yes	NM_001024675.2	1.0200	-1.1271	13.8774	2.0E-04	0.0438
**ENST00000685045**	ENSG00000289017	15	92908003	92909131	-	lncRNA	Yes	-	-1.4502	-0.4710	13.8538	2.0E-04	0.0438
**ENST00000692682**	ENSG00000289082	2	42795325	42796750	-	lncRNA	Yes	-	-2.3796	-2.4201	13.8378	2.0E-04	0.0438
**ENST00000378247**	ENSG00000204991	16	89828474	89871319	SPIRE2	protein_coding	Yes	NM_032451.2	3.7181	-2.3575	13.8260	2.0E-04	0.0439

**ENST00000504911**	ENSG00000250105	11	66558865	66560384	-	lncRNA	Yes	-	6.3896	-4.7576	13.8111	2.0E-04	0.0440
**ENST00000602669**	ENSG00000269940	14	103694559	103695170	-	lncRNA	Yes	-	-1.9785	-1.1206	13.7897	2.0E-04	0.0442
**ENST00000215368**	ENSG00000099617	19	1285872	1301431	EFNA2	protein_coding	Yes	NM_001405.4	5.5300	-2.8736	13.6609	2.2E-04	0.0443
**ENST00000256646**	ENSG00000134250	1	119911552	120069662	NOTCH2	protein_coding	Yes	NM_024408.4	-1.1191	5.7614	13.7110	2.1E-04	0.0443
**ENST00000257572**	ENSG00000135116	12	116856143	116881441	HRK	protein_coding	Yes	NM_003806.4	1.7705	-2.2394	13.6468	2.2E-04	0.0443
**ENST00000289957**	ENSG00000147432	8	42697365	42737407	CHRNB3	protein_coding	Yes	NM_000749.5	6.4922	-4.7039	13.7306	2.1E-04	0.0443
**ENST00000306318**	ENSG00000168505	2	236165235	236168386	GBX2	protein_coding	Yes	NM_001485.4	6.1494	-3.9600	13.6947	2.2E-04	0.0443
**ENST00000315489**	ENSG00000181781	19	463360	474983	ODF3L2	protein_coding	Yes	NM_182577.3	6.2352	-3.2986	13.6929	2.2E-04	0.0443
**ENST00000363593**	ENSG00000200463	17	8173453	8173587	SNORD118	snoRNA	Yes	-	-3.2287	-3.9198	13.6237	2.2E-04	0.0443
**ENST00000414273**	ENSG00000237973	1	631073	632616	MTCO1P12	unprocessed_pseudogene	Yes	-	-1.1191	1.9631	13.6668	2.2E-04	0.0443
**ENST00000426928**	ENSG00000215906	1	22953042	22972866	LACTBL1	protein_coding	Yes	NM_001289974.2	6.2848	-3.2549	13.6151	2.2E-04	0.0443
**ENST00000496058**	ENSG00000240669	4	152613874	152614333	-	processed_pseudogene	Yes	-	-1.5568	-1.7093	13.6513	2.2E-04	0.0443
**ENST00000507448**	ENSG00000251535	4	336257	336786	-	processed_pseudogene	Yes	-	3.3151	-3.2480	13.6277	2.2E-04	0.0443
**ENST00000515156**	ENSG00000250138	5	69631962	69636399	-	unprocessed_pseudogene	Yes	-	-2.9649	-0.7610	13.6823	2.2E-04	0.0443
**ENST00000578539**	ENSG00000264853	17	75370946	75373736	-	lncRNA	Yes	-	-1.7069	0.9083	13.7154	2.1E-04	0.0443
**ENST00000611923**	ENSG00000278713	16	29862759	29863417	-	lncRNA	Yes	-	1.2332	-0.8863	13.6154	2.2E-04	0.0443
**ENST00000615164**	ENSG00000276966	6	26204609	26205021	H4C5	protein_coding	Yes	NM_003545.4	1.1252	3.8334	13.7307	2.1E-04	0.0443
**ENST00000651351**	ENSG00000197859	9	133534703	133575519	ADAMTSL2	protein_coding	Yes	NM_014694.4	4.8593	-2.6617	13.6821	2.2E-04	0.0443
**ENST00000661319**	ENSG00000287356	1	2315039	2323085	-	lncRNA	Yes	-	4.6529	-1.9404	13.7397	2.1E-04	0.0443
**ENST00000668204**	ENSG00000266401	18	3653029	3656269	-	lncRNA	Yes	-	1.2494	-0.8234	13.6175	2.2E-04	0.0443
**ENST00000688494**	ENSG00000289236	20	62120045	62121762	-	lncRNA	Yes	-	5.3619	-4.4893	13.6189	2.2E-04	0.0443
**ENST00000690203**	ENSG00000015413	16	89613641	89638433	DPEP1	protein_coding	Yes	NM_001389466.1	7.1130	-4.2696	13.6661	2.2E-04	0.0443
**ENST00000695704**	ENSG00000289691	7	100397572	100420722	-	lncRNA	Yes	-	-1.2968	-0.4996	13.6085	2.3E-04	0.0444

**ENST00000378733**	ENSG00000205090	1	1534777	1540624	TMEM240	protein_coding	Yes	NM_001114748.2	3.9921	-2.5735	13.6006	2.3E-04	0.0444
**ENST00000554623**	ENSG00000259006	16	89919826	89922662	-	lncRNA	Yes	-	5.4676	-5.1526	13.5938	2.3E-04	0.0445
**ENST00000322989**	ENSG00000134419	16	18781294	18790334	RPS15A	protein_coding	Yes	NM_001019.5	1.0718	6.4543	13.5832	2.3E-04	0.0445
**ENST00000623674**	ENSG00000279166	2	144494264	144496878	-	TEC	Yes	-	-1.0600	2.7526	13.5757	2.3E-04	0.0445
**ENST00000490496**	ENSG00000240767	13	46169457	46169748	RN7SL288P	misc_RNA	Yes	-	-1.5100	-0.9800	13.5447	2.3E-04	0.0447
**ENST00000361066**	ENSG00000134202	1	109733936	109741038	GSTM3	protein_coding	Yes	NM_000849.5	2.0635	-0.9774	13.5154	2.4E-04	0.0450
**ENST00000338222**	ENSG00000188021	X	56563626	56567868	UBQLN2	protein_coding	Yes	NM_013444.4	-1.1859	2.0780	13.5065	2.4E-04	0.0450
**ENST00000341735**	ENSG00000188095	15	89776331	89778754	MESP2	protein_coding	Yes	NM_001039958.2	5.3605	-4.0332	13.4868	2.4E-04	0.0451
**ENST00000441346**	ENSG00000234073	3	36880183	36880729	-	processed_pseudogene	Yes	-	-1.0411	-0.7122	13.4915	2.4E-04	0.0451
**ENST00000261980**	ENSG00000119614	14	74239448	74262738	VSX2	protein_coding	Yes	NM_182894.3	6.5086	-4.6551	13.4741	2.4E-04	0.0452
**ENST00000274721**	ENSG00000146013	5	138252379	138274621	GFRA3	protein_coding	Yes	NM_001496.4	4.6510	-4.1906	13.4650	2.4E-04	0.0454
**ENST00000304477**	ENSG00000171794	10	133230216	133231558	UTF1	protein_coding	Yes	NM_003577.3	4.9422	-2.9343	13.4301	2.5E-04	0.0457
**ENST00000397542**	ENSG00000099834	11	616582	624955	CDHR5	protein_coding	Yes	NM_021924.5	4.8970	-2.6130	13.4256	2.5E-04	0.0457
**ENST00000585937**	ENSG00000267059	19	1578338	1605445	-	protein_coding	Yes	-	1.3613	3.1224	13.4052	2.5E-04	0.0458
**ENST00000332235**	ENSG00000183186	19	405444	409147	C2CD4C	protein_coding	Yes	NM_001136263.2	5.9563	-2.0703	13.3734	2.6E-04	0.0460
**ENST00000420428**	ENSG00000101489	18	37243039	37565798	CELF4	protein_coding	Yes	NM_020180.4	4.8175	-3.1533	13.3627	2.6E-04	0.0461
**ENST00000356613**	ENSG00000198576	8	142611048	142614479	ARC	protein_coding	Yes	NM_015193.5	5.7713	-3.2566	13.3296	2.6E-04	0.0463
**ENST00000582106**	ENSG00000265688	17	81927828	81930753	MILIP	lncRNA	Yes	-	6.3909	-3.7808	13.3350	2.6E-04	0.0463
**ENST00000501931**	ENSG00000246877	15	75727669	75738623	DNM1P35	lncRNA	Yes	-	6.4557	-4.6880	13.3077	2.6E-04	0.0464
**ENST00000326586**	ENSG00000181524	6	42956344	42956765	RPL24P4	processed_pseudogene	Yes	-	1.1044	4.0914	13.3022	2.7E-04	0.0464
**ENST00000349945**	ENSG00000102908	16	69565965	69704654	NFAT5	protein_coding	Yes	NM_138713.4	-1.1531	4.4209	13.2899	2.7E-04	0.0465
**ENST00000421673**	ENSG00000184956	11	1012822	1036718	MUC6	protein_coding	Yes	NM_005961.3	4.5241	-1.8646	13.2873	2.7E-04	0.0466
**ENST00000327134**	ENSG00000180370	3	196739856	196832647	PAK2	protein_coding	Yes	NM_002577.4	-1.1471	5.0063	13.2582	2.7E-04	0.0468
**ENST00000459460**	ENSG00000238829	2	201141903	201141966	RNU7-45P	snRNA	Yes	-	-2.1940	-2.2010	13.2468	2.7E-04	0.0468
**ENST00000368586**	ENSG00000171811	10	132808391	132942570	CFAP46	protein_coding	Yes	NM_001200049.3	4.2717	-2.0462	13.2401	2.7E-04	0.0469

**ENST00000683046**	ENSG00000186314	5	145756343	145835342	PRELID2	protein_coding	Yes	NM_205846.3	1.0246	0.8431	13.2347	2.7E-04	0.0470
**ENST00000396872**	ENSG00000164638	7	5282942	5306912	SLC29A4	protein_coding	Yes	NM_153247.4	3.7241	-2.9483	13.2308	2.8E-04	0.0470
**ENST00000328278**	ENSG00000185028	5	191494	196334	LRRC14B	protein_coding	Yes	NM_001080478.3	5.2889	-3.1782	13.2156	2.8E-04	0.0472
**ENST00000329267**	ENSG00000185198	19	685545	695452	PRSS57	protein_coding	Yes	NM_001308209.2	2.1381	-2.2760	13.2003	2.8E-04	0.0472
**ENST00000434752**	ENSG00000228672	5	139390591	139395104	PROB1	protein_coding	Yes	NM_001161546.2	3.2989	-2.2055	13.1706	2.8E-04	0.0474
**ENST00000215637**	ENSG00000099866	19	496485	505343	MADCAM1	protein_coding	Yes	NM_130760.3	4.5021	-2.8762	13.1202	2.9E-04	0.0475
**ENST00000270631**	ENSG00000142538	19	49422418	49423441	PTH2	protein_coding	Yes	NM_178449.4	6.9876	-4.3673	13.1432	2.9E-04	0.0475
**ENST00000507617**	ENSG00000248785	4	109673842	109674124	HIGD1AP14	processed_pseudogene	Yes	-	-2.1127	-2.6136	13.1235	2.9E-04	0.0475
**ENST00000242317**	ENSG00000122735	9	34458804	34520984	DNAI1	protein_coding	Yes	NM_012144.4	6.2889	-4.8302	13.1158	2.9E-04	0.0476
**ENST00000488441**	ENSG00000232888	12	132825700	132826184	RPS11P5	processed_pseudogene	Yes	-	1.0573	1.7645	13.1056	2.9E-04	0.0477
**ENST00000275560**	ENSG00000146700	7	76389336	76409697	SSC4D	protein_coding	Yes	NM_080744.2	4.7735	-3.3774	13.0477	3.0E-04	0.0483
**ENST00000492974**	ENSG00000218426	6	153282286	153282733	-	processed_pseudogene	Yes	-	1.0446	5.5892	13.0480	3.0E-04	0.0483
**ENST00000415014**	ENSG00000234402	14	105546609	105549694	ELK2BP	unprocessed_pseudogene	Yes	-	6.1493	-3.3809	13.0196	3.1E-04	0.0485
**ENST00000377670**	ENSG00000124440	19	46297041	46343433	HIF3A	protein_coding	Yes	NM_152795.4	6.9589	-4.3905	13.0132	3.1E-04	0.0486
**ENST00000567091**	ENSG00000260394	16	678503	679777	STUB1-DT	lncRNA	Yes	-	6.0241	-4.9681	12.9921	3.1E-04	0.0488
**ENST00000580341**	ENSG00000277452	1	150566561	150566860	RN7SL473P	misc_RNA	Yes	-	-1.7685	-0.6526	12.9920	3.1E-04	0.0488
**ENST00000378295**	ENSG00000078900	1	3652515	3736201	TP73	protein_coding	Yes	NM_005427.4	3.6358	-2.9634	12.9788	3.2E-04	0.0489
**ENST00000580891**	ENSG00000266805	18	9506241	9509726	-	lncRNA	Yes	-	-1.7307	-1.2512	12.9809	3.1E-04	0.0489
**ENST00000691244**	ENSG00000225127	20	21085577	21106358	LINC00237	lncRNA	Yes	-	5.3112	-5.2276	12.9811	3.1E-04	0.0489
**ENST00000287038**	ENSG00000156482	8	98041720	98045545	RPL30	protein_coding	Yes	NM_000989.4	1.0108	6.7577	12.9499	3.2E-04	0.0490
**ENST00000347132**	ENSG00000117013	1	40783786	40840452	KCNQ4	protein_coding	Yes	NM_004700.4	4.4556	-2.9524	12.9509	3.2E-04	0.0490
**ENST00000391553**	ENSG00000212864	9	137220258	137222240	RNF208	protein_coding	Yes	NM_031297.7	4.0656	-3.1131	12.9540	3.2E-04	0.0490
**ENST00000538872**	ENSG00000120645	12	66766	178455	IQSEC3	protein_coding	Yes	NM_001170738.2	4.3672	-1.7495	12.9406	3.2E-04	0.0490
**ENST00000591899**	ENSG00000127540	19	1597168	1605462	UQCR11	protein_coding	Yes	NM_006830.4	1.3638	3.1716	12.9366	3.2E-04	0.0490

**ENST00000624790**	ENSG00000280414	2	174334437	174337566	-	TEC	Yes	-	5.6425	-3.0477	12.9567	3.2E-04	0.0490
**ENST00000692767**	ENSG00000289281	16	3004131	3004859	-	protein_coding	Yes	-	4.8527	-4.7456	12.9459	3.2E-04	0.0490
**ENST00000160298**	ENSG00000076826	19	7595862	7618304	CAMSAP3	protein_coding	Yes	NM_020902.2	3.8807	-2.0316	12.8443	3.4E-04	0.0491
**ENST00000256797**	ENSG00000134398	16	23690309	23713222	ERN2	protein_coding	Yes	NM_033266.4	6.5340	-4.6591	12.8981	3.3E-04	0.0491
**ENST00000312541**	ENSG00000112763	6	26457954	26469637	BTN2A1	protein_coding	Yes	NM_007049.5	-1.0411	2.3355	12.8822	3.3E-04	0.0491
**ENST00000325602**	ENSG00000181631	3	151326311	151329549	P2RY13	protein_coding	Yes	NM_176894.3	-1.0185	3.2301	12.8472	3.4E-04	0.0491
**ENST00000340083**	ENSG00000175920	4	3463305	3494482	DOK7	protein_coding	Yes	NM_173660.5	2.2787	-2.2399	12.8618	3.4E-04	0.0491
**ENST00000371561**	ENSG00000176884	9	137139153	137168756	GRIN1	protein_coding	Yes	NM_007327.4	5.0076	-2.0396	12.8434	3.4E-04	0.0491
**ENST00000377574**	ENSG00000197891	11	64591219	64602344	SLC22A12	protein_coding	Yes	NM_144585.4	6.6545	-4.5808	12.8274	3.4E-04	0.0491
**ENST00000578387**	ENSG00000251359	4	183097016	183099199	WWC2-AS2	lncRNA	Yes	-	4.4365	-3.4473	12.9067	3.3E-04	0.0491
**ENST00000618550**	ENSG00000183248	19	7868718	7874390	PRR36	protein_coding	Yes	NM_001190467.2	4.6742	-2.5161	12.8868	3.3E-04	0.0491
**ENST00000624840**	ENSG00000280205	18	76385641	76389406	-	TEC	Yes	-	-1.5439	-1.1003	12.8934	3.3E-04	0.0491
**ENST00000652299**	ENSG00000052850	11	44260439	44310139	ALX4	protein_coding	Yes	NM_021926.4	5.0031	-3.3449	12.8662	3.3E-04	0.0491
**ENST00000685067**	ENSG00000288805	6	30460824	30481634	-	lncRNA	Yes	-	5.9368	-4.1160	12.8529	3.4E-04	0.0491
**ENST00000423539**	ENSG00000228079	2	64086352	64088246	-	lncRNA	Yes	-	-1.1607	-1.2114	12.8237	3.4E-04	0.0491
**ENST00000599174**	ENSG00000267909	14	69569798	69574871	CCDC177	protein_coding	Yes	NM_001271507.2	5.4786	-3.1640	12.8210	3.4E-04	0.0491
**ENST00000230050**	ENSG00000112306	6	132814568	132817564	RPS12	protein_coding	Yes	NM_001016.4	1.0475	6.8175	12.7656	3.5E-04	0.0494
**ENST00000265459**	ENSG00000110076	11	64606173	64723197	NRXN2	protein_coding	Yes	NM_015080.4	4.0643	-1.7282	12.7797	3.5E-04	0.0494
**ENST00000297477**	ENSG00000164855	7	1542234	1556205	TMEM184A	protein_coding	Yes	NM_001097620.2	3.0622	-1.9393	12.7746	3.5E-04	0.0494
**ENST00000310597**	ENSG00000174276	11	65116402	65117701	ZNHIT2	protein_coding	Yes	NM_014205.4	1.0168	-0.7119	12.7425	3.6E-04	0.0494
**ENST00000323249**	ENSG00000131188	5	177446813	177456286	PRR7	protein_coding	Yes	NM_030567.5	1.2692	-1.1115	12.7389	3.6E-04	0.0494
**ENST00000366273**	ENSG00000213073	6	160093422	160093993	CHP1P2	transcribed_processed_pseudogene	Yes	-	-1.1915	-0.8937	12.7528	3.6E-04	0.0494
**ENST00000404795**	ENSG00000142700	1	50417549	50423443	DMRTA2	protein_coding	Yes	NM_032110.3	5.5896	-3.0731	12.7690	3.5E-04	0.0494
**ENST00000501387**	ENSG00000247131	12	69713632	69738574	-	lncRNA	Yes	-	6.7706	-4.5187	12.7435	3.6E-04	0.0494
**ENST00000515384**	ENSG00000165566	13	25161678	25172288	AMER2	protein_coding	Yes	NM_152704.4	5.4188	-3.5304	12.7840	3.5E-04	0.0494

**ENST00000624428**	ENSG00000279884	2	174545384	174546335	-	TEC	Yes	-	-1.2380	0.5582	12.7353	3.6E-04	0.0494
**ENST00000625598**	ENSG00000280614	21	8393418	8394341	-	lncRNA	Yes	-	4.3038	-3.1380	12.7443	3.6E-04	0.0494
**ENST00000369939**	ENSG00000116299	1	109114114	109206781	ELAPOR1	protein_coding	Yes	NM_020775.5	-1.4552	2.5765	12.7266	3.6E-04	0.0495
**ENST00000537762**	ENSG00000256542	12	132277348	132280900	-	lncRNA	Yes	-	6.5651	-4.6489	12.7239	3.6E-04	0.0495
**ENST00000263100**	ENSG00000121410	19	58345182	58353492	A1BG	protein_coding	Yes	NM_130786.4	1.3405	-0.9229	12.7124	3.6E-04	0.0497
**ENST00000294288**	ENSG00000167791	11	67518911	67523446	CABP2	protein_coding	Yes	NM_016366.3	6.2530	-4.8080	12.7130	3.6E-04	0.0497
**ENST00000689091**	ENSG00000289097	6	18277241	18278064	-	lncRNA	Yes	-	6.1562	-4.9093	12.7099	3.6E-04	0.0497
**ENST00000685999**	ENSG00000289144	21	44748899	44750986	-	lncRNA	Yes	-	7.0143	-4.3550	12.7002	3.7E-04	0.0497
**ENST00000288502**	ENSG00000157693	9	114611290	114646422	TMEM268	protein_coding	Yes	NM_153045.4	-1.1230	0.7162	12.6894	3.7E-04	0.0498
**ENST00000263266**	ENSG00000105523	19	48599960	48615076	FAM83E	protein_coding	Yes	NM_017708.4	2.7091	-2.4531	12.6402	3.8E-04	0.0500
**ENST00000441146**	ENSG00000229331	X	30671634	30672166	GK-IT1	lncRNA	Yes	-	-3.1284	-2.7388	12.6458	3.8E-04	0.0500
**ENST00000539008**	ENSG00000011347	11	61513713	61581076	SYT7	protein_coding	Yes	NM_001365809.2	5.1394	-3.0423	12.6654	3.7E-04	0.0500
**ENST00000641816**	ENSG00000284638	19	3482109	3483441	SMIM44	protein_coding	Yes	NM_001395954.1	6.6603	-4.5951	12.6511	3.8E-04	0.0500

Supplementary table 5: Differentially expressed genes (Female MwA patients vs. Female controls). Log_2FC – Log 2-fold change; logCPM – log counts per million; LR – likelihood ratio; FDR – False discovery rate

Transcript	Transcript stable ID	chr	start	end	Gene name	Gene type	Canonical	MANE Select	log_2FC	logCPM	LR	P-Value	FDR
**ENST00000296591**	ENSG00000164176	5	83940553	84384880	EDIL3	protein_coding	Yes	NM_005711.5	3.1140	-3.3254	27.2379	1.80E-07	0.0047
**ENST00000450128**	ENSG00000227262	6	29925982	29926973	HCG4B	unprocessed_pseudogene	Yes	-	3.2011	-2.0265	28.1148	1.14E-07	0.0047
**ENST00000689584**	ENSG00000181218	1	2.28E+08	2.28E+08	H2AW	protein_coding	Yes	NM_033445.3	1.5050	-0.1503	27.6481	1.46E-07	0.0047
**ENST00000357045**	ENSG00000237655	2	1.78E+08	1.78E+08	-	lncRNA	Yes	-	5.2782	-5.1531	26.2771	2.96E-07	0.0049
**ENST00000682961**	ENSG00000165181	9	1.12E+08	1.12E+08	SHOC1	protein_coding	Yes	NM_001378211.1	-2.4328	-2.8258	26.2156	3.05E-07	0.0049
**ENST00000663752**	ENSG00000286489	10	1.02E+08	1.02E+08	-	lncRNA	Yes	-	2.1383	-3.3946	25.0513	5.58E-07	0.0066
**ENST00000700791**	ENSG00000289829	6	31826840	31827443	-	lncRNA	Yes	-	2.5207	-3.7680	24.9331	5.94E-07	0.0066
**ENST00000374940**	ENSG00000237541	6	32741390	32747198	HLA-DQA2	protein_coding	Yes	NM_020056.5	-3.9079	1.1417	22.4323	2.18E-06	0.0169
**ENST00000524091**	ENSG00000253570	8	38600660	38601200	RNF5P1	processed_pseudogene	Yes	-	1.2705	0.0280	22.0562	2.65E-06	0.0193

**ENST00000376008**	ENSG00000171729	1	15153746	15220478	TMEM51	protein_coding	Yes	NM_001136218.2	-2.5996	-3.1364	21.4485	3.63E-06	0.0246
**ENST00000458797**	ENSG00000238741	3	1.61E+08	1.61E+08	SCARNA7	scaRNA	Yes	-	1.3890	5.1216	20.7067	5.35E-06	0.0302
**ENST00000495392**	ENSG00000227081	12	3211662	3211917	-	processed_ pseudogene	Yes	-	1.7130	6.5123	20.6754	5.44E-06	0.0302
**ENST00000547512**	ENSG00000257246	12	51124627	51125381	PHB1P19	transcribed_ processed_ pseudogene	Yes	-	-2.6227	-2.1427	20.5527	5.80E-06	0.0302
**ENST00000245458**	ENSG00000213741	14	49583576	49586380	RPS29	protein_coding	Yes	NM_001032.5	1.0391	4.7245	20.3882	6.32E-06	0.0304
**ENST00000614247**	ENSG00000277157	6	26188709	26189112	H4C4	protein_coding	Yes	NM_003539.4	1.3080	2.0684	20.2971	6.63E-06	0.0310
**ENST00000590850**	ENSG00000267637	17	59784812	59785035	-	lncRNA	Yes	-	-2.1735	-2.0782	19.9854	7.80E-06	0.0339
**ENST00000244537**	ENSG00000274618	6	26240392	26240793	H4C6	protein_coding	Yes	NM_003540.4	1.1053	2.7263	18.6108	1.60E-05	0.0385
**ENST00000335750**	ENSG00000021645	14	78170372	79868291	NRXN3	protein_coding	Yes	NM_001330195.2	1.8072	-0.1518	19.2987	1.12E-05	0.0385
**ENST00000364711**	ENSG00000201581	10	6149622	6149940	RN7SKP78	misc_RNA	Yes	-	-3.9214	-4.3764	19.4074	1.06E-05	0.0385
**ENST00000407942**	ENSG00000217643	2	25822468	25822950	PTGES3P2	processed_ pseudogene	Yes	-	-2.7661	-4.5385	18.9455	1.35E-05	0.0385
**ENST00000433113**	ENSG00000240527	10	95833507	95873758	-	lncRNA	Yes	-	-1.6258	-0.9131	19.0641	1.26E-05	0.0385
**ENST00000615164**	ENSG00000276966	6	26204609	26205021	H4C5	protein_coding	Yes	NM_003545.4	1.0218	3.7652	18.6825	1.54E-05	0.0385
**ENST00000615353**	ENSG00000276180	6	27139281	27139678	H4C9	protein_coding	Yes	NM_003495.3	1.0731	1.2489	18.6597	1.56E-05	0.0385
**ENST00000618305**	ENSG00000275126	6	27873147	27873534	H4C13	protein_coding	Yes	NM_003546.3	1.1923	0.7779	19.2954	1.12E-05	0.0385
**ENST00000009180**	ENSG00000010278	12	6200399	6238266	CD9	protein_coding	Yes	NM_001769.4	1.0967	2.0175	18.3951	1.80E-05	0.0390
**ENST00000602658**	ENSG00000227888	8	12362381	12388296	FAM66A	lncRNA	Yes	-	2.2528	-4.3648	17.5568	2.79E-05	0.0489
**ENST00000591899**	ENSG00000127540	19	1597168	1605462	UQCR11	protein_coding	Yes	NM_006830.4	1.2206	3.0743	17.4799	2.90E-05	0.049625

Supplementary table 6: Differentially expressed genes (Male MwA patients vs. Male controls). Log_2FC – Log 2-fold change; logCPM – log counts per million; LR – likelihood ratio; FDR – False discovery rate

Transcript	Transcript stable ID	chr	start	end	Gene name	Gene type	Canonical	MANE Select	log_2FC	logCPM	LR	P-Value	FDR
**ENST00000278865**	ENSG00000149516	11	60056664	60071115	MS4A3	protein_coding	Yes	NM_006138.5	2.1817	1.1389	46.9482	7.29E-12	2.7E-07
**ENST00000341495**	ENSG00000188056	6	41228348	41238882	TREML4	protein_coding	Yes	NM_198153.3	4.3499	-2.2702	44.7067	2.29E-11	5.6E-07
**ENST00000347310**	ENSG00000162594	1	67166453	67259979	IL23R	protein_coding	Yes	NM_144701.3	2.8983	-2.1992	43.9709	3.33E-11	7.0E-07
**ENST00000273352**	ENSG00000144820	3	100609600	100695479	ADGRG7	protein_coding	Yes	NM_032787.3	7.8590	-1.0806	40.5779	1.89E-10	2.3E-06

**ENST00000219022**	ENSG00000102837	13	53028812	53052057	OLFM4	protein_coding	Yes	NM_006418.5	4.9038	2.0702	38.1142	6.67E-10	6.8E-06
**ENST00000685869**	ENSG00000289459	9	87930404	87939270	-	lncRNA	Yes	-	-6.9362	-1.0117	35.5195	2.53E-09	2.3E-05
**ENST00000371225**	ENSG00000184292	1	58575432	58577252	TACSTD2	protein_coding	Yes	NM_002353.3	2.8798	-1.4909	32.6883	1.08E-08	7.3E-05
**ENST00000604204**	ENSG00000271361	6	3023141	3023772	HTATSF1P2	processed_ pseudogene	Yes	-	-2.4080	0.3831	31.9137	1.61E-08	0.00010
**ENST00000648076**	ENSG00000065618	10	104031285	104085880	COL17A1	protein_coding	Yes	NM_000494.4	2.8016	-1.6714	29.9130	4.52E-08	0.00020
**ENST00000446997**	ENSG00000144290	2	161624415	161985270	SLC4A10	protein_coding	Yes	NM_001178015.2	1.9584	0.5763	25.4332	4.58E-07	0.00142
**ENST00000538924**	ENSG00000068615	2	86213992	86337626	REEP1	protein_coding	Yes	NM_001371279.1	3.6162	-2.2301	24.4026	7.82E-07	0.00216
**ENST00000412472**	ENSG00000206066	22	25318255	25320080	IGLL3P	unprocessed_ pseudogene	Yes	-	7.7216	-3.3280	24.2848	8.31E-07	0.00223
**ENST00000377122**	ENSG00000078114	10	20779972	20897311	NEBL	protein_coding	Yes	NM_006393.3	4.2588	-1.0112	23.9245	1.00E-06	0.00262
**ENST00000435803**	ENSG00000179869	7	48171457	48647497	ABCA13	protein_coding	Yes	NM_152701.5	1.7532	1.9765	23.6731	1.14E-06	0.00291
**ENST00000377424**	ENSG00000142583	1	9035105	9069635	SLC2A5	protein_coding	Yes	NM_003039.3	2.0901	-0.7107	22.9343	1.68E-06	0.00401
**ENST00000242208**	ENSG00000122641	7	41685113	41703090	INHBA	protein_coding	Yes	NM_002192.4	2.3335	-2.3120	22.0363	2.68E-06	0.00577
**ENST00000263045**	ENSG00000096006	6	49727375	49744388	CRISP3	protein_coding	Yes	NM_006061.4	1.8947	0.4257	21.1488	4.25E-06	0.00897
**ENST00000642449**	ENSG00000101425	20	38304155	38337505	BPI	protein_coding	Yes	NM_001725.3	2.1690	-0.0041	20.3295	6.52E-06	0.01322
**ENST00000652295**	ENSG00000164047	3	48223494	48225485	CAMP	protein_coding	Yes	NM_004345.5	2.5014	2.7290	20.3246	6.54E-06	0.01322
**ENST00000295992**	ENSG00000163710	3	142817873	142889083	PCOLCE2	protein_coding	Yes	NM_013363.4	3.0086	-2.7411	19.9661	7.88E-06	0.01532
**ENST00000650541**	ENSG00000227674	13	63851196	64076044	LINC00355	lncRNA	Yes	-	7.4939	-2.8879	19.6022	9.54E-06	0.01817
**ENST00000327857**	ENSG00000239839	8	7015868	7018297	DEFA3	protein_coding	Yes	NM_005217.4	4.0249	4.9061	19.4312	1.04E-05	0.01968
**ENST00000390454**	ENSG00000211806	14	22112346	22113031	TRAV25	TR_V_gene	Yes	-	2.5191	-0.9858	19.1132	1.23E-05	0.0226
**ENST00000309539**	ENSG00000173391	12	10158300	10172132	OLR1	protein_coding	Yes	NM_002543.4	2.4834	-1.4019	18.8866	1.39E-05	0.02498
**ENST00000199764**	ENSG00000086548	19	41755529	41772211	CEACAM6	protein_coding	Yes	NM_002483.7	2.5533	-1.1634	18.6010	1.61E-05	0.02824
**ENST00000390440**	ENSG00000211792	14	21924062	21924651	TRAV14DV4	TR_V_gene	Yes	-	1.5672	-1.0169	18.0386	2.16E-05	0.03631
**ENST00000367434**	ENSG00000162630	1	193178729	193186613	B3GALT2	protein_coding	Yes	NM_003783.3	1.6628	-0.5605	17.3889	3.05E-05	0.04900

**Supplementary table 7:** Differentially expressed genes (Male MwoA patients vs. Male MwA patients). Log_2FC – Log 2-fold change; logCPM – log counts per million; LR – likelihood ratio; FDR – False discovery rate													
**Transcript**	**Transcript stable ID**	**chr**	**start**	**end**	**Gene name**	**Gene type**	**Canonical**	**MANE Select**	**log_2FC**	**logCPM**	**LR**	**P-Value**	**FDR**
**ENST00000604204**	ENSG00000271361	6	3023141	3023772	HTATSF1P2	processed_pseudogene	Yes	-	2.4736	0.4214	42.9100	5.73E-11	1.15E-05
**ENST00000551108**	ENSG00000257599	12	29389293	29487473	OVCH1-AS1	lncRNA	Yes	-	-2.1983	-1.5590	29.1078	6.85E-08	0.001522
**ENST00000273352**	ENSG00000144820	3	100609600	100695479	ADGRG7	protein_coding	Yes	NM_032787.3	-6.5032	-0.8366	27.6923	1.42E-07	0.002788
**ENST00000538924**	ENSG00000068615	2	86213992	86337626	REEP1	protein_coding	Yes	NM_001371279.1	-2.7617	-1.8809	26.3186	2.89E-07	0.004138
**ENST00000377122**	ENSG00000078114	10	20779972	20897311	NEBL	protein_coding	Yes	NM_006393.3	-4.3258	-0.8280	25.7073	3.97E-07	0.004552
**ENST00000412472**	ENSG00000206066	22	25318255	25320080	IGLL3P	unprocessed_pseudogene	Yes	-	-7.5226	-3.1055	25.6495	4.09E-07	0.004552
**ENST00000450893**	ENSG00000234389	2	102438712	102440475	-	lncRNA	Yes	-	1.9615	-0.3694	23.9762	9.75E-07	0.008487
**ENST00000375352**	ENSG00000204345	17	74579364	74592283	CD300LD	protein_coding	Yes	NM_001115152.2	4.8518	-3.0222	22.0614	2.64E-06	0.019575

**Supplementary Table 8:** Gene set enrichment results of Peripheral Blood RNA-seq data of the migraine patients vs. healthy controls comparison. Description: functional terms in the differentially expressed gene list accordingly to the Reactome database. NES – normalized enrichment score; FDR – false discovery rate						
**ID**	**Description**	**Enrichment Score**	**NES**	**p-Value**	**FDR**	**Genes**
R-HSA-1430728	**Metabolism**	0.7302	2.3453	2.2 E-16	2.2 E-16	PRODH;SULT6B1;TYR;UGT1A1;UGT1A4;UGT1A5;UGT1A7;UGT1A8
R-HSA-1280215	**Cytokine Signaling in Immune system**	-0.4892	-2.2921	2.2 E-16	2.2 E-16	CCL2;CXCL10;FCGR1A;GBP1;GBP6;HERC5;IFI6;IFIT1;IFIT2;IFIT3;IRF7;ISG15;MX1;OAS1;OAS2;OAS3;OASL;RSAD2;TRIM6;USP18;XAF1
R-HSA-1169410	**Antiviral mechanism by IFN-stimulated genes**	-0.6662	-2.3357	2.2 E-16	2.2 E-16	HERC5;IFIT1;ISG15;MX1;OAS1;OAS2;OAS3;OASL;USP18
R-HSA-168256	**Immune System**	-0.4738	-2.3664	2.2 E-16	2.2 E-16	CCL2;CD274;CXCL10;FCGR1A;GBP1;GBP6;HERC5;IFI6;IFIT1;IFIT2;IFIT3;IRF7;ISG15;MX1;OAS1;OAS2;OAS3;OASL;PDCD1LG2;RSAD2;SERPING1;SIGLEC1;TNFAIP6;TRIM6;USP18;XAF1
R-HSA-909733	**Interferon alpha/beta signaling**	-0.6825	-2.5159	2.2 E-16	2.2 E-16	IFI6;IFIT1;IFIT2;IFIT3;IRF7;ISG15;MX1;OAS1;OAS2;OAS3;OASL;RSAD2;USP18;XAF1
R-HSA-913531	**Interferon Signaling**	-0.5709	-2.5169	2.2 E-16	2.2 E-16	DDX58;EIF2AK2;FCGR1A;GBP1;GBP5;GBP6;HERC5;IFI6;IFIT1;IFIT2;IFIT3;IRF7;ISG15;MX1;OAS1;OAS2;OAS3;OASL;RSAD2;TRIM6;USP18;XAF1
R-HSA-156588	**Glucuronidation**	0.9006	2.0684	2.2 E-16	2.27E-04	UGT1A1;UGT1A4;UGT1A5;UGT1A7;UGT1A8
R-HSA-156580	**Phase II - Conjugation of compounds**	0.8586	2.2540	2.2 E-16	3.03E-04	SULT6B1;UGT1A1;UGT1A4;UGT1A5;UGT1A7;UGT1A8

R-HSA-211859	**Biological oxidations**	0.8586	2.2540	2.2 E-16	3.03E-04	SULT6B1;UGT1A1;UGT1A4;UGT1A5;UGT1A7;UGT1A8
R-HSA-1169408	**ISG15 antiviral mechanism**	-0.6255	-1.8206	0.0111	0.0210	HERC5;IFIT1;ISG15;MX1;USP18
R-HSA-877300	**Interferon gamma signaling**	-0.5208	-1.7613	0.0190	0.0240	FCGR1A;GBP1;GBP5;GBP6;IRF7;OAS1;OAS2;OAS3;OASL;TRIM6
R-HSA-8983711	OAS antiviral response	-0.6446	-1.5804	0.0306	0.0690	OAS1;OAS2;OAS3;OASL
R-HSA-392499	Metabolism of proteins	-0.5606	-1.5083	0.0660	0.0838	ATF3;CCL2;DDX58;IFIH1;LY6E;USP18
R-HSA-168249	Innate Immune System	-0.4571	-1.4885	0.0857	0.0845	DDX58;FCGR1A;HERC5;IFIH1;IRF7;ISG15;SERPING1;TNFAIP6
R-HSA-1643685	Disease	0.5292	1.2984	0.1784	0.1701	UGT1A1;UGT1A4

Supplementary Table 9: Gene set enrichment results of Peripheral Blood RNA-seq data of the female migraine patients vs. female healthy controls comparison. Description: functional terms in the differentially expressed gene list accordingly to the Reactome database. NES – normalized enrichment score; FDR – false discovery rate

ID	Description	Enrichment Score	NES	p-Value	FDR	Proteins
R-HSA-372790	**Signaling by GPCR**	0.7902	2.0346	2.2 E-16	2.2 E-16	GRK1;NPBWR2;PTH2;RBP3;RGS11;WNT3A;WNT6
R-HSA-388396	**GPCR downstream signalling**	0.7926	1.8879	0.0013	0.0054	GRK1;NPBWR2;PTH2;RBP3;RGS11
R-HSA-162582	**Signal Transduction**	0.5561	1.7788	0.0011	0.0287	ARHGAP23;BCAR1;GRK1;KREMEN2;NPBWR2;PTH2;RBP3;RGS11;SPRED3;TERT;WNT3A;WNT6
R-HSA-418594	**G alpha (i) signalling events**	0.7991	1.7415	0.0044	0.0348	GRK1;NPBWR2;RBP3;RGS11
R-HSA-597592	**Post-translational protein modification**	0.7973	1.7864	0.0014	0.0350	ADAMTS16;B3GNT6;NEU4
R-HSA-392499	Metabolism of proteins	0.5793	1.6637	0.0197	0.0652	ADAMTS16;B3GNT6;NEU4;RGS11
R-HSA-500792	GPCR ligand binding	0.7076	1.6706	0.0167	0.0711	NPBWR2;PTH2;WNT3A;WNT6
R-HSA-195721	Signaling by WNT	0.7190	1.6154	0.0228	0.0985	KREMEN2;TERT;WNT3A;WNT6
R-HSA-382551	Transport of small molecules	0.7532	1.5199	0.0260	0.2131	ASIC4;HBB;SLC22A12;SLC44A5
R-HSA-201681	TCF dependent signaling in response to WNT	0.6734	1.4525	0.0550	0.2867	KREMEN2;TERT;WNT3A
R-HSA-8953854	Metabolism of RNA	-0.3195	-0.9810	0.4856	0.6769	CPSF7;POLR2A;RPL38;RPS13;RPS16;RPS21;RPS29;SNRPF;SRRM2
R-HSA-5663205	Infectious disease	-0.3206	-0.9976	0.4192	0.7542	CBL;CD9;EIF2AK2;PAK2;POLR2A;RPL38;RPS13;RPS16;RPS21;RPS29
R-HSA-212436	Generic Transcription Pathway	-0.3156	-1.0304	0.3957	0.8243	CBX5;CSNK2A2;GATAD2B;HIVEP3;MED12;PML;POLR2A;PRDM1;TCF7L2
R-HSA-114608	Platelet degranulation	-0.2482	-0.6570	0.8829	0.8749	CD9;CTSW;LGALS3BP;PF4;SERPINA1;SERPING1
R-HSA-76005	Response to elevated platelet cytosolic Ca2+	-0.2482	-0.6570	0.8829	0.8749	CD9;CTSW;LGALS3BP;PF4;SERPINA1;SERPING1

R-HSA-73857	RNA Polymerase II Transcription	-0.3045	-1.0823	0.3545	0.8968	CBX5;CPSF7;CSNK2A2;GATAD2B;HIVEP3;MED12;PML;POLR2A;PRDM1;SNRPF;TCF7L2;TNFRSF18
R-HSA-3700989	Transcriptional Regulation by TP53	-0.5186	-1.2623	0.2093	0.9775	CSNK2A2;GATAD2B;PML;POLR2A;PRDM1
R-HSA-199991	Membrane Trafficking	-0.4315	-1.2937	0.1654	1	CBL;DENND1C;DNAJC6;PAFAH1B1;SERPINA1;TBC1D3;TBC1D3C;UBQLN2
R-HSA-74160	Gene expression (Transcription)	-0.3149	-1.1348	0.2906	1	CBX5;CPSF7;CSNK2A2;GATAD2B;HIVEP3;MED12;PML;POLR2A;PRDM1;SNRPF;TCF7L2;TDRD9;TNFRSF18
R-HSA-5653656	Vesicle-mediated transport	-0.2174	-0.6938	0.8385	1	CBL;DENND1C;PAFAH1B1;SERPINA1;TBC1D3;TBC1D3C;UBQLN2

Supplementary Table 10: Gene set enrichment results of Peripheral Blood RNA-seq data of the female MwoA patients vs. female healthy controls comparison. Description: functional terms in the differentially expressed gene list accordingly to the Reactome database. NES – normalized enrichment score; FDR – false discovery rate

ID	Description	Enrichment score	NES	p-Value	FDR	Proteins
R-HSA-5663205	**Infectious disease**	-0.712962963	-2.370518622	2.2 E-16	2.2 E-16	CBL;EIF2AK2;PAK2;RPL30;RPL38;RPS12;RPS13;RPS15A;RPS21
R-HSA-71291	**Metabolism of amino acids and derivatives**	-0.703196347	-1.909314818	0.006536	0.006689	RPL30;RPL38;RPS12;RPS13;RPS15A;RPS21
R-HSA-72766	**Translation**	-0.703196347	-1.909314818	0.006536	0.006689	RPL30;RPL38;RPS12;RPS13;RPS15A;RPS21
R-HSA-6791226	**Major pathway of rRNA processing in the nucleolus and cytosol**	-0.703196347	-1.909314818	0.006536	0.006689	RPL30;RPL38;RPS12;RPS13;RPS15A;RPS21
R-HSA-72312	**rRNA processing**	-0.703196347	-1.909314818	0.006536	0.006689	RPL30;RPL38;RPS12;RPS13;RPS15A;RPS21
R-HSA-8868773	**rRNA processing in the nucleus and cytosol**	-0.703196347	-1.909314818	0.006536	0.006689	RPL30;RPL38;RPS12;RPS13;RPS15A;RPS21
R-HSA-9010553	**Regulation of expression of SLITs and ROBOs**	-0.703196347	-1.909314818	0.006536	0.006689	RPL30;RPL38;RPS12;RPS13;RPS15A;RPS21
R-HSA-2408522	**Selenoamino acid metabolism**	-0.703196347	-1.909314818	0.006536	0.006689	RPL30;RPL38;RPS12;RPS13;RPS15A;RPS21
R-HSA-156827	**L13a-mediated translational silencing of Ceruloplasmin expression**	-0.703196347	-1.909314818	0.006536	0.006689	RPL30;RPL38;RPS12;RPS13;RPS15A;RPS21
R-HSA-72613	**Eukaryotic Translation Initiation**	-0.703196347	-1.909314818	0.006536	0.006689	RPL30;RPL38;RPS12;RPS13;RPS15A;RPS21
R-HSA-388396	**GPCR downstream signalling**	0.761866428	1.795468335	0.001138	0.019206	NPBWR2;PTH1R;PTH2;RGS11
R-HSA-372790	Signaling by GPCR	0.643686573	1.655394144	0.007519	0.058603	NPBWR2;PTH1R;PTH2;RGS11;WNT3A;WNT6
R-HSA-500792	GPCR ligand binding	0.620370702	1.553788198	0.024149	0.151021	NPBWR2;PTH1R;PTH2;WNT3A;WNT6
R-HSA-373080	Class B/2 (Secretin family receptors)	0.709408486	1.503966102	0.026602	0.167929	PTH1R;PTH2;WNT3A;WNT6

R-HSA-418594	G alpha (i) signalling events	0.654462649	1.420408005	0.063549	0.268686	NPBWR2;RGS11
R-HSA-597592	Post-translational protein modification	0.495145137	1.344815414	0.079167	0.366472	ADAMTS16;B3GNT6;HIF3A;MSLN;NEU4
R-HSA-392499	Metabolism of proteins	0.443795906	1.294727349	0.103166	0.379378	ADAMTS16;B3GNT6;HIF3A;MSLN;NEU4;RGS11
R-HSA-1430728	Metabolism	0.493300508	1.306923128	0.11254	0.406349	DPEP1;NEU4
R-HSA-5173105	O-linked glycosylation	0.559090909	1.202170389	0.204082	0.562388	ADAMTS16;ADAMTSL2;B3GNT6;GALNT9;MUC6
R-HSA-162582	Signal Transduction	0.367372911	1.102910926	0.330303	0.652057	BCAR1;FGF22;GFRA3;GRIN1;KREMEN2;NPBWR2;PTH1R;PTH2;RGS11;SHC2;SPRED3;TERT;WNT3A;WNT6
